# Supplementary material for: Immunosenescence and vaccine efficacy revealed by immunometabolic analysis of SARS-CoV-2-specific cells in multiple sclerosis patients
Source: Nat Commun. 2024 Mar 29;15:2752. doi: 10.1038/s41467-024-47013-0 (PMC10980723; doi:10.1038/s41467-024-47013-0)
Supplement: Supplementary file 1 — Supplementary Information [file 41467_2024_47013_MOESM1_ESM.pdf]

Supplementary Information

Immunosenescence and vaccine efficacy revealed by immunometabolism of SARS-CoV-2-specific cells in vaccinated Multiple Sclerosis patients

De Biasi S, Lo Tartaro D, Neroni A, Rau M, Paschalidis N, Borella R, Santacroce E, Paolini A , Gibellini L, Ciobanu AL, Cuccorese M, Trenti T, Rubio I, Vitetta F, Cardi M, Argüello RJ, Ferraro D, Cossarizza A.

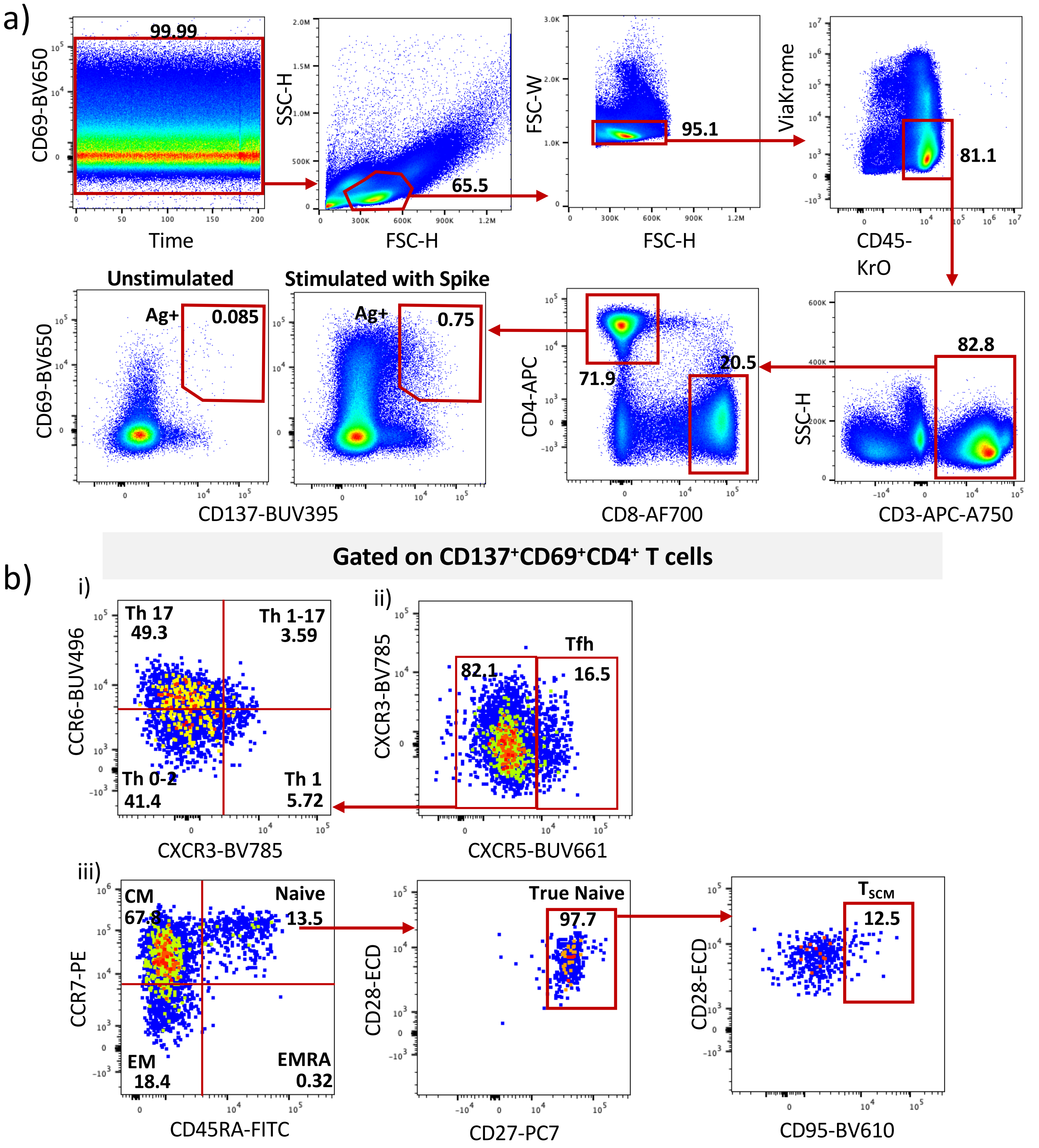

Supplementary Figure 1 Gating strategy for the identification and characterization of antigen-specific CD4+ T cells (AIM assay). **(a)** A gate was set on CD69 vs TIME plot, then in this population, a gate was set according to physical parameter (FSC and SSC. Further gating is done in an FSC-H and FSC-Width dot plot to eliminate doublets. On a bivariate plot of CD45 vs. ViaKrome (viability) select CD45+, ViaKrome− cells (viable cells). On a bivariate plot of CD3 vs SSC-H select CD3 T lymphocytes. CD4+ T cells was selected and the percentage of Antigen-Specific (CD69+ CD137+)T cells was quantified. **(b)** Gating strategy to identify and characterize i)T helper (Th), ii) circulating T follicular helper (cTfh), iii) Naive, true naive and T_{SCM} among Ag+CD4+ T cell populations. EM, effector memory; CM central memory; EMRA, terminally differentiated effector memory; T_{SCM} stem memory cell.

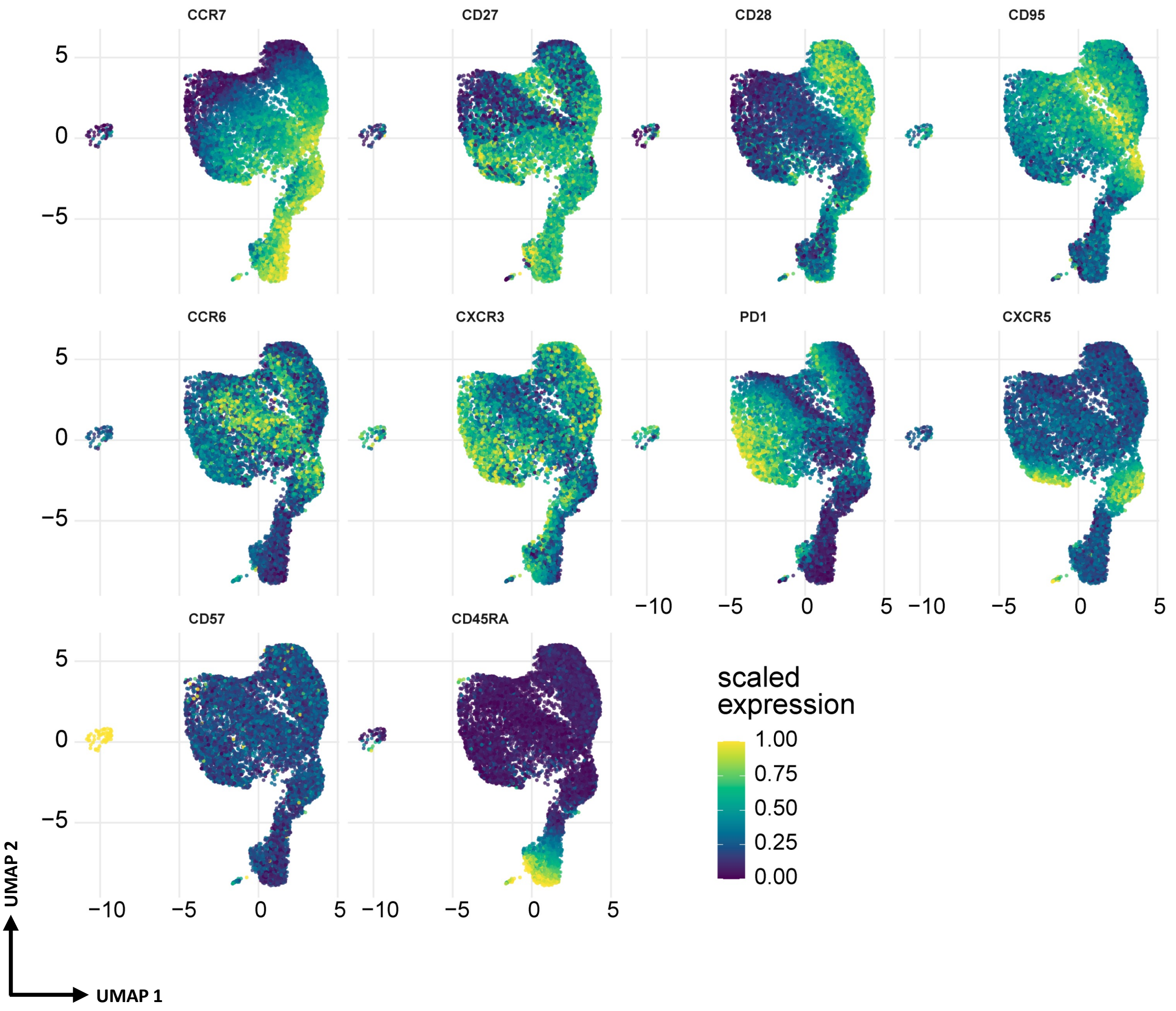

Supplementary Figure 2. Uniform Manifold Approximation and Projection (UMAP) plot shows the 2D spatial distribution of cells from 13 healthy donors (HD) vaccinated against SARS-CoV2 and 93 patients with multiple sclerosis undergoing different disease-modifying therapies (DMT) and vaccinated against COVID-19. UMAP graphs are colored by the expression of ten different markers used for unsupervised analysis of $CD4^{+}$ antigen specific T cells. Yellow: high expression. Dark blue: low expression.

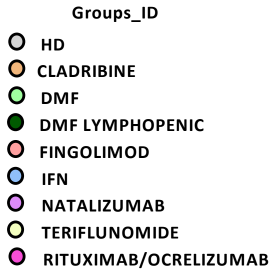

Naive

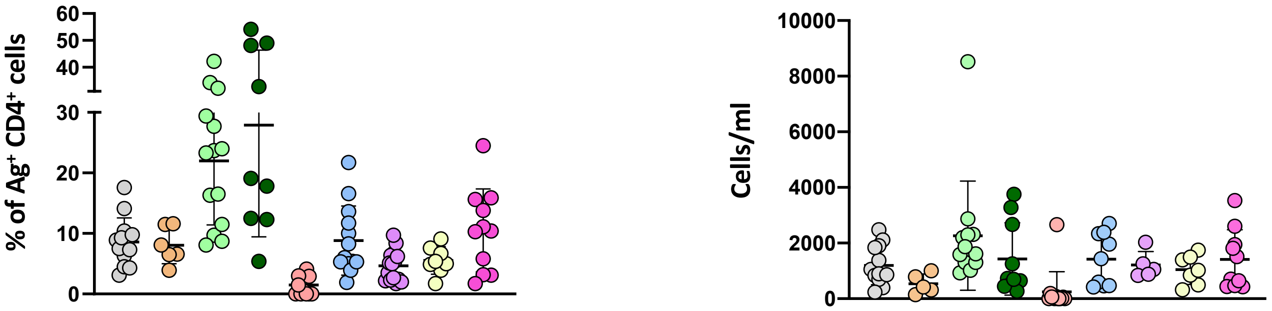

CD4+ T CELLS (N, %)	q-value	P-value
HD VS DMF	0.0307	0.0111
HD VS DMF LYMPHOPENIC	0.0452	0.0176
HD VS FINGOLIMOD	0.0013	0.0002
CLADRIBINE VS FINGOLIMOD	0.0137	0.0038
DMF VS FINGOLIMOD	<0.0001	<0.0001
DMF VS IFN	0.0180	0.0055
DMF VS NATALIZUMAB	<0.0001	<0.0001
DMF VS TERIFLUNOMIDE	0.0024	0.0005
DMF LYMPHOPENIC VS FINGOLIMOD	<0.0001	<0.0001
DMF LYMPHOPENIC VS IFN	0.0294	0.0098
DMF LYMPHOPENIC VS NATALIZUMAB	0.0002	<0.0001
DMF LYMPHOPENIC VS TERIFLUNOMIDE	0.0042	0.0011
FINGOLIMOD VS IFN	0.0024	0.0005
FINGOLIMOD VS RITUXIMAB/OCRELIZUMAB	0.0011	0.0002

CD4+ T CELLS (N, ml)	q-value	P-value
HD VS FINGOLIMOD	0.0045	0.0004
CLADRIBINE VS DMF	0.0193	0.0038
DMF VS FINGOLIMOD	<0.0001	<0.0001
DMF LYMPHOPENIC VS FINGOLIMOD	0.0083	0.0012
FINGOLIMOD VS IFN	0.0045	0.0004
FINGOLIMOD VS NATALIZUMAB	0.0193	0.0037
FINGOLIMOD VS TERIFLUNOMIDE	0.0303	0.0067
FINGOLIMOD VS RITUXIMAB/OCRELIZUMAB	0.0045	0.0005

Supplementary Figure 3A . Detailed statistical analysis of CD4+clusters obtained using FlowSOM. On the left, dot plots show the percentage of cells in different treated groups of patients and healthy donors (HD). On the right, dot plots show the absolute number of cells in different treated groups of patients and HD. Scatter plots show individual values; the central bar represents the mean ± SD. Kruskal–Wallis test (one-sided) with Benjamini–Hochberg correction for multiple comparisons. Tables display statistically significant q-value and individual p-value obtained. For all graphs: HD: healthy donors (N=13); Cladribine (N=6) DMF: Dimethyl Fumarate (N=14); DMF Lymphopenic: Dimethyl Fumarate Lymphopenic (N=9); Fingolimod (N=12); IFN: Interferon 1b (N=13); Natalizumab (N=15); Teriflunomide (N=8); Rituximab/Ocrelizumab (N=11).

T_{SCM}

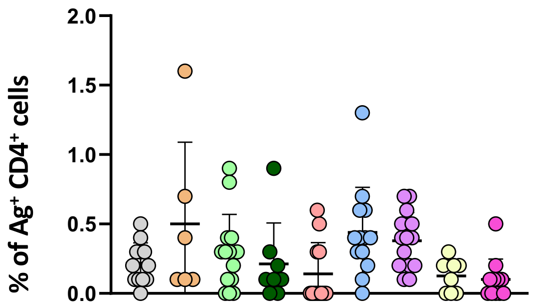

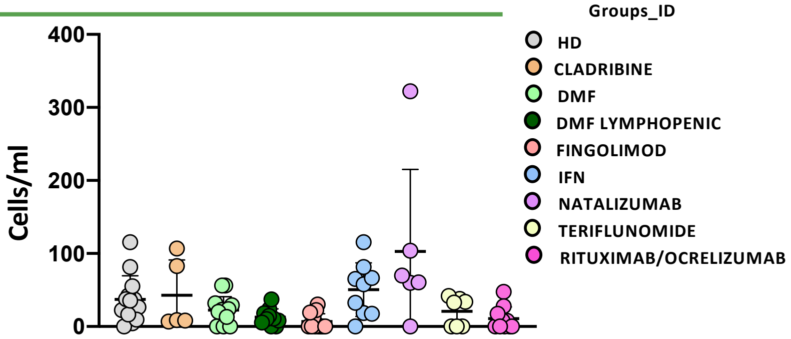

$CD4^+$ T CELLS (N, %)	q-value	P-value
FINGOLIMOD VS IFN	0.0159	0.0016
FINGOLIMOD VS NATALIZUMAB	0.0159	0.0018
IFN VS RITUXIMAB/OCRELIZUMAB	0.0159	0.0009
NATALIZUMAB VS RITUXIMAB/OCRELIZUMAB	0.0159	0.0009

$CD4^+$ T CELLS (N, ml)	q-value	P-value
HD VS FINGOLIMOD	0.0200	0.0018
FINGOLIMOD VS IFN	0.0114	0.0006
FINGOLIMOD VS NATALIZUMAB	0.0114	0.0003
IFN VS RITUXIMAB/OCRELIZUMAB	0.0352	0.0049
NATALIZUMAB VS RITUXIMAB/OCRELIZUMAB	0.0200	0.0022

CM Th_1

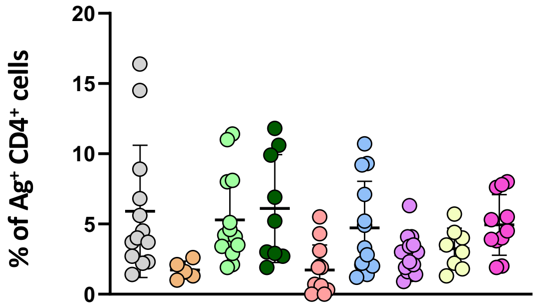

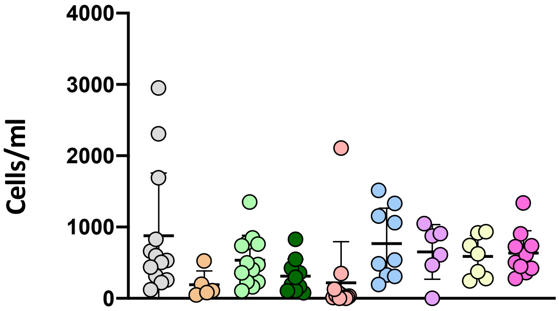

$CD4^+$ T CELLS (N, %)	q-value	P-value
HD VS CLADRIBINE	0.0254	0.0056
HD VS FINGOLIMOD	0.0094	0.0008
CLADRIBINE VS DMF	0.0254	0.0039
CLADRIBINE VS DMF LYMPHOPENIC	0.0254	0.0053
CLADRIBINE VS RITUXIMAB/OCRELIZUMAB	0.0254	0.0049
DMF VS FINGOLIMOD	0.0094	0.0004
DMF LYMPHOPENIC VS FINGOLIMOD	0.0095	0.0011
FINGOLIMOD VS IFN	0.0307	0.0077
FINGOLIMOD VS RITUXIMAB/OCRELIZUMAB	0.0094	0.0008

$CD4^+$ T CELLS (N, ml)	q-value	P-value
HD VS FINGOLIMOD	0.0026	0.0001
DMF VS FINGOLIMOD	0.0098	0.0013
FINGOLIMOD VS IFN	0.0026	0.0002
FINGOLIMOD VS NATALIZUMAB	0.0109	0.0018
FINGOLIMOD VS TERIFLUNOMIDE	0.0098	0.0014
FINGOLIMOD VS RITUXIMAB/OCRELIZUMAB	0.0026	0.0002

Supplementary Figure 3B. Detailed statistical analysis of CD4+clusters obtained using FlowSOM. On the left, dot plots show the percentage of cells in different treated groups of patients and healthy donors (HD). On the right, dot plots show the absolute number of cells in different treated groups of patients and HD. Scatter plots show individual values; the central bar represents the mean ± SD. Kruskal–Wallis test (one-sided) with Benjamini–Hochberg correction for multiple comparisons. Tables display statistically significant q-value and individual p-value obtained. For all graphs: HD: healthy donors (N=13); Cladribine (N=6) DMF: Dimethyl Fumarate (N=14); DMF Lymphopenic: Dimethyl Fumarate Lymphopenic (N=9); Fingolimod (N=12); IFN: Interferon 1b (N=13); Natalizumab (N=15); Teriflunomide (N=8); Rituximab/Ocrelizumab (N=11).

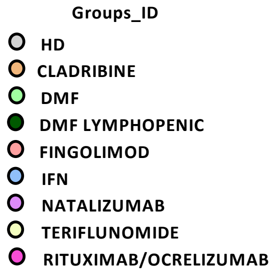

EM Th_2

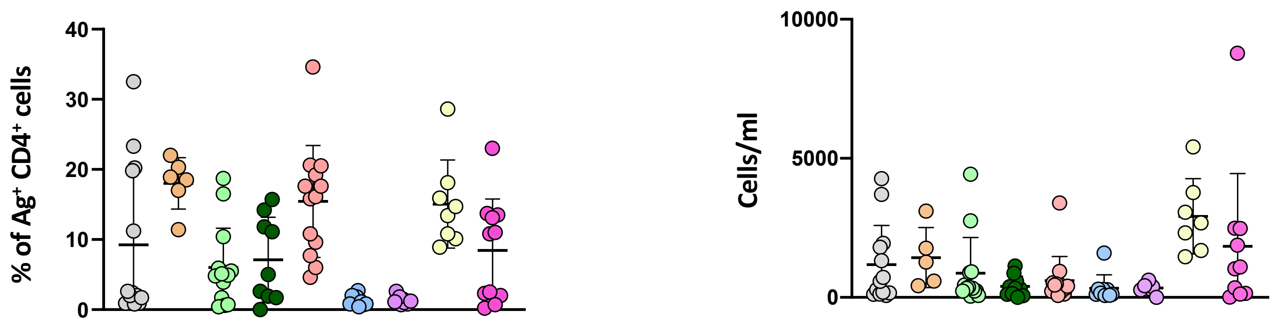

$CD4^+$ T CELLS (N, %)	q-value	P-value
CLADRIBINE VS DMF	0.0222	0.0043
CLADRIBINE VS IFN	0.0002	<0.0001
CLDRIBINE VS NATALIZUMAB	0.0002	<0.0001
DMF VS FINGOLIMOD	0.0282	0.0063
FINGOLIMOD VS IFN	0.0002	<0.0001
FINGOLIMOD VS NATALIZUMAB	0.0002	<0.0001
IFN VS TERIFLUNOMIDE	0.0012	0.0002
NATALIZUMAB VS TERIFLUNOMIDE	0.0012	0.0002

$CD4^+$ T CELLS (N, ml)	q-value	P-value
HD VS TERIFLUNOMIDE	0.0490	0.0095
CLADRIBINE VS IFN	0.0476	0.0079
DMF VS TERIFLUNOMIDE	0.0246	0.0034
DMF LYMPHOPENIC VS TERIFLUNOMIDE	0.0083	0.0005
FINGOLIMOD VS TERIFLUNOMIDE	0.0246	0.0022
IFN VS TERIFLUNOMIDE	0.0024	<0.0001
NATALIZUMAB VS TERIFLUNOMIDE	0.0246	0.0034

Supplementary Figure 3C. Detailed statistical analysis of CD4+clusters obtained using FlowSOM. On the left, dot plots show the percentage of cells in different treated groups of patients and healthy donors (HD). On the right, dot plots show the absolute number of cells in different treated groups of patients and HD. Scatter plots show individual values; the central bar represents the mean ± SD. Kruskal–Wallis test (one-sided) with Benjamini–Hochberg correction for multiple comparisons. Tables display statistically significant q-value and individual p-value obtained. For all graphs: HD: healthy donors (N=13); Cladribine (N=6) DMF: Dimethyl Fumarate (N=14); DMF Lymphopenic: Dimethyl Fumarate Lymphopenic (N=9); Fingolimod (N=12); IFN: Interferon 1b (N=13); Natalizumab (N=15); Teriflunomide (N=8); Rituximab/Ocrelizumab (N=11).

Tfh

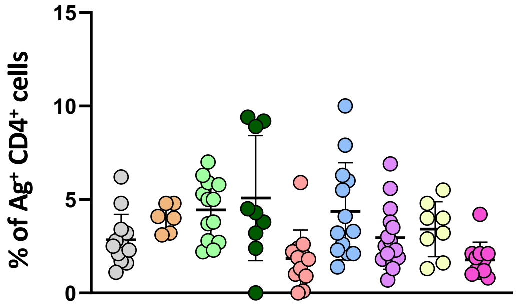

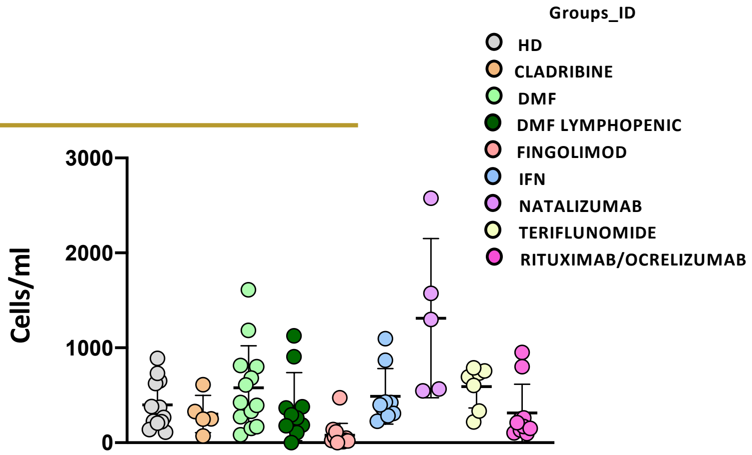

CD4⁺ T CELLS (N, %)	q-value	P-value
CLADRIBINE VS FINGOLIMOD	0.0174	0.0039
CLADRIBINE VS RITUXIMAB/OCRELIZUMAB	0.0116	0.0023
DMF VS FINGOLIMOD	0.0031	0.0002
DMF VS RITUXIMAB/OCRELIZUMAB	0.0031	<0.0001
DMF LYMPHOPENIC VS FINGOLIMOD	0.0088	0.0012
DMF LYMPHOPENIC VS RITUXIMAB/OCRELIZUMAB	0.0078	0.0007
FINGOLIMOD VS IFN	0.0107	0.0018
IFN VS RITUXIMAB/OCRELIZUMAB	0.0083	0.0009

CD4⁺ T CELLS (N, ml)	q-value	P-value
HD VS FINGOLIMOD	0.0046	0.0006
DMF VS FINGOLIMOD	0.0004	<0.0001
DMF LYMPHOPENIC VS FINGOLIMOD	0.0320	0.0062
FINGOLIMOD VS IFN	0.0009	0.0001
FINGOLIMOD VS NATALIZUMAB	<0.0001	<0.0001
FINGOLIMOD VS TERIFLUNOMIDE	0.0006	<0.0001
NATALIZUMAB VS RITUXIMAB/OCRELIZUMAB	0.0240	0.0040

EM Th_1 $PD1^+$

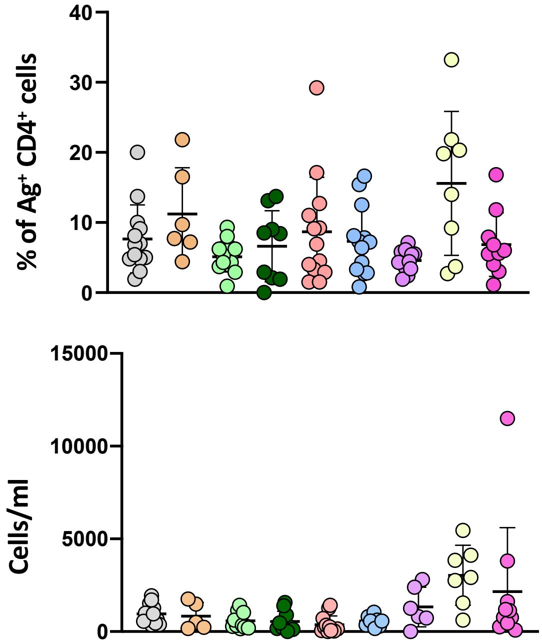

CD4⁺ T CELLS (N, ml)	q-value	P-value
HD VS FINGOLIMOD	0.0202	0.0022
DMF VS TERIFLUNOMIDE	0.0141	0.0012
DMF LYMPHOPENIC VS TERIFLUNOMIDE	0.0087	0.0005
FINGOLIMOD VS TERIFLUNOMIDE	0.0003	<0.0001
FINGOLIMOD VS RITUXIMAB/OCRELIZUMAB	0.0244	0.0041
IFN VS TERIFLUNOMIDE	0.0244	0.0037

Supplementary Figure 3D. Detailed statistical analysis of CD4+clusters obtained using FlowSOM. On the left, dot plots show the percentage of cells in different treated groups of patients and healthy donors (HD). On the right, dot plots show the absolute number of cells in different treated groups of patients and HD. Scatter plots show individual values; the central bar represents the mean ± SD. Kruskal–Wallis test (one-sided) with Benjamini–Hochberg correction for multiple comparisons. Tables display statistically significant q-value and individual p-value obtained. For all graphs: HD: healthy donors (N=13); Cladribine (N=6) DMF: Dimethyl Fumarate (N=14); DMF Lymphopenic: Dimethyl Fumarate Lymphopenic (N=9); Fingolimod (N=12); IFN: Interferon 1b (N=13); Natalizumab (N=15); Teriflunomide (N=8); Rituximab/Ocrelizumab (N=11).

CM CXCR5^{+}

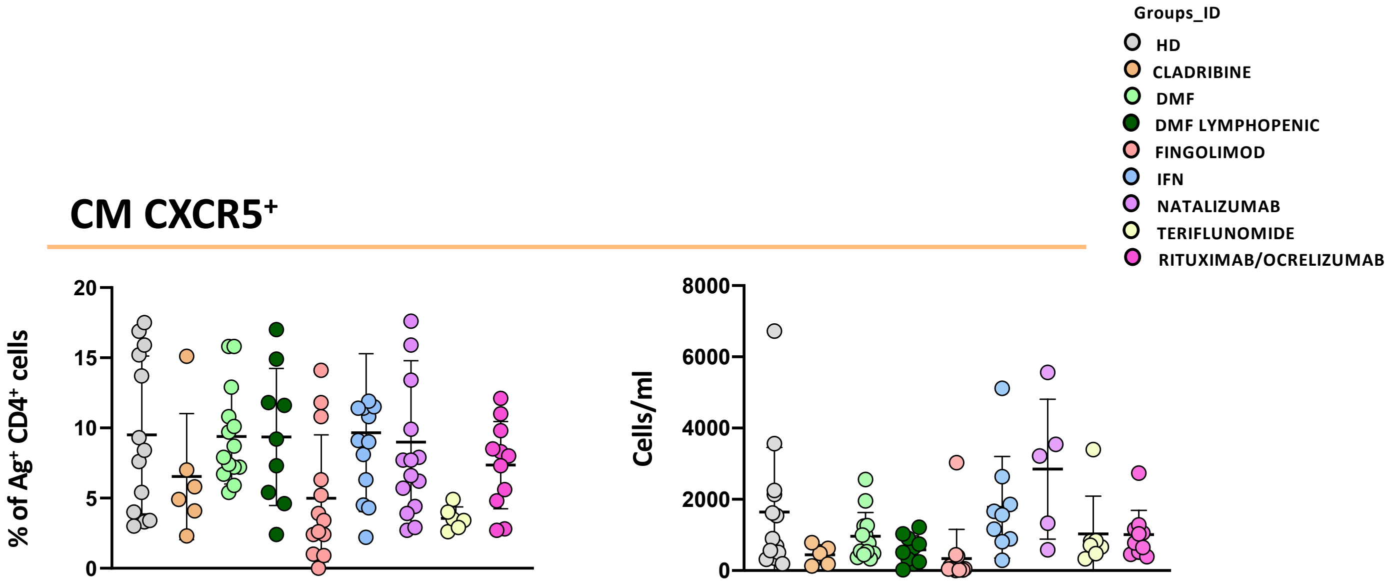

CD4^{+} T CELLS (N, ml)	q-value	P-value
HD VS FINGOLIMOD	0.0017	0.0001
CLADRIBINE VS NATALIZUMAB	0.0424	0.0083
DMF VS FINGOLIMOD	0.0074	0.0010
DMF LYMPHOPENIC VS NATALIZUMAB	0.0424	0.0094
FINGOLIMOD VS IFN	0.0004	<0.0001
FINGOLIMOD VS NATALIZUMAB	0.000.	<0.0001
FINGOLIMOD VS TERIFLUNOMIDE	0.0424	0.0085
FINGOLIMOD VS RITUXIMAB/OCRELIZUMAB	0.0074	0.0009

Supplementary Figure 3E . Detailed statistical analysis of CD4+clusters obtained using FlowSOM. On the left, dot plots show the percentage of cells in different treated groups of patients and healthy donors (HD). On the right, dot plots show the absolute number of cells in different treated groups of patients and HD. Scatter plots show individual values; the central bar represents the mean ± SD. Kruskal–Wallis test (one-sided) with Benjamini–Hochberg correction for multiple comparisons. Tables display statistically significant q-value and individual p-value obtained. For all graphs: HD: healthy donors (N=13); Cladribine (N=6) DMF: Dimethyl Fumarate (N=14); DMF Lymphopenic: Dimethyl Fumarate Lymphopenic (N=9); Fingolimod (N=12); IFN: Interferon 1b (N=13); Natalizumab (N=15); Teriflunomide (N=8); Rituximab/Ocrelizumab (N=11).

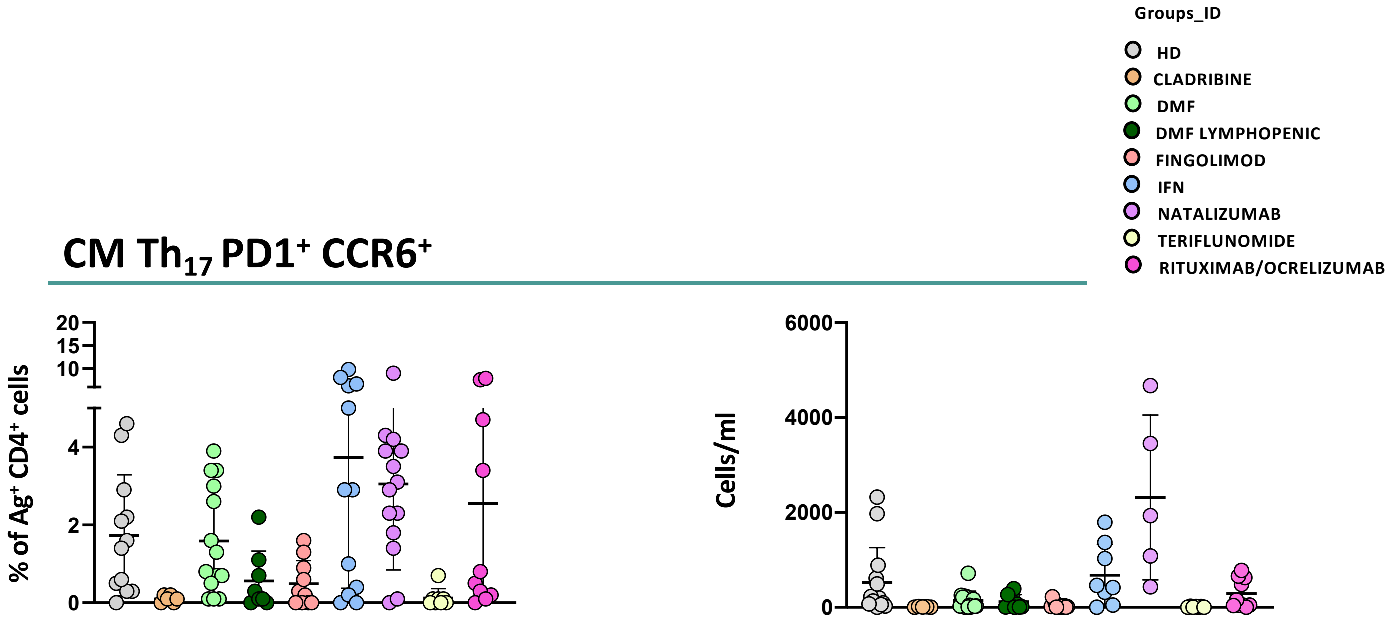

CD4+ T CELLS (N, %)	q-value	P-value
HD VS CLADRIBINE	0.0485	0.0163
HD VS TERIFLUNOMIDE	0.0262	0.0050
CLADRIBINE VS DMF	0.0485	0.0175
CLADRIBINE VS IFN	0.0172	0.0019
CLADRIBINE VS NATALIZUMAB	0.0101	0.0008
DMF VS TERIFLUNOMIDE	0.0262	0.0051
DMF LYMPHOPENIC VS IFN	0.0485	0.0149
DMF LYMPHOPENIC VS NATALIZUMAB	0.0281	0.0072
FINGOLIMOD VS IFN	0.0262	0.0058
FINGOLIMOD VS NATALIZUMAB	0.0172	0.0024
IFN VS TERIFLUNOMIDE	0.0060	0.0003
NATALIZUMAB VS TERIFLUNOMIDE	0.0041	0.0001
TERIFLUNOMIDE VS RITUXIMAB/OCRELIZUMAB	0.0281	0.0078

CD4+ T CELLS (N, ml)	q-value	P-value
HD VS FINGOLIMOD	0.0098	0.0026
HD VS TERIFLUNOMIDE	0.0041	0.0006
HD VS CLADRIBINA	0.0088	0.0017
CLADRIBINE VS IFN	0.0084	0.0014
CLADRIBINE VS NATALIZUMAB	0.0008	<0.0001
CLADRIBINE VS RITUXIMAB/OCRELIZUMAB	0.0250	0.0090
DMF VS NATALIZUMAB	0.0137	0.0042
DMF LYMPHOPENIC VS NATALIZUMAB	0.0098	0.0024
FINGOLIMOD VS IFN	0.0098	0.0027
FINGOLIMOD VS NATALIZUMAB	0.0008	<0.0001
IFN VS TERIFLUNOMIDE	0.0041	0.0006
NATALIZUMAB VS TERIFLUNOMIDE	0.0006	<0.0001
TERIFLUNOMIDE VS RITUXIMAB/OCRELIZUMAB	0.0141	0.0047

Supplementary Figure 3F. Detailed statistical analysis of CD4+clusters obtained using FlowSOM. On the left, dot plots show the percentage of cells in different treated groups of patients and healthy donors (HD). On the right, dot plots show the absolute number of cells in different treated groups of patients and HD. Scatter plots show individual values; the central bar represents the mean ± SD. Kruskal–Wallis test (one-sided) with Benjamini–Hochberg correction for multiple comparisons. Tables display statistically significant q-value and individual p-value obtained. For all graphs: HD: healthy donors (N=13); Cladribine (N=6) DMF: Dimethyl Fumarate (N=14); DMF Lymphopenic: Dimethyl Fumarate Lymphopenic (N=9); Fingolimod (N=12); IFN: Interferon 1b (N=13); Natalizumab (N=15); Teriflunomide (N=8); Rituximab/Ocrelizumab (N=11).

TM Th$_1$ CXCR3$^+$

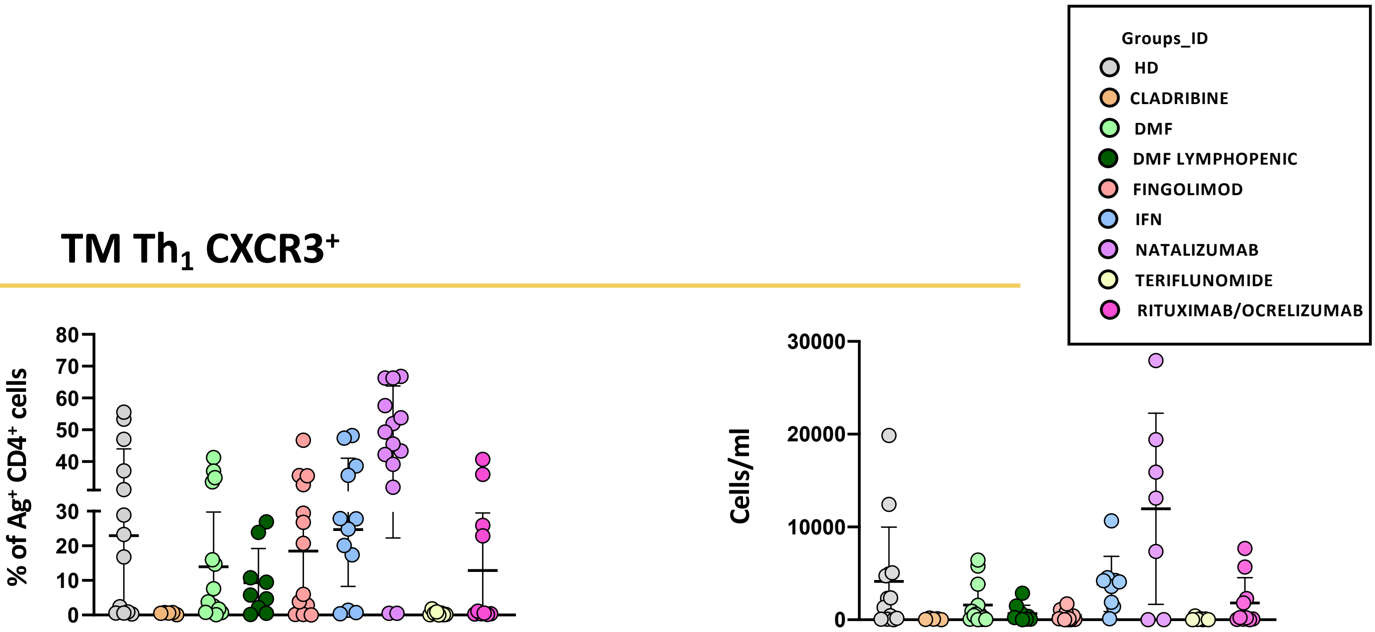

CD4+ T CELLS (N, %)	q-value	P-value
HD VS TERIFLUNOMIDE	0.0138	0.0020
CLADRIBINE VS IFN	0.0384	0.0096
CLADRIBINE VS NATALIZUMAB	0.0017	<0.0001
DMF VS NATALIZUMAB	0.0215	0.0042
DMF VS TERIFLUNOMIDE	0.0433	0.0132
DMF LYMPHOPENIC VS NATALIZUMAB	0.0138	0.0023
FINGOLIMOD VS NATALIZUMAB	0.0322	0.0072
FINGOLIMOD VS TERIFLUNOMIDE	0.0394	0.0110
IFN VS TERIFLUNOMIDE	0.0071	0.0008
NATALIZUMAB VS TERIFLUNOMIDE	<0.0001	<0.0001
NATALIZUMAB VS RITUXIMAB/OCRELIZUMAB	0.0071	0.0006

CD4+ T CELLS (N, ml)	q-value	P-value
HD VS TERIFLUNOMIDE	0.0334	0.0037
CLADRIBINE VS IFN	0.0334	0.0019
CLADRIBINE VS NATALIZUMAB	0.0416	0.0077
FINGOLIMOD VS IFN	0.0433	0.0060
IFN VS TERIFLUNOMIDE	0.0195	0.0005
NATALIZUMAB VS TERIFLUNOMIDE	0.0334	0.0032

Supplementary Figure 3G Detailed statistical analysis of CD4+clusters obtained using FlowSOM. On the left, dot plots show the percentage of cells in different treated groups of patients and healthy donors (HD). On the right, dot plots show the absolute number of cells in different treated groups of patients and HD. Scatter plots show individual values; the central bar represents the mean ± SD. Kruskal–Wallis test (one-sided) with Benjamini–Hochberg correction for multiple comparisons. Tables display statistically significant q-value and individual p-value obtained. For all graphs: HD: healthy donors (N=13); Cladribine (N=6) DMF: Dimethyl Fumarate (N=14); DMF Lymphopenic: Dimethyl Fumarate Lymphopenic (N=9); Fingolimod (N=12); IFN: Interferon 1b (N=13); Natalizumab (N=15); Teriflunomide (N=8); Rituximab/Ocrelizumab (N=11).

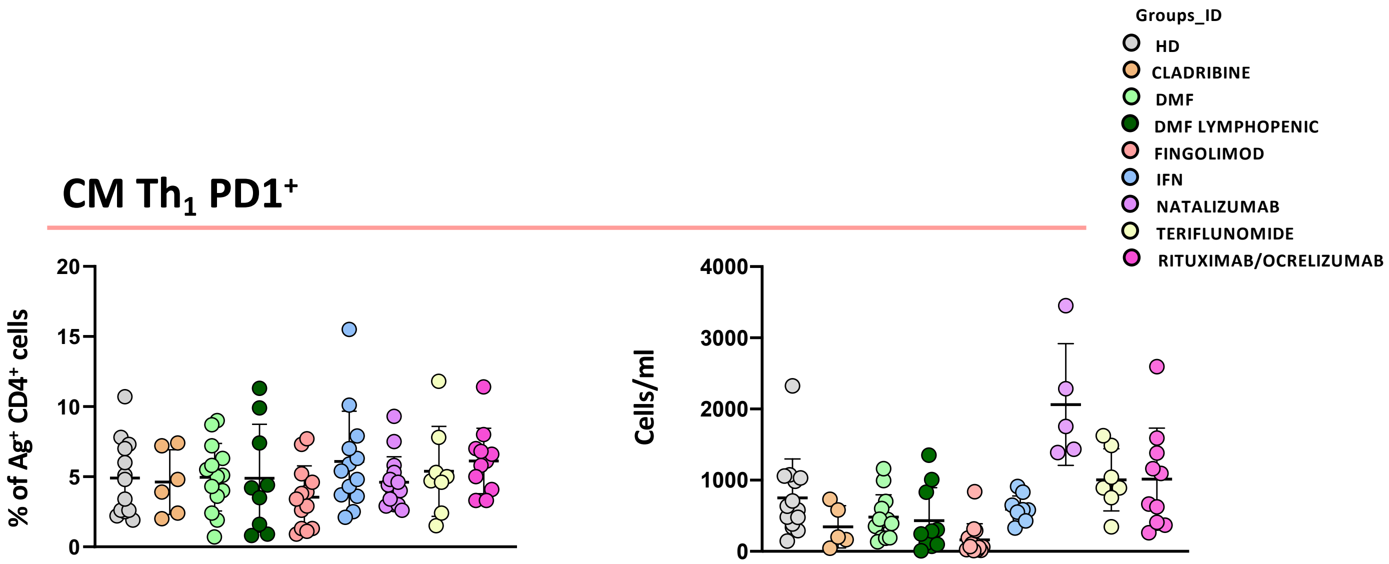

$CD4^+$ T CELLS (N, ml)	q-value	P-value
HD VS FINGOLIMOD	0.0020	0.0002
CLADRIBINE VS NATALIZUMAB	0.0065	0.0011
DMF VS FINGOLIMOD	0.0482	0.0149
DMF VS NATALIZUMAB	0.0068	0.0013
DMF LYMPHOPENIC VS NATALIZUMAB	0.0020	0.0003
DMF LYMPHOPENIC VS TERIFLUNOMIDE	0.0370	0.0092
DMF LYMPHOPENIC VS RITUXIMAB/OCRELIZUMAB	0.0480	0.0145
FINGOLIMOD VS IFN	0.0120	0.0027
FINGOLIMOD VS NATALIZUMAB	<0.0001	<0.0001
FINGOLIMOD VS TERIFLUNOMIDE	0.0004	<0.0001
FINGOLIMOD VS RITUXIMAB/OCRELIZUMAB	0.0004	<0.0001
IFN VS NATALIZUMAB	0.0482	0.0161

Supplementary Figure 3H Detailed statistical analysis of CD4+clusters obtained using FlowSOM. On the left, dot plots show the percentage of cells in different treated groups of patients and healthy donors (HD). On the right, dot plots show the absolute number of cells in different treated groups of patients and HD. Scatter plots show individual values; the central bar represents the mean ± SD. Kruskal–Wallis test (one-sided) with Benjamini–Hochberg correction for multiple comparisons. Tables display statistically significant q-value and individual p-value obtained. For all graphs: HD: healthy donors (N=13); Cladribine (N=6) DMF: Dimethyl Fumarate (N=14); DMF Lymphopenic: Dimethyl Fumarate Lymphopenic (N=9); Fingolimod (N=12); IFN: Interferon 1b (N=13); Natalizumab (N=15); Teriflunomide (N=8); Rituximab/Ocrelizumab (N=11).

CM Th$_{1-17}$

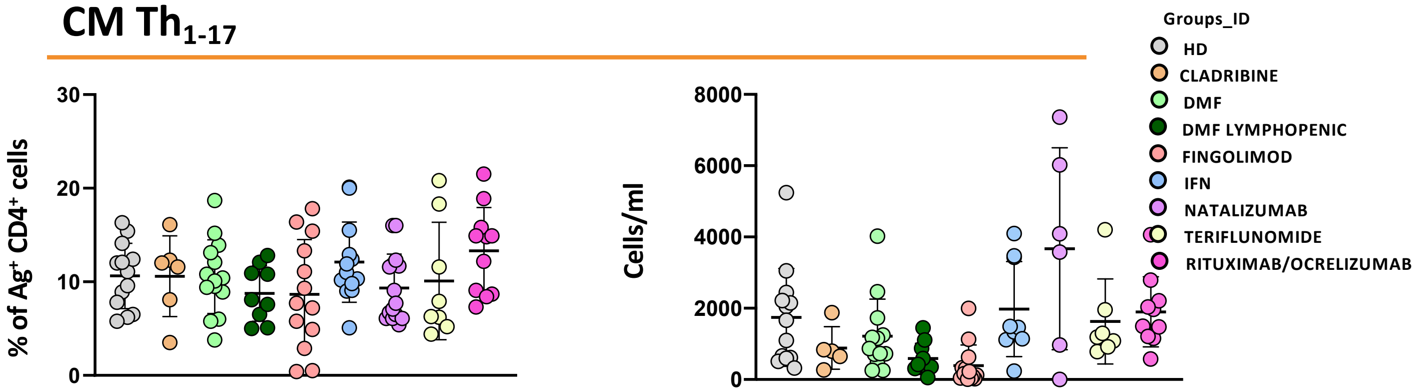

CD4+ T CELLS (N, ml)	q-value	P-value
HD VS FINGOLIMOD	0.0039	0.0004
DMF VS FINGOLIMOD	0.0400	0.0100
DMF LYMPHOPENIC VS IFN	0.0400	0.0097
DMF LYMPHOPENIC VS NATALIZUMAB	0.0334	0.0065
FINGOLIMOD VS IFN	0.0032	0.0003
FINGOLIMOD VS NATALIZUMAB	0.0032	0.0003
FINGOLIMOD VS TERIFLUNOMIDE	0.0168	0.0023
FINGOLIMOD VS RITUXIMAB/OCRELIZUMAB	0.0016	<0.0001

CM Th$_2$

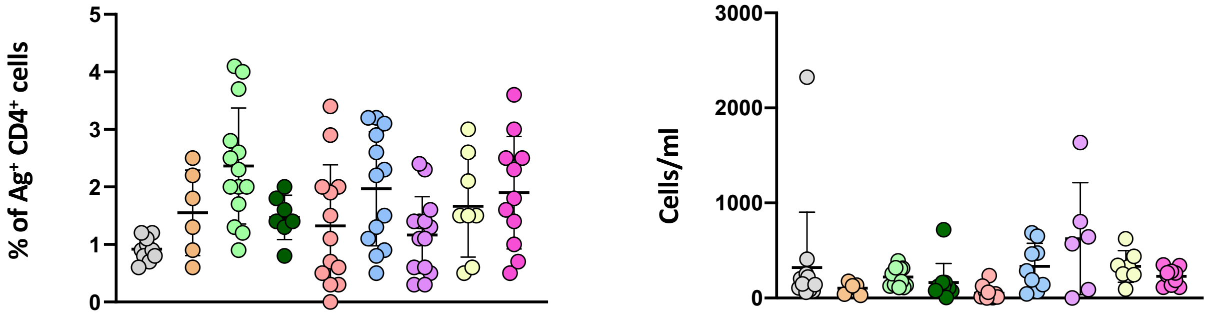

CD4+ T CELLS (N, %)	q-value	P-value
HD VS DMF	0.0085	0.0002
DMF VS NATALIZUMAB	0.0270	0.0015

CD4+ T CELLS (N, ml)	q-value	P-value
HD VS FINGOLIMOD	0.0117	0.0020
DMF VS FINGOLIMOD	0.0017	0.0002
FINGOLIMOD VS IFN	0.0017	0.0001
FINGOLIMOD VS NATALIZUMAB	0.0024	0.0003
FINGOLIMOD VS TERIFLUNOMIDE	0.0017	<0.0001
FINGOLIMOD VS RITUXIMAB/OCRELIZUMAB	0.0017	0.0002

Supplementary Figure 3l Detailed statistical analysis of CD4+clusters obtained using FlowSOM. On the left, dot plots show the percentage of cells in different treated groups of patients and healthy donors (HD). On the right, dot plots show the absolute number of cells in different treated groups of patients and HD. Scatter plots show individual values; the central bar represents the mean ± SD. Kruskal–Wallis test (one-sided) with Benjamini–Hochberg correction for multiple comparisons. Tables display statistically significant q-value and individual p-value obtained. For all graphs: HD: healthy donors (N=13); Cladribine (N=6) DMF: Dimethyl Fumarate (N=14); DMF Lymphopenic: Dimethyl Fumarate Lymphopenic (N=9); Fingolimod (N=12); IFN: Interferon 1b (N=13); Natalizumab (N=15); Teriflunomide (N=8); Rituximab/Ocrelizumab (N=11).

CM Th_{17}

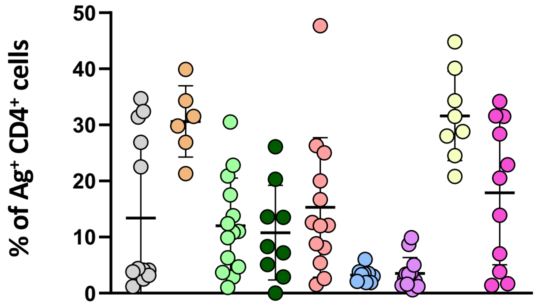

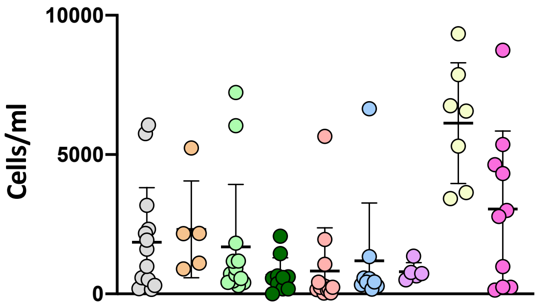

CD4+ T CELLS (N, %)	q-value	P-value
HD VS CLADRIBINE	0.0312	0.0102
HD VS NATALIZUMAB	0.0443	0.0209
HD VS TERIFLUNOMIDE	0.0231	0.0045
CLADRIBINE VS DMF	0.0326	0.0128
CLADRIBINE VS DMF LYMPHOPENIC	0.0312	0.0104
CLADRIBINE VS IFN	0.0005	<0.0001
CLADRIBINE VS NATALIZUMAB	0.0001	<0.0001
DMF VS NATALIZUMAB	0.0326	0.0127
DMF VS TERIFLUNOMIDE	0.0232	0.0058
DMF LYMPHOPENIC VS TERIFLUNOMIDE	0.0232	0.0052
FINGOLIMOD VS IFN	0.0326	0.0136
FINGOLIMOD VS NATALIZUMAB	0.0231	0.0042
FINGOLIMOD VS TERIFLUNOMIDE	0.0413	0.0183
IFN VS TERIFLUNOMIDE	0.0001	<0.0001
IFN VS RITUXIMAB/OCRELIZUMAB	0.0248	0.0069
NATALIZUMAB VS TERIFLUNOMIDE	<0.0001	<0.0001
NATALIZUMAB VS RITUXIMAB/OCRELIZUMAB	0.0143	0.0020

CD4+ T CELLS (N, ml)	q-value	P-value
HD VS TERIFLUNOMIDE	0.0386	0.0053
CLADRIBINE VS FINGOLIMOD	0.0386	0.0073
DMF VS TERIFLUNOMIDE	0.0386	0.0057
DMF LYMPHOPENIC VS TERIFLUNOMIDE	0.0029	0.0002
FINGOLIMOD VS TERIFLUNOMIDE	0.0001	<0.0001
FINGOLIMOD VS RITUXIMAB/OCRELIZUMAB	0.0386	0.0075
IFN VS TERIFLUNOMIDE	0.0067	0.0006

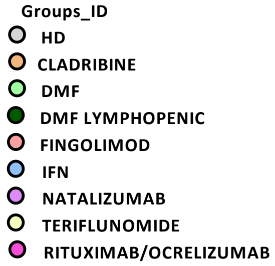

Supplementary Figure 3L Detailed statistical analysis of CD4+clusters obtained using FlowSOM. On the left, dot plots show the percentage of cells in different treated groups of patients and healthy donors (HD). On the right, dot plots show the absolute number of cells in different treated groups of patients and HD. Scatter plots show individual values; the central bar represents the mean ± SD. Kruskal–Wallis test (one-sided) with Benjamini–Hochberg correction for multiple comparisons. Tables display statistically significant q-value and individual p-value obtained. For all graphs: HD: healthy donors (N=13); Cladribine (N=6) DMF: Dimethyl Fumarate (N=14); DMF Lymphopenic: Dimethyl Fumarate Lymphopenic (N=9); Fingolimod (N=12); IFN: Interferon 1b (N=13); Natalizumab (N=15); Teriflunomide (N=8); Rituximab/Ocrelizumab (N=11).

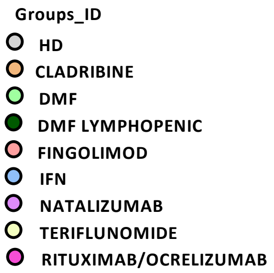

CM Th_1 $CD57^+$

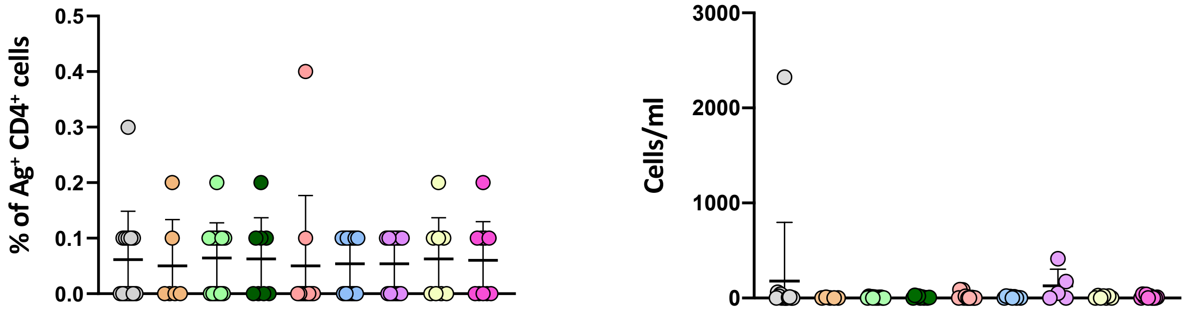

EM Th_1 $CD57^+$

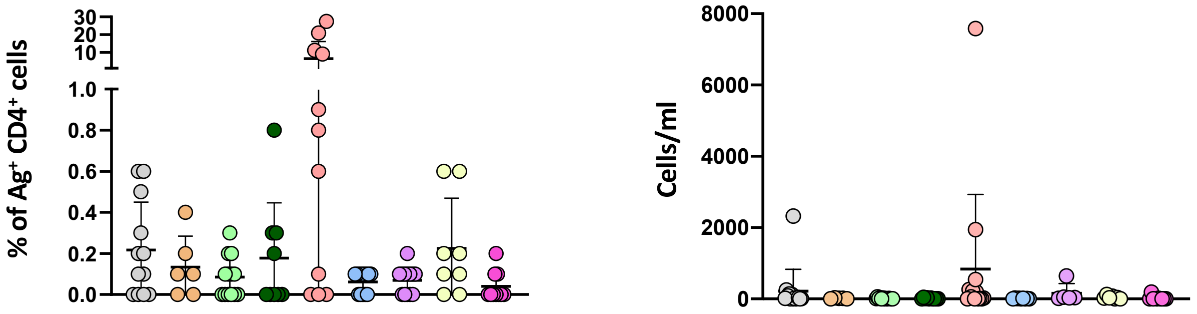

Supplementary Figure 3M Detailed statistical analysis of CD4+clusters obtained using FlowSOM. On the left, dot plots show the percentage of cells in different treated groups of patients and healthy donors (HD). On the right, dot plots show the absolute number of cells in different treated groups of patients and HD. Scatter plots show individual values; the central bar represents the mean ± SD. Kruskal–Wallis test (one-sided) with Benjamini–Hochberg correction for multiple comparisons. Tables display statistically significant q-value and individual p-value obtained. For all graphs: HD: healthy donors (N=13); Cladribine (N=6) DMF: Dimethyl Fumarate (N=14); DMF Lymphopenic: Dimethyl Fumarate Lymphopenic (N=9); Fingolimod (N=12); IFN: Interferon 1b (N=13); Natalizumab (N=15); Teriflunomide (N=8); Rituximab/Ocrelizumab (N=11).

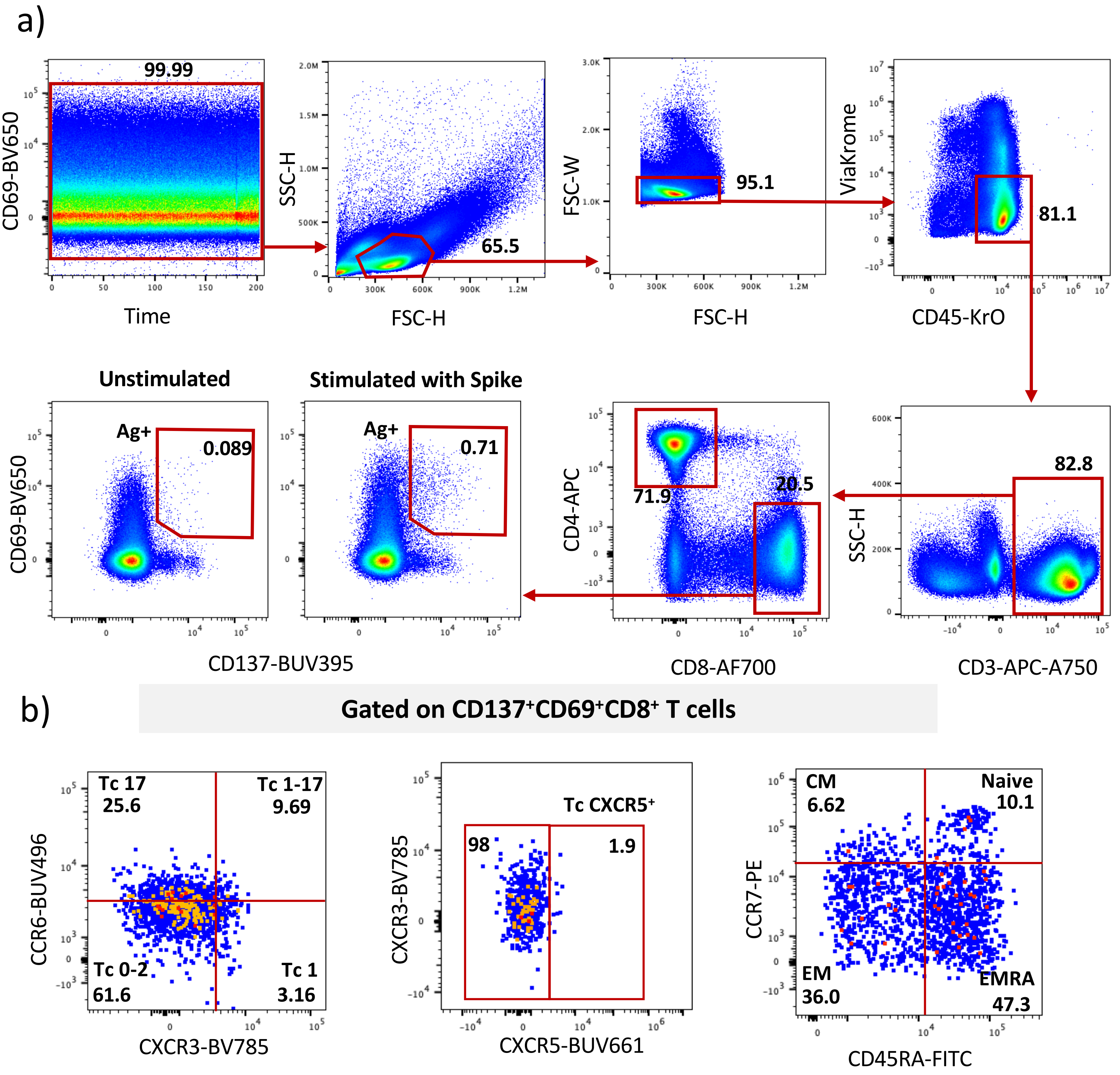

Supplementary Figure 4. Gating strategy for the identification and characterization of antigen-specific $CD8^+$ T cells (AIM assay). **(a)** A gate was set on CD69 vs TIME plot, then in this population, a gate was set according to physical parameter (FSC and SSC. Further gating is done in an FSC-H and FSC-Width dot plot to eliminate doublets. On a bivariate plot of CD45 vs. ViaKrome (viability) select $CD45^+$, $ViaKrome^-$ cells (viable cells). On a bivariate plot of CD3 vs SSC-H select CD3 T lymphocytes. $CD4^+$ T cells was selected and the percentage of Antigen-Specific ($CD69^+$ $CD137^+$)T cells was quantified. **(b)** Gating strategy to identify and characterize i)T cytotoxic (Tc), ii) Tc $CXCR5^+$, iii) Naive, true naive and T_{SCM} among Ag^+CD4^+ T cell populations. EM, effector memory; CM central memory; EMRA, terminally differentiated effector memory; T_{SCM} stem memory cell.

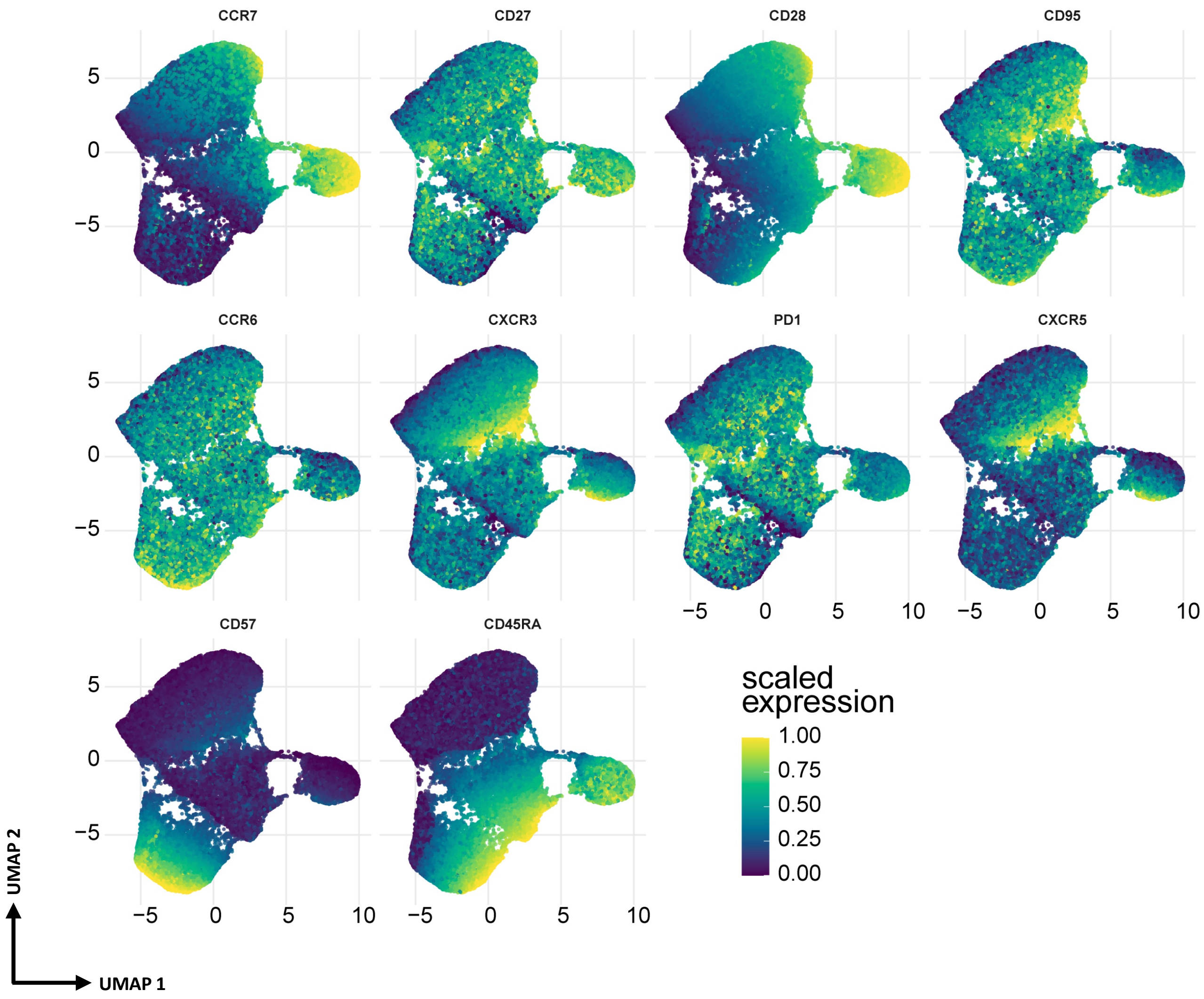

Supplementary Figure 5. Uniform Manifold Approximation and Projection (UMAP) plot shows the 2D spatial distribution of cells from 28 heallthy donors vaccinated against SARS-CoV2 and 106 patients with multiple sclerosis undergoing different disease-modifying therapies (DMT) and vaccinated against COVID-19. UMAP graphs colored by the expression of 10 markers used for $CD8^+$ antigen specific T cell. Blue represents lower expression while yellow represent higher expression.

Naive Tc_0

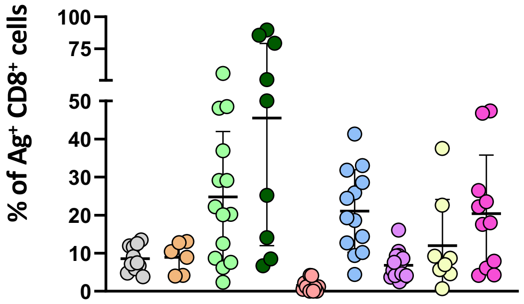

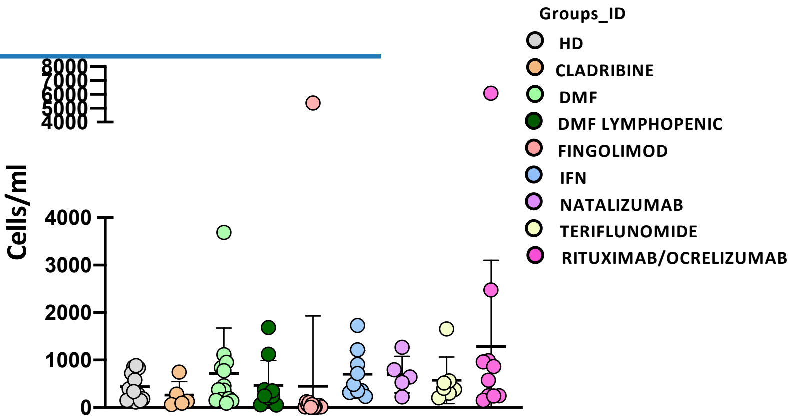

CD8+ T CELLS (N, %)	q-value	p-value
HD vs. DMF LYMPHO	0.0234	0.0065
HD vs. FINGOLIMOD	0.0164	0.0036
CLADRIBINE vs. FINGOLIMOD	0.0399	0.0122
DMF vs. FINGOLIMOD	<0.0001	<0.0001
DMF vs. NATALIZUMAB	0.0097	0.0019
DMF LYMPHO vs. FINGOLIMOD	<0.0001	<0.0001
DMF LYMPHO vs. NATALIZUMAB	0.0020	0.0003
DMF LYMPHO vs. TERFLUONOMIDE	0.0497	0.0166
FINGOLIMOD vs. IFN	<0.0001	<0.0001
FINGOLIMOD vs. TERFLUONOMIDE	0.0211	0.0053
FINGOLIMOD vs. RITUXIMAB/OCRELIZUMAB	0.0001	<0.0001
IFN vs. NATALIZUMAB	0.0086	0.0014

CD8+ T CELLS (N, ml)	q-value	p-value
HD VS FINGOLIMOD	0.0094	0.0016
DMF VS FINGOLIMOD	0.0053	0.0004
DMF LYMPHOPENIC VS FINGOLIMOD	0.0432	0.0084
FINGOLIMOD VS IFN	0.0011	<0.0001
FINGOLIMOD VS NATALIZUMAB	0.0056	0.0006
FINGOLIMOD VS TERIFLUNOMIDE	0.0073	0.0010
FINGOLIMOD VS RITUXIMAB/OCRELIZUMAB	0.0010	<0.0001

CM Tc_{0-2}

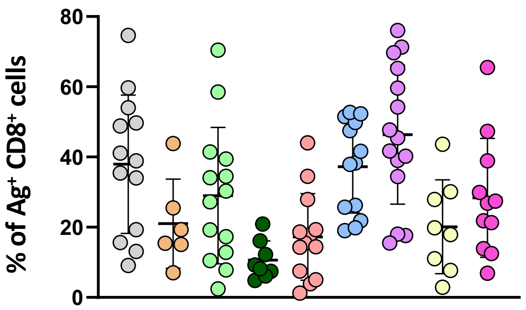

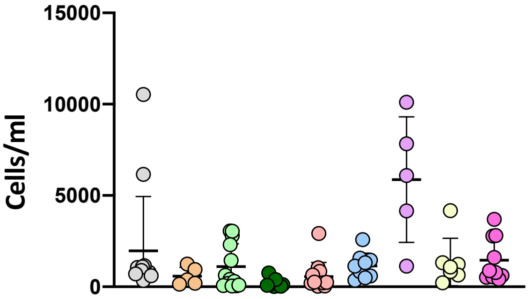

CD8+ T CELLS (N, %)	q-value	p-value
HD vs. DMF LYMPHO	0.0053	0.0006
HD vs. FINGOLIMOD	0.0267	0.0052
CLADRIBINE vs. NATALIZUMAB	0.0484	0.0108
DMF LYMPHO vs. IFN	0.0027	0.0002
DMF LYMPHO vs. NATALIZUMAB	0.0004	<0.0001
FINGOLIMOD vs. IFN	0.0150	0.0021
FINGOLIMOD vs. NATALIZUMAB	0.0020	0.0001
NATALIZUMAB vs. TERFLUONOMIDE	0.0250	0.0042

CD8+ T CELLS (N, ml)	q-value	p-value
HD VS DMF LYMPHOPENIC	0.0058	0.0005
CLADRIBINE VS NATALIZUMAB	0.0309	0.0069
DMF VS NATALIZUMAB	0.0143	0.0028
DMF LYMPHOPENIC VS IFN	0.0100	0.0014
DMF LYMPHOPENIC VS NATALIZUMAB	0.0002	<0.0001
DMF LYMPHOPENIC VS TERIFLUNOMIDE	0.0142	0.0024
DMF LYMPHOPENIC VS RITUXIMAB/OCRELIZUMAB	0.0088	0.0010
FINGOLIMOD VS NATALIZUMAB	0.0031	0.0002

Supplementary Figure 6A Detailed statistical analysis of CD8+ clusters obtained using FlowSOM. The left side features dot plots illustrating the cell percentage within distinct clusters across various patient treatment groups and healthy donors (HD). On the right side, dot plots represent the absolute cell counts within these clusters for different patient treatment groups and HD. Scatter plots show individual values, the central bar represents the mean ± SD. Kruskal–Wallis test (one-sided) with Benjamini–Hochberg correction for multiple comparisons. Tables display statistically significant q-value and individual p-value obtained. For all graphs: HD: healthy donors (N=13); Cladribine (N=6) DMF: Dimethyl Fumarate (N=14); DMF Lymphopenic: Dimethyl Fumarate Lymphopenic (N=9); Fingolimod (N=12); IFN: Interferon 1b (N=13); Natalizumab (N=15); Teriflunomide (N=8); Rituximab/Ocrelizumab (N=11).

EMRA Tc_{17} $PD1^{+}$ $CD57^{+}$

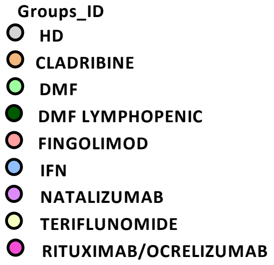

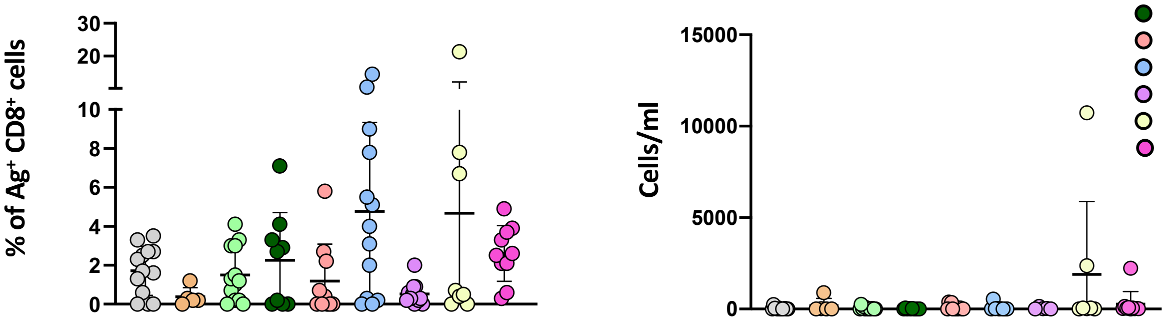

EM Tc_{0-2} $PD1^{+}$ $CD57^{+}$

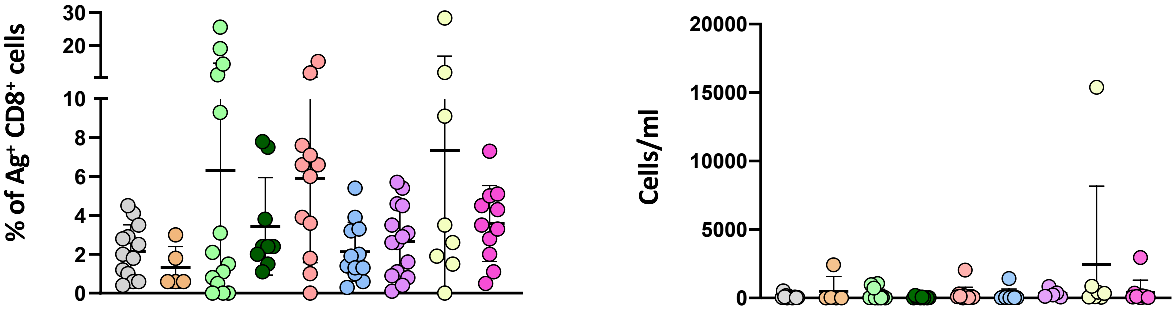

EMRA Tc_{17} $PD1^{-}$ $CD57^{+}$

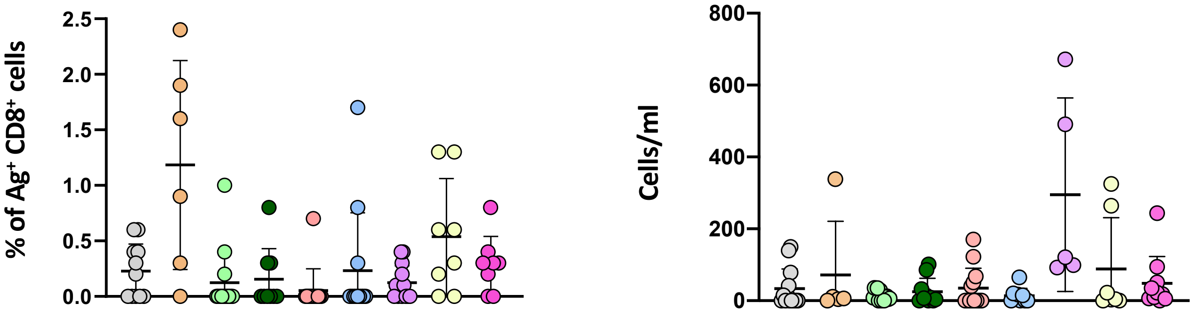

Supplementary Figure 6B. Detailed statistical analysis of CD8+ clusters obtained using FlowSOM. The left side features dot plots illustrating the cell percentage within distinct clusters across various patient treatment groups and healthy donors (HD). On the right side, dot plots represent the absolute cell counts within these clusters for different patient treatment groups and HD. Scatter plots show individual values, the central bar represents the mean ± SD. Kruskal–Wallis test (one-sided) with Benjamini–Hochberg correction for multiple comparisons. Tables display statistically significant q-value and individual p-value obtained. For all graphs: HD: healthy donors (N=13); Cladribine (N=6) DMF: Dimethyl Fumarate (N=14); DMF Lymphopenic: Dimethyl Fumarate Lymphopenic (N=9); Fingolimod (N=12); IFN: Interferon 1b (N=13); Natalizumab (N=15); Teriflunomide (N=8); Rituximab/Ocrelizumab (N=11).

EM $Tc_{0\text{-}2}PD1^{-}$ $CD57^{-}$

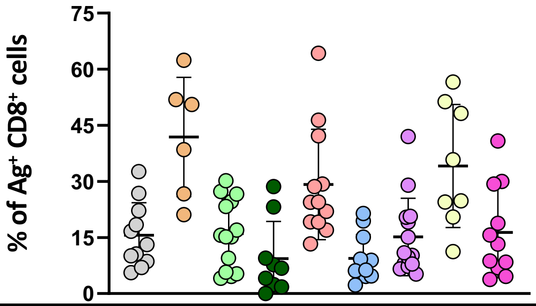

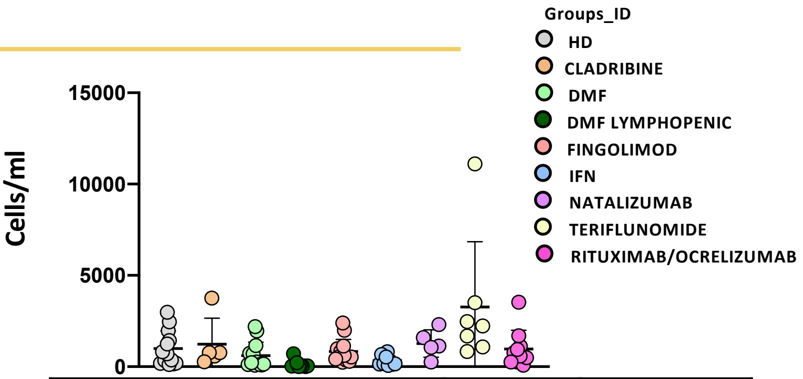

CD8$^+$ T CELLS (N, %)	q-value	p-value
HD vs. CLADRIBINE	0.0279	0.0078
CLADRIBINE vs. DMF	0.0212	0.0052
CLADRIBINE vs. DMF LYMPHO	0.0017	<0.0001
CLADRIBINE vs. IFN	0.0212	<0.0001
CLADRIBINE vs. NATALIZUMAB	0.0212	0.0028
CLADRIBINE vs. RITUXIMAB/OCRELIZUMAB	0.0212	0.0053
DMF vs. TERFLUONOMIDE	0.0420	0.0163
DMF LYMPHO vs. FINGOLIMOD	0.0024	0.0004
DMF LYMPHO vs. TERFLUONOMIDE	0.0021	0.0003
FINGOLIMOD vs. IFN	0.0021	0.0003
FINGOLIMOD vs. NATALIZUMAB	0.0420	0.0145
IFN vs. TERFLUONOMIDE	0.0021	0.0002
NATALIZUMAB vs. TERFLUONOMIDE	0.0294	0.0090
TERFLUONOMIDE vs. RITUXIMAB/OCRELIZUMAB	0.0420	0.0163

CD8$^+$ T CELLS (N, ml)	q-value	p-value
HD VS DMF LYMPHOPENIC	0.0029	0.0004
CLADRIBINE VS DMF LYMPHOPENIC	0.0083	0.0018
DMF VS TERIFLUNOMIDE	0.0049	0.0010
DMF LYMPHOPENIC VS FINGOLIMOD	0.0029	0.0002
DMF LYMPHOPENIC VS NATALIZUMAB	0.0029	0.0003
DMF LYMPHOPENIC VS TERIFLUNOMIDE	<0.0001	<0.0001
DMF LYMPHOPENIC VS RITUXIMAB/OCRELIZUMAB	0.0042	0.0007
IFN VS TERIFLUNOMIDE	0.0029	0.0004

TM Tc_1 $CXCR5^{+}$

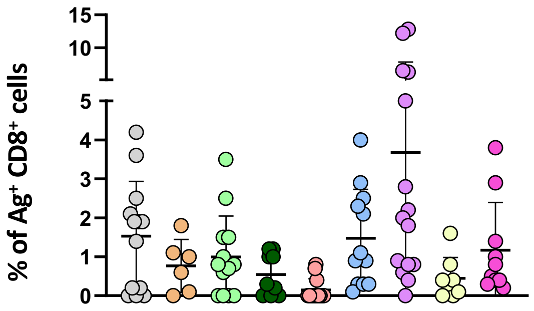

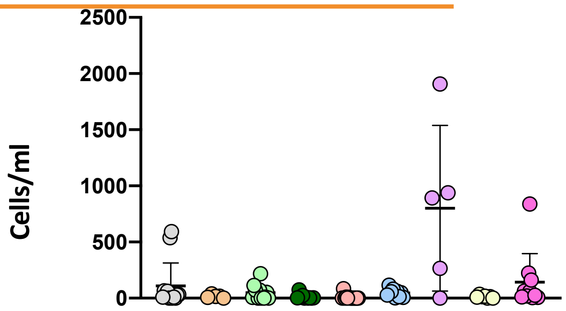

CD8+ T CELLS (N, %)	q-value	p-value
HD vs. FINGOLIMOD	0.0113	0.0009
FINGOLIMOD vs. IFN	0.0055	0.0003
FINGOLIMOD vs. NATALIZUMAB	<0.0001	<0.0001
FINGOLIMOD vs. RITUXIMAB/OCRELIZUMAB	0.0327	0.0036
NATALIZUMAB vs. TERFLUONOMIDE	0.0380	0.0053

CD8+ T CELLS (N, ml)	q-value	p-value
HD VS FINGOLIMOD	0.0318	0.0041
DMF LYMPHOPENIC VS NATALIZUMAB	0.0318	0.0044
FINGOLIMD VS IFN	0.0193	0.0016
FINGOLIMOD VS NATALIZUMAB	0.0072	0.0002
FINGOLIMOD VS RITUXIMAB/OCRELIZUMAB	0.0072	0.0004

Supplementary Figure 6C Detailed statistical analysis of CD8+ clusters obtained using FlowSOM. The left side features dot plots illustrating the cell percentage within distinct clusters across various patient treatment groups and healthy donors (HD). On the right side, dot plots represent the absolute cell counts within these clusters for different patient treatment groups and HD. Scatter plots show individual values, the central bar represents the mean ± SD. Kruskal–Wallis test (one-sided) with Benjamini–Hochberg correction for multiple comparisons. Tables display statistically significant q-value and individual p-value obtained. For all graphs: HD: healthy donors (N=13); Cladribine (N=6) DMF: Dimethyl Fumarate (N=14); DMF Lymphopenic: Dimethyl Fumarate Lymphopenic (N=9); Fingolimod (N=12); IFN: Interferon 1b (N=13); Natalizumab (N=15); Teriflunomide (N=8); Rituximab/Ocrelizumab (N=11).

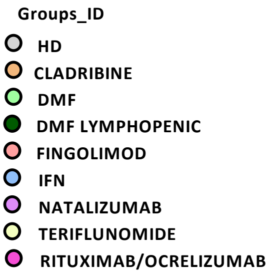

EM Tc$_1$ CXCR5$^+$

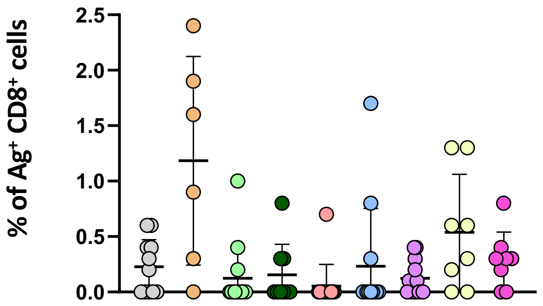

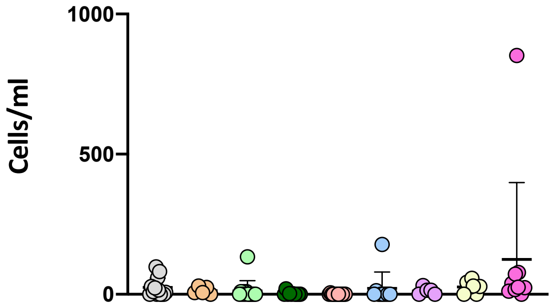

CD8$^+$ T CELLS (N, %)	q-value	p-value
CLADRIBINE vs. DMF	0.0279	0.0016
CLADRIBINE vs. FINGOLIMOD	0.0076	0.0002
CLADRIBINE vs. IFN	0.0329	0.0037
FINGOLIMOD vs. TERFLUONOMIDE	0.0288	0.0024

CD8$^+$ T CELLS (N, ml)	q-value	p-value
HD FINGOLIMD	0.0245	0.0041
DMF VS RITUXIMAB/OCRELIZUMAB	0.0167	0.0017
DMF LYMPHOPENIC VS RITUXIMAB/OCRELIZUMAB	0.0167	0.0012
FINGOLIMOD VS TERIFLUNOMIDE	0.0167	0.0019
FINGOLIMOD VS RITUXIMAB/OCRELIZUMAB	0.0012	<0.0001
IFN VS RITUXIMAB/OCRELIZUMAB	0.0193	0.0027

Supplementary Figure 6D Detailed statistical analysis of CD8+ clusters obtained using FlowSOM. The left side features dot plots illustrating the cell percentage within distinct clusters across various patient treatment groups and healthy donors (HD). On the right side, dot plots represent the absolute cell counts within these clusters for different patient treatment groups and HD. Scatter plots show individual values, the central bar represents the mean ± SD. Kruskal–Wallis test (one-sided) with Benjamini–Hochberg correction for multiple comparisons. Tables display statistically significant q-value and individual p-value obtained. For all graphs: HD: healthy donors (N=13); Cladribine (N=6) DMF: Dimethyl Fumarate (N=14); DMF Lymphopenic: Dimethyl Fumarate Lymphopenic (N=9); Fingolimod (N=12); IFN: Interferon 1b (N=13); Natalizumab (N=15); Teriflunomide (N=8); Rituximab/Ocrelizumab (N=11).

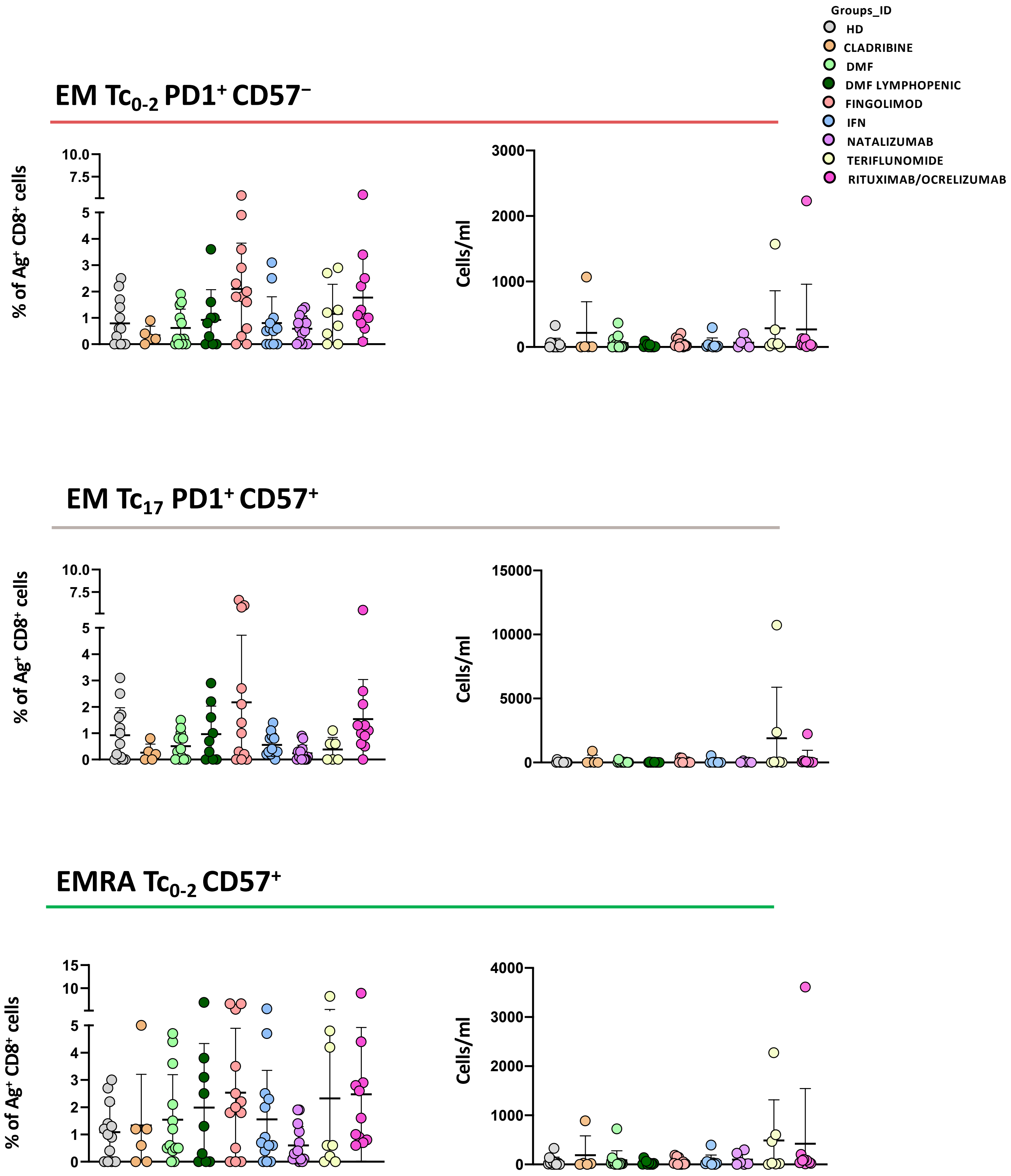

Supplementary Figure 6E Detailed statistical analysis of CD8+ clusters obtained using FlowSOM. The left side features dot plots illustrating the cell percentage within distinct clusters across various patient treatment groups and healthy donors (HD). On the right side, dot plots represent the absolute cell counts within these clusters for different patient treatment groups and HD. Scatter plots show individual values, the central bar represents the mean ± SD. Kruskal–Wallis test (one-sided) with Benjamini–Hochberg correction for multiple comparisons. Tables display statistically significant q-value and individual p-value obtained. For all graphs: HD: healthy donors (N=13); Cladribine (N=6) DMF: Dimethyl Fumarate (N=14); DMF Lymphopenic: Dimethyl Fumarate Lymphopenic (N=9); Fingolimod (N=12); IFN: Interferon 1b (N=13); Natalizumab (N=15); Teriflunomide (N=8); Rituximab/Ocrelizumab (N=11).

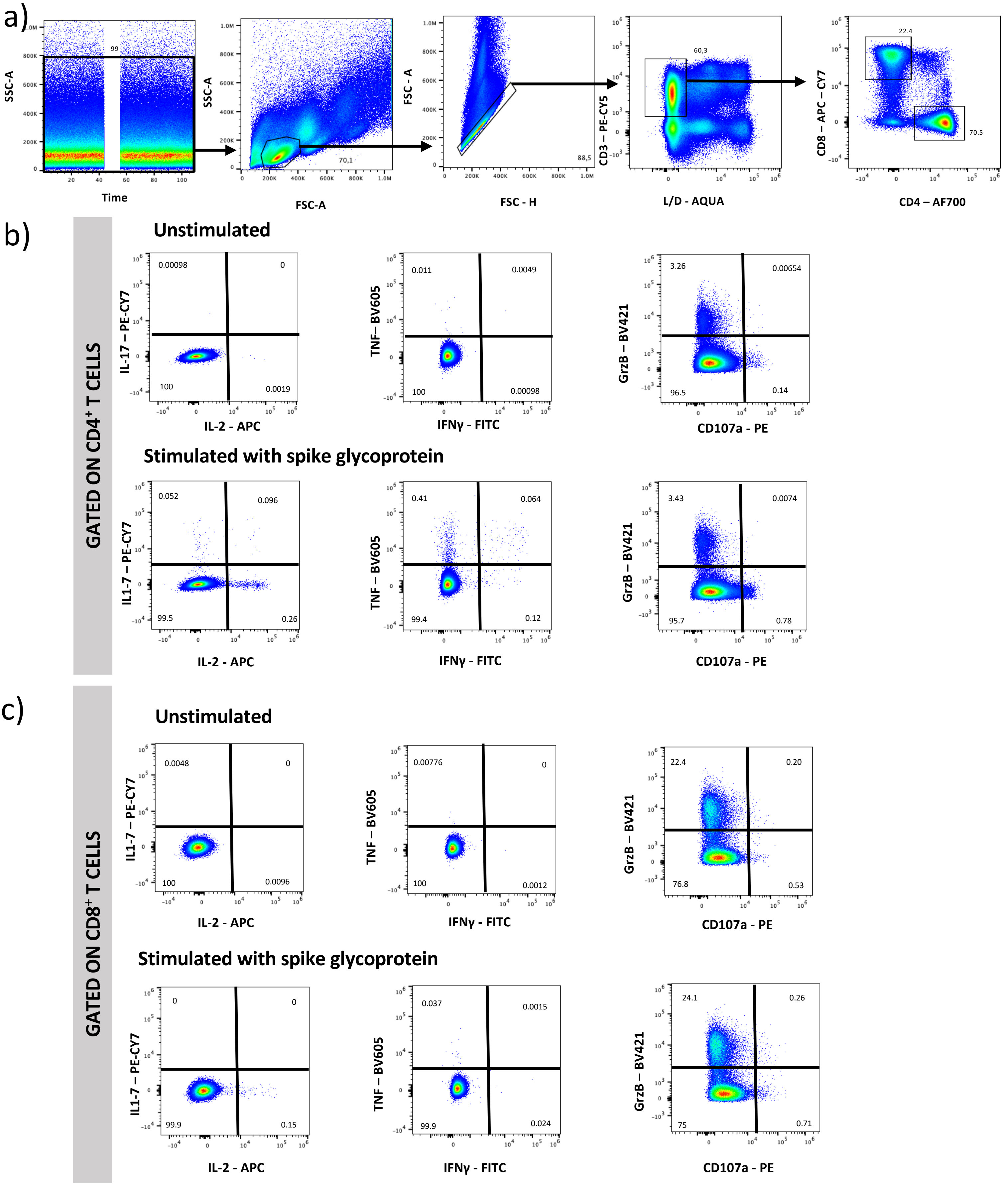

Supplementary Figure 7. Gating strategy and representative plots of intracellular staining analysis of cytokine producing cells (ICS) after overnight stimulation with spike protein compared to unstimulated control. Cytokine production and polyfunctionality of antigen-specific $CD4^+$ T cells (**panel b**) and $CD8^+$ T cells (**panel c**) . Numbers in the dot plots indicate the percentage of $CD4^+$ and $CD8^+$ cells identified by the gates. Comparison between the total production of IFN-γ, TNF, IL-17, IL-2, CD107a, and GZMB .

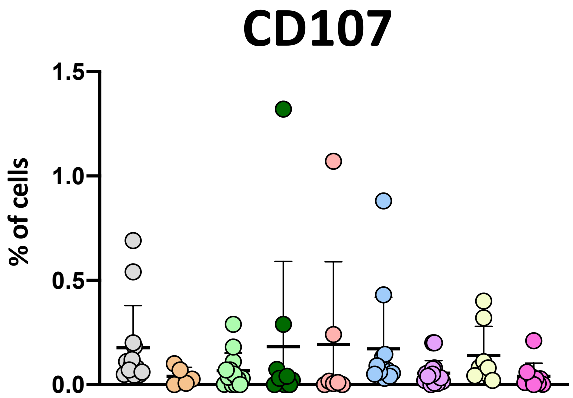

CD4⁺ T CELLS	q-value	P-value
HD VS CLADRBINA	0.0395	0.0296
HD VS DMF	0.0274	0.0137
HD VS DMF LYMPHOPENIC	0.0296	0.0185
HD VS FINGOLIMOD	0.0274	0.0123
HD VS NATALIZUMAB	0.0274	0.0098
HD VS RITUXIMAB/OCRELIZUMAB	0.0176	0.0022

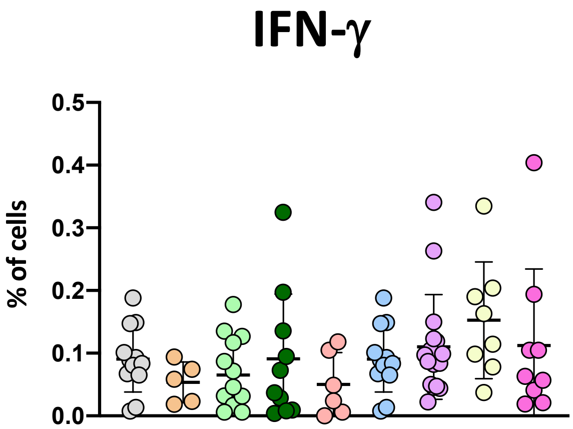

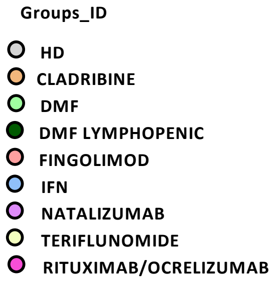

Supplementary Figure 8A. Detailed statistical analysis of CD4⁺ total cytokine production. Dot plots show the percentage of cells in different treated groups of patients and healthy donors (HC). Scatter plots show individual values, the central bar represents the mean ± SD. Kruskal–Wallis test (one-sided) with Benjamini–Hochberg correction for multiple comparisons. Tables display statistically significant q-value and individual p-value obtained. For all graphs: HD: healthy donors (N=13); Cladribine (N=6) DMF: Dimethyl Fumarate (N=14); DMF Lymphopenic: Dimethyl Fumarate Lymphopenic (N=9); Fingolimod (N=12); IFN: Interferon 1β (N=13); Natalizumab (N=15); Teriflunomide (N=8); Rituximab/Ocrelizumab (N=11).

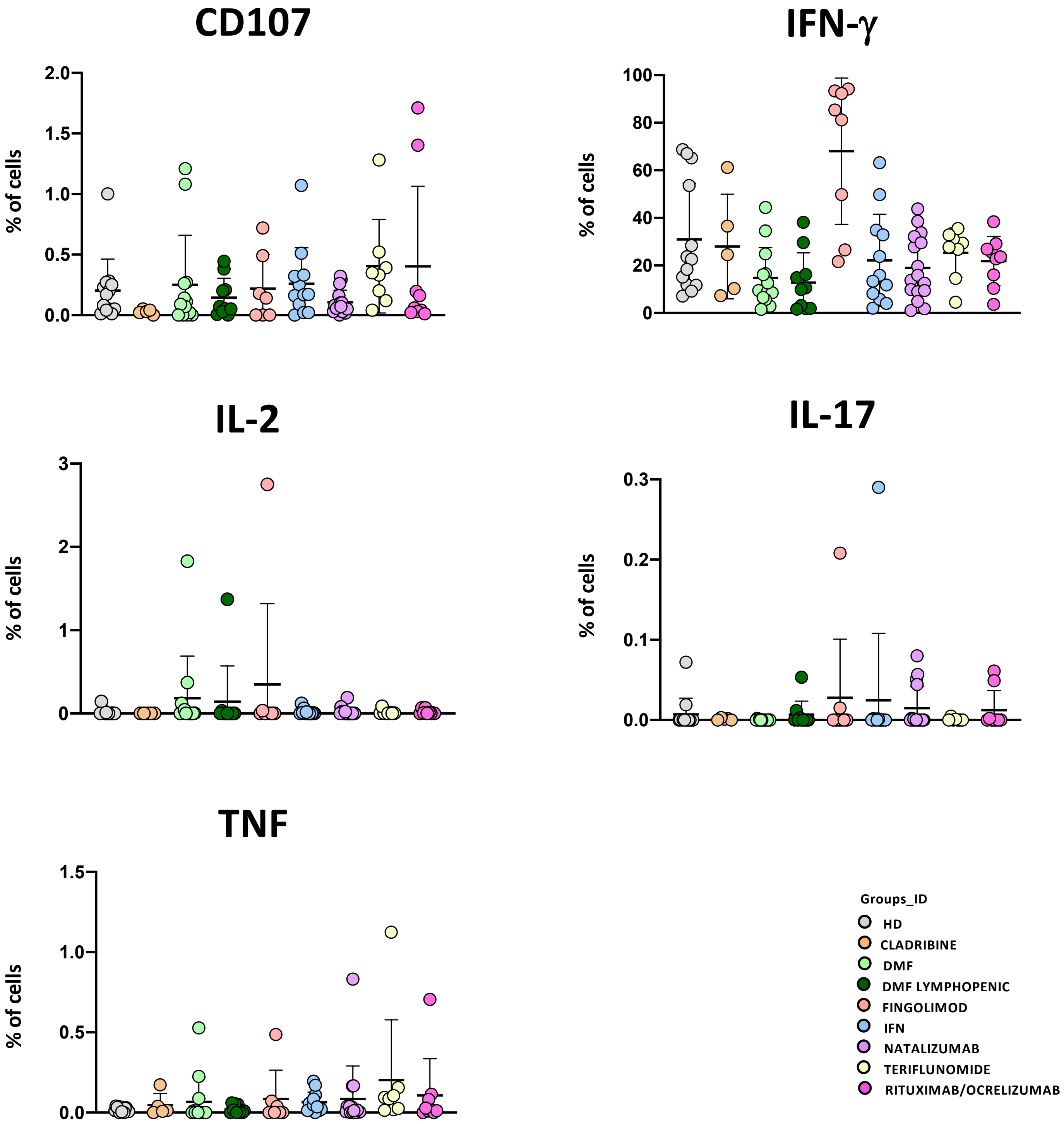

Supplementary Figure 8B. Detailed statistical analysis of CD8+ total cytokine production. Dot plots show the percentage of cells in different treated groups of patients and healthy donors (HD). Scatter plots show individual values, the central bar represents the mean ± SD. Kruskal–Wallis test (one-sided) with Benjamini–Hochberg correction for multiple comparisons. Tables display statistically significant q-value and individual p-value obtained. For all graphs: HD: healthy donors (N=13); Cladribine (N=6) DMF: Dimethyl Fumarate (N=14); DMF Lymphopenic: Dimethyl Fumarate Lymphopenic (N=9); Fingolimod (N=12); IFN: Interferon 1b (N=13); Natalizumab (N=15); Teriflunomide (N=8); Rituximab/Ocrelizumab (N=11).

CD107^{+} IFN-γ^{-} IL2^{-} IL17^{-} TNF^{-}

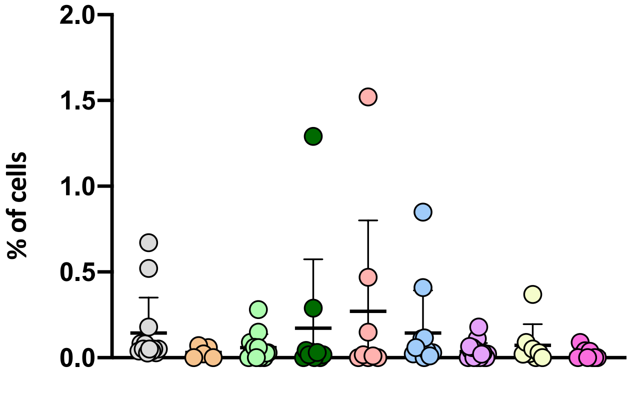

CD107^{-} IFN-γ^{-} IL2^{+} IL17^{-} TNF^{-}

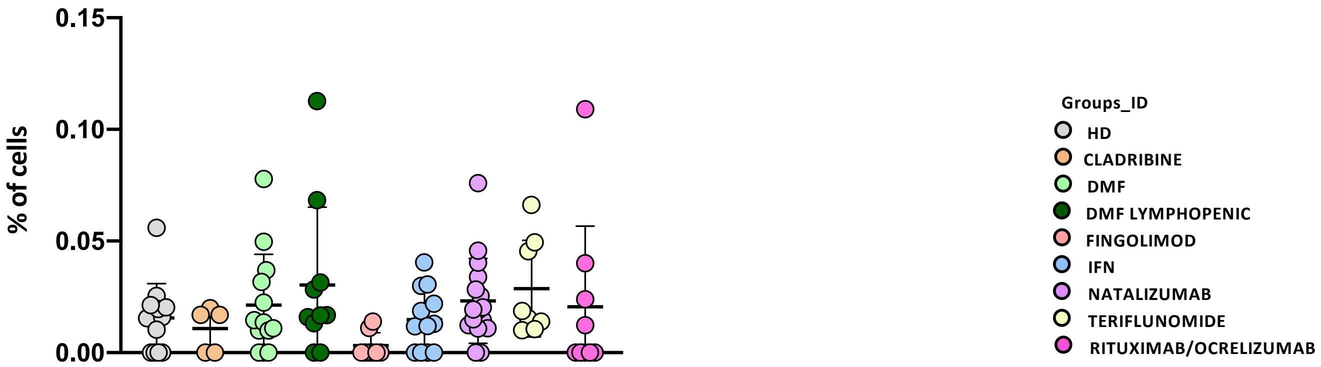

Supplementary Figure 9. Detailed statistical analysis of CD4+ polyfunctionality. Dot plots show the percentage of cells in different treated groups of patients and healthy donors (HD). Scatter plots show individual values, the central bar represents the mean ± SD. Kruskal–Wallis test (one-sided) with Benjamini–Hochberg correction for multiple comparisons. Tables display statistically significant q-value and individual p-value obtained. For all graphs: HD: healthy donors (N=13); Cladribine (N=6) DMF: Dimethyl Fumarate (N=14); DMF Lymphopenic: Dimethyl Fumarate Lymphopenic (N=9); Fingolimod (N=12); IFN: Interferon 1b (N=13); Natalizumab (N=15); Teriflunomide (N=8); Rituximab/Ocrelizumab (N=11).

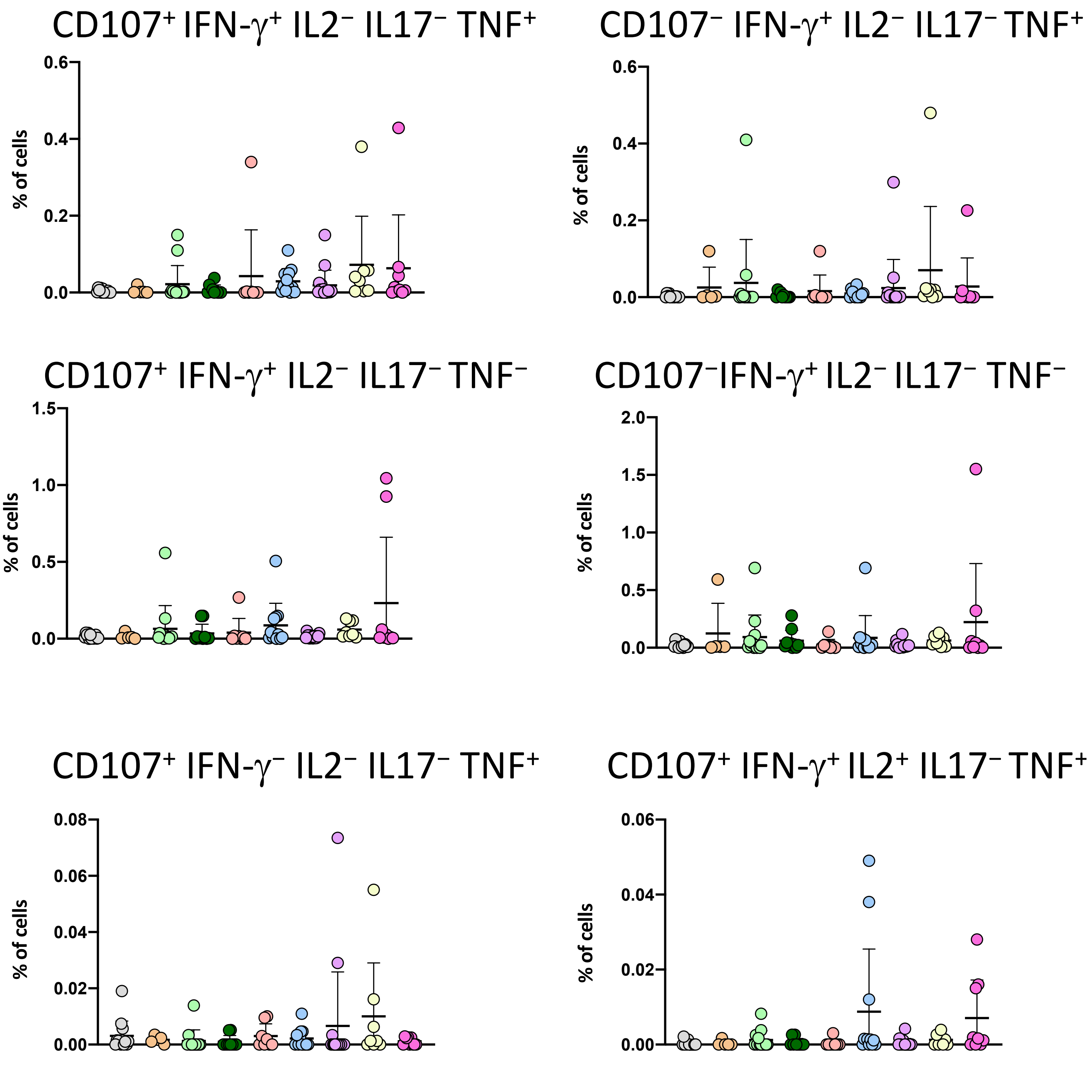

Supplementary Figure 10. Detailed statistical analysis of CD8+ polyfunctionality. Dot plots show the percentage of cells in different treated groups of patients and healthy donors (HD). Scatter plots show individual values, the central bar represents the mean ± SD. Kruskal–Wallis test (one-sided) with Benjamini–Hochberg correction for multiple comparisons. Tables display statistically significant q-value and individual p-value obtained. For all graphs: HD: healthy donors (N=13); Cladribine (N=6) DMF: Dimethyl Fumarate (N=14); DMF Lymphopenic: Dimethyl Fumarate Lymphopenic (N=9); Fingolimod (N=12); IFN: Interferon 1b (N=13); Natalizumab (N=15); Teriflunomide (N=8); Rituximab/Ocrelizumab (N=11).

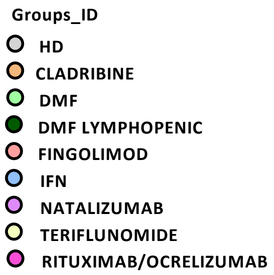

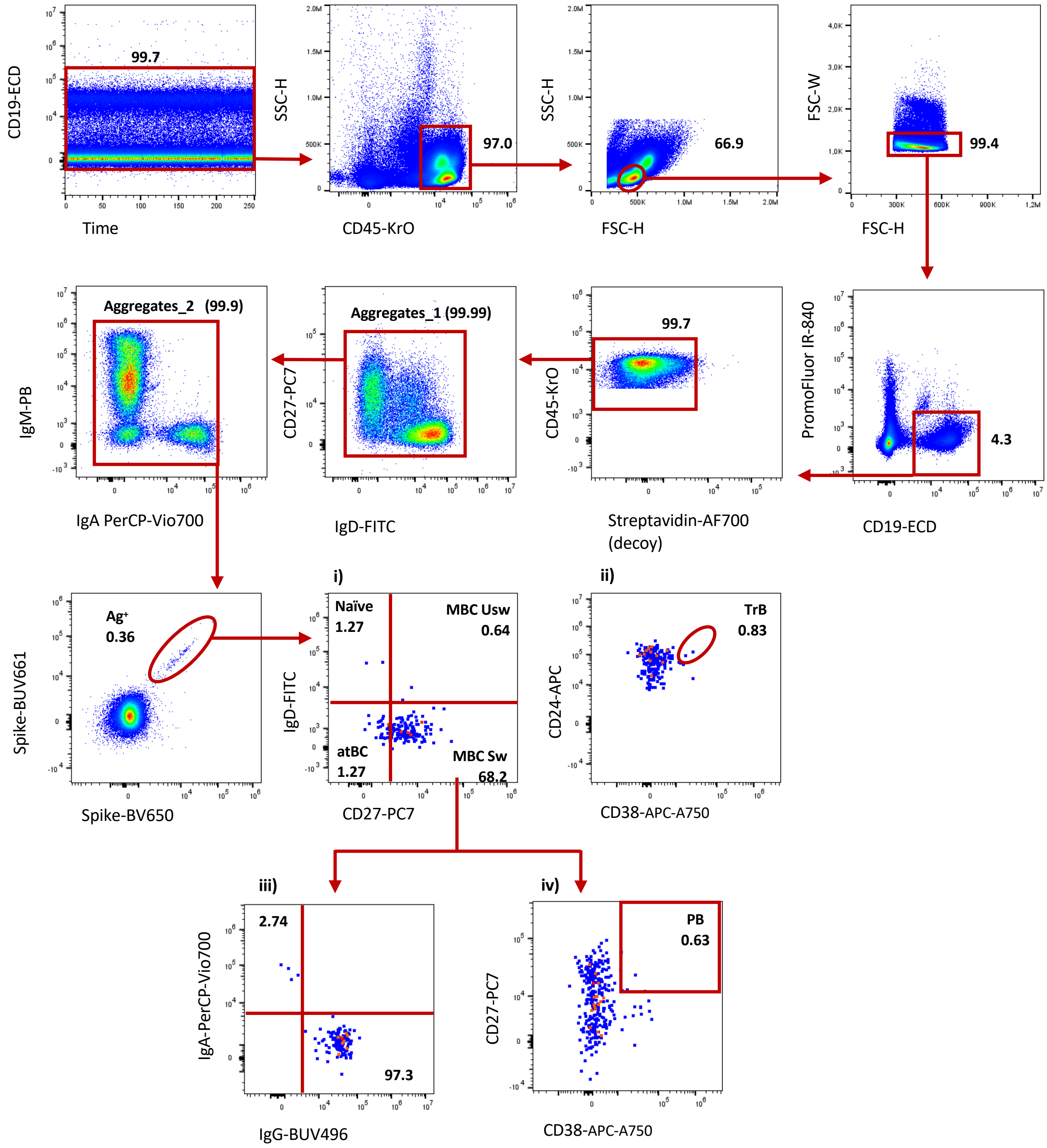

Supplementary Figure 11. Gating strategy before computational analysis of Ag⁺ B cells. The Time *vs* CD19-ECD gate was used to exclude unstable flow during acquisition. Leukocytes were selected as CD45⁺ cells. Next, lymphocytes were selected based on their physical characteristics, and doublets were excluded from the analysis by utilizing forward scatter height (FSC-H) and forward scatter width (FSC-W) parameters. Living B cells were selected as PromoFluor⁻ and CD19⁺ . Aggregates were removed and Ag⁺ (Spike-BUV661⁺ and Spike-BV650⁺) and Ag⁻ ((Spike-BUV661⁻ and Spike-BV650⁻) B cells were displayed. Inside Ag⁺ B cells. we identified **i)** Naïve cells. Memory Unswitched B cells (MBC Usw). Memory Switched B cells (MBC Sw). and atypical B cells (atBC); **ii)** transitional B cells (TrB). Within MBC Sw gate. **iii)** the quadrant plots reported the different percentages of IgA⁺. IgG⁺ cells while **iv)** the dot plots plasmablast (PB) percentage.

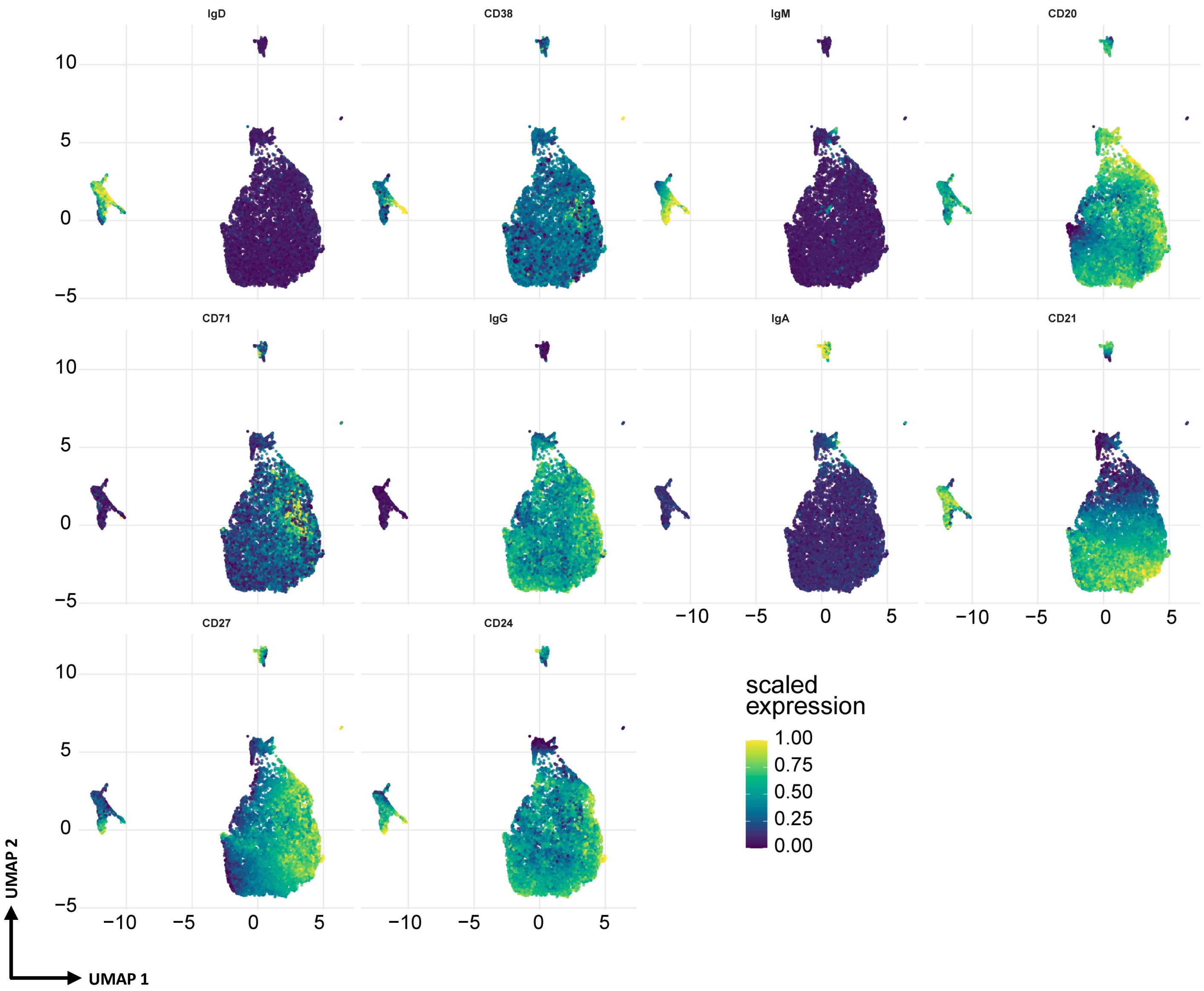

Supplementary Figure 12. Uniform Manifold Approximation and Projection (UMAP) plot shows the 2D spatial distribution of B cells from 28 healthy donors vaccinated against Sars-CoV2 and 106 patients with multiple sclerosis undergoing different disease-modifying therapies (DMT) and vaccinated against COVID-19. UMAP graphs colored by the expression of 10 markers used for $CD19^+$ antigen specific B cell phenotyping. Blue represents lower expression while yellow represent higher expression.

Naive

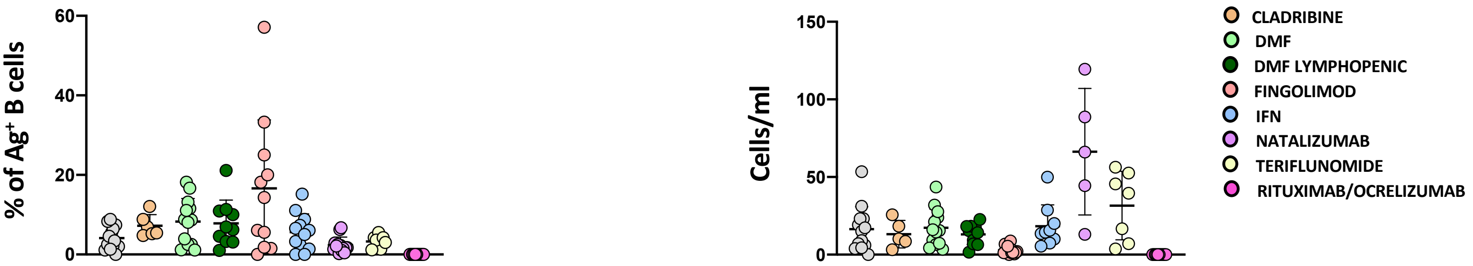

Naive (N, %)	q value	Individual P Value
HD vs. RITUXIMAB/OCRELIZUMAB	0.0043	0.0007
CLADRIBINE vs. RITUXIMAB/OCRELIZUMAB	0.0003	<0.0001
DMF vs. NATALIZUMAB	0.0367	0.0082
DMF vs. RITUXIMAB/OCRELIZUMAB	<0.0001	<0.0001
DMF LYMPHO vs. RITUXIMAB/OCRELIZUMAB	<0.0001	<0.0001
FINGOLIMOD vs. NATALIZUMAB	0.0186	0.0036
FINGOLIMOD vs. RITUXIMAB/OCRELIZUMAB	<0.0001	<0.0001
IFN vs. RITUXIMAB/OCRELIZUMAB	0.001	0.0001
TERFLUNOMIDE vs. RITUXIMAB/OCRELIZUMAB	0.0411	0.0103

Naive (N, mL)	q value	Individual P Value
HD vs. FINGOLIMOD	0.018	0.006
HD vs. RITUXIMAB/OCRELIZUMAB	0.0003	<0.0001
CLADRIBINE vs. RITUXIMAB/OCRELIZUMAB	0.0077	0.0021
DMF vs. FINGOLIMOD	0.0074	0.0018
DMF vs. RITUXIMAB/OCRELIZUMAB	<0.0001	<0.0001
DMF LYMPHO vs. FINGOLIMOD	0.0368	0.0133
DMF LYMPHO vs. RITUXIMAB/OCRELIZUMAB	0.0008	0.0002
FINGOLIMOD vs. IFN	0.0106	0.0033
FINGOLIMOD vs. NATALIZUMAB	0.0003	<0.0001
FINGOLIMOD vs. TERFLUNOMIDE	0.0028	0.0006
IFN vs. RITUXIMAB/OCRELIZUMAB	0.0002	<0.0001
NATALIZUMAB vs. RITUXIMAB/OCRELIZUMAB	<0.0001	<0.0001
TERFLUNOMIDE vs. RITUXIMAB/OCRELIZUMAB	<0.0001	<0.0001

Immature TrB

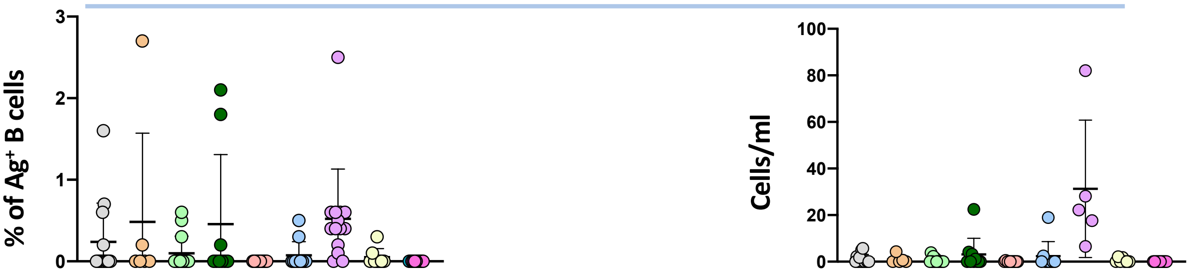

Im TrB (N, %)	q value	Individual P Value
HD vs. NATALIZUMAB	0.0203	0.0034
DMF vs. NATALIZUMAB	0.0024	0.0003
FINGOLIMOD vs. NATALIZUMAB	0.0002	<0.0001
IFN vs. NATALIZUMAB	0.0024	0.0003
NATALIZUMAB vs. TERFLUNOMIDE	0.0109	0.0015
NATALIZUMAB vs. RITUXIMAB/OCRELIZUMAB	0.0002	<0.0001

Im TrB (N mL)	q value	Individual P Value
HD vs. NATALIZUMAB	0.0016	0.0002
CLADRIBINE vs. NATALIZUMAB	0.0184	0.0041
DMF vs. NATALIZUMAB	0.0005	<0.0001
DMF LYMPHO vs. NATALIZUMAB	0.008	0.0016
FINGOLIMOD vs. NATALIZUMAB	<0.0001	<0.0001
IFN vs. NATALIZUMAB	0.0016	0.0002
NATALIZUMAB vs. TERFLUNOMIDE	0.0024	0.0004
NATALIZUMAB vs. RITUXIMAB/OCRELIZUMAB	<0.0001	<0.0001

Supplementary Figure 13A. Detailed statistical analysis of 11 clusters obtained using FlowSOM. On the left, dot plots show the percentage of cells in different treated groups of patients and healthy donors (HD). On the right, dot plots show the absolute number of cells in different treated groups of patients and healthy donors (HD). Scatter plots show individual values, mean ± standard deviation (SD). Tables display statistically significant q-value and individual p-value obtained using Kruskal–Wallis test with Benjamini–Hochberg correction for multiple comparisons. Abbreviations: Naïve; TrB. transitional B cells; MBC Usw. memory B cell unswitched; MBC. memory B cell; PB. plasmablasts; atBC. atypical B cell. HD, healthy donors n=13; Cladribine n=6; DMF, Dimethyl Fumarate n=14; DMF Lympho, Dimethyl Fumarate Lymphopenic n=9; Fingolimod n=12; IFN, interferon n=13; Natalizumab n=15; Teriflunomide n=8; rituximab/ocrelizumab (Ocrelizumab;/Rituximab) n=11.

TrB

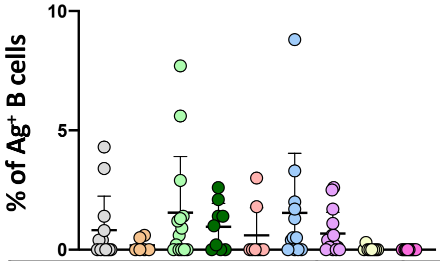

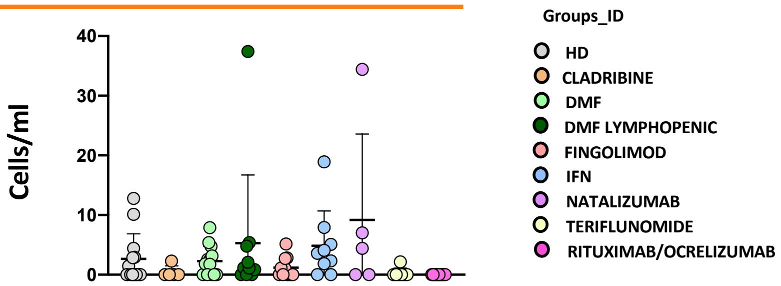

TrB (N, %)	q value	Individual P Value
DMF vs. RITUXIMAB/OCRELIZUMAB	0.0269	0.0013
DMF LYMPHO vs. RITUXIMAB/OCRELIZUMAB	0.0344	0.0038
IFN vs. RITUXIMAB/OCRELIZUMAB	0.0269	0.0015
NATALIZUMAB vs. RITUXIMAB/OCRELIZUMAB	0.0344	0.0029

TrB (N, mL)	q value	Individual P Value
IFN vs. RITUXIMAB/OCRELIZUMAB	0.0204	0.0006

MBC Usw

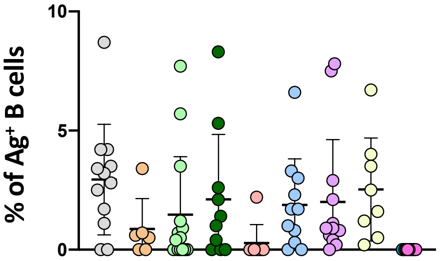

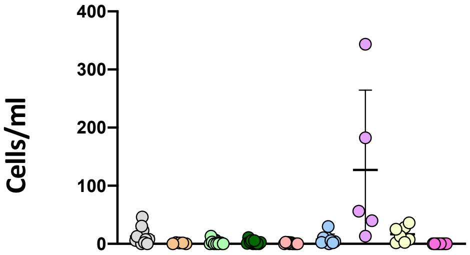

MBC Usw (N,%)	q value	Individual P Value
HD vs. FINGOLIMOD	0.0047	0.0005
HD vs. RITUXIMAB/OCRELIZUMAB	0.0004	<0.0001
DMF LYMPHO vs. RITUXIMAB/OCRELIZUMAB	0.0141	0.0027
FINGOLIMOD vs. IFN	0.0405	0.0101
FINGOLIMOD vs. NATALIZUMAB	0.0376	0.0083
FINGOLIMOD vs. TERFLUNOMIDE	0.0114	0.0019
IFN vs. RITUXIMAB/OCRELIZUMAB	0.0057	0.0008
NATALIZUMAB vs. RITUXIMAB/OCRELIZUMAB	0.0047	0.0005
TERFLUNOMIDE vs. RITUXIMAB/OCRELIZUMAB	0.0021	0.0001

MBC Usw (N, mL)	q value	Individual P Value
HD vs. CLADRIBINE	0.0478	0.0239
HD vs. DMF	0.0288	0.0108
HD vs. FINGOLIMOD	0.0039	0.001
HD vs. RITUXIMAB/OCRELIZUMAB	0.0002	<0.0001
CLADRIBINE vs. NATALIZUMAB	0.0039	0.0009
CLADRIBINE vs. TERFLUNOMIDE	0.0352	0.0128
DMF vs. NATALIZUMAB	0.0021	0.0003
DMF vs. TERFLUNOMIDE	0.0223	0.0068
DMF LYMPHO vs. NATALIZUMAB	0.0059	0.0016
FINGOLIMOD vs. IFN	0.0352	0.0137
FINGOLIMOD vs. NATALIZUMAB	0.0003	<0.0001
FINGOLIMOD vs. TERFLUNOMIDE	0.0039	0.0008
IFN vs. RITUXIMAB/OCRELIZUMAB	0.0039	0.0008
NATALIZUMAB vs. RITUXIMAB/OCRELIZUMAB	<0.0001	<0.0001
TERFLUNOMIDE vs. RITUXIMAB/OCRELIZUMAB	0.0003	<0.0001

Supplementary Figure 13B. Detailed statistical analysis of 11 clusters obtained using FlowSOM. On the left, dot plots show the percentage of cells in different treated groups of patients and healthy donors (HD). On the right, dot plots show the absolute number of cells in different treated groups of patients and healthy donors (HD). Scatter plots show individual values, mean ± standard deviation (SD). Tables display statistically significant q-value and individual p-value obtained using Kruskal–Wallis test with Benjamini–Hochberg correction for multiple comparisons. Abbreviations: Naïve; TrB. transitional B cells; MBC Usw. memory B cell unswitched; MBC. memory B cell; PB. plasmablasts; atBC. atypical B cell. HD, healthy donors n=13; Cladribine n=6; DMF, Dimethyl Fumarate n=14; DMF Lympho, Dimethyl Fumarate Lymphopenic n=9; Fingolimod n=12; IFN, interferon n=13; Natalizumab n=15; Teriflunomide n=8; rituximab/ocrelizumab (Ocrelizumab;/Rituximab) n=11.

IgG CD21^{low}

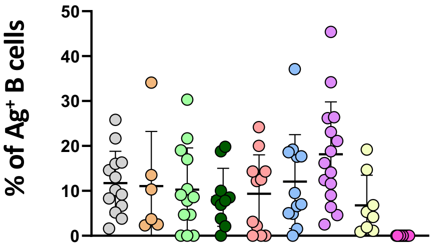

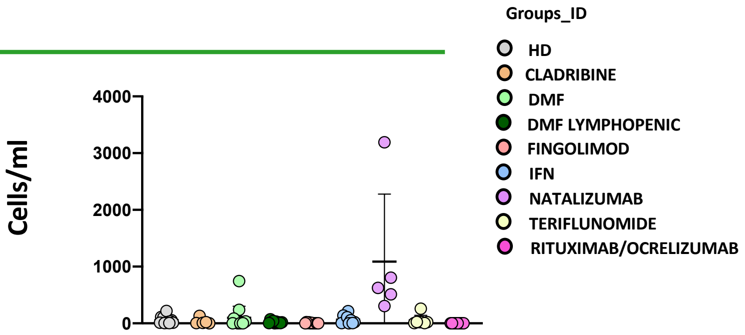

MBC IgG CD21 low (N, %)	q value	Individual P Value
HD vs. RITUXIMAB/OCRELIZUMAB	0.0005	<0.0001
CLADRIBINE vs. RITUXIMAB/OCRELIZUMAB	0.0216	0.0042
DMF vs. RITUXIMAB/OCRELIZUMAB	0.0043	0.0005
DMF LYMPHO vs. RITUXIMAB/OCRELIZUMAB	0.0117	0.0019
FINGOLIMOD vs. RITUXIMAB/OCRELIZUMAB	0.0117	0.0018
IFN vs. RITUXIMAB/OCRELIZUMAB	0.0014	0.0001
NATALIZUMAB vs. RITUXIMAB/OCRELIZUMAB	<0.0001	<0.0001

MBC IgG CD21 low (N, mL)	q value	Individual P Value
HD vs. CLADRIBINE	0.0478	0.0239
HD vs. DMF	0.0288	0.0108
HD vs. FINGOLIMOD	0.0039	0.001
HD vs. RITUXIMAB/OCRELIZUMAB	0.0002	<0.0001
CLADRIBINE vs. NATALIZUMAB	0.0306	0.0102
CLADRIBINE vs. RITUXIMAB/OCRELIZUMAB	0.038	0.0137
DMF vs. NATALIZUMAB	0.0237	0.0066
DMF vs. RITUXIMAB/OCRELIZUMAB	0.0017	0.0002
DMF LYMPHO vs. NATALIZUMAB	0.0136	0.0034
DMF LYMPHO vs. RITUXIMAB/OCRELIZUMAB	0.0098	0.0022
FINGOLIMOD vs. IFN	0.0272	0.0083
FINGOLIMOD vs. NATALIZUMAB	0.0001	<0.0001
IFN vs. RITUXIMAB/OCRELIZUMAB	0.0008	<0.0001
NATALIZUMAB vs. RITUXIMAB/OCRELIZUMAB	<0.0001	<0.0001
TERFLUNOMIDE vs. RITUXIMAB/OCRELIZUMAB	0.0056	0.0009

MBC IgG CD20^{-}

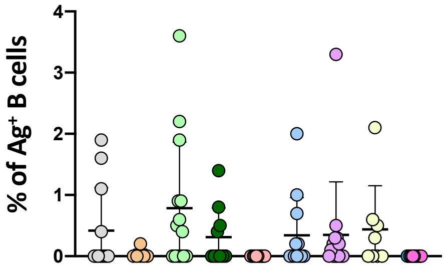

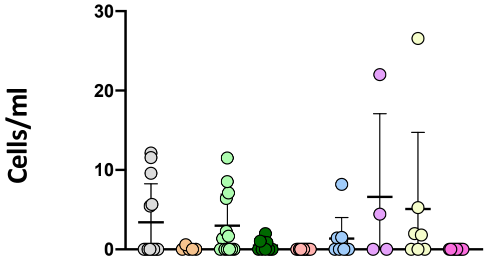

MBC IgG CD20^{-} (N, %)	q value	Individual P Value
DMF vs. FINGOLIMOD	0.0237	0.0013
DMF vs. RITUXIMAB/OCRELIZUMAB	0.0237	0.0013

Supplementary Figure 13C. Detailed statistical analysis of 11 clusters obtained using FlowSOM. On the left, dot plots show the percentage of cells in different treated groups of patients and healthy donors (HD). On the right, dot plots show the absolute number of cells in different treated groups of patients and healthy donors (HD). Scatter plots show individual values, mean ± standard deviation (SD). Tables display statistically significant q-value and individual p-value obtained using Kruskal–Wallis test with Benjamini–Hochberg correction for multiple comparisons. Abbreviations: Naïve; TrB. transitional B cells; MBC Usw. memory B cell unswitched; MBC. memory B cell; PB. plasmablasts; atBC. atypical B cell. HD, healthy donors n=13; Cladribine n=6; DMF, Dimethyl Fumarate n=14; DMF Lympho, Dimethyl Fumarate Lymphopenic n=9; Fingolimod n=12; IFN, interferon n=13; Natalizumab n=15; Teriflunomide n=8; rituximab/ocrelizumab (Ocrelizumab;/Rituximab) n=11.

MBC IgG CD71

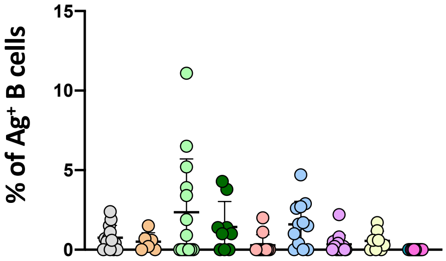

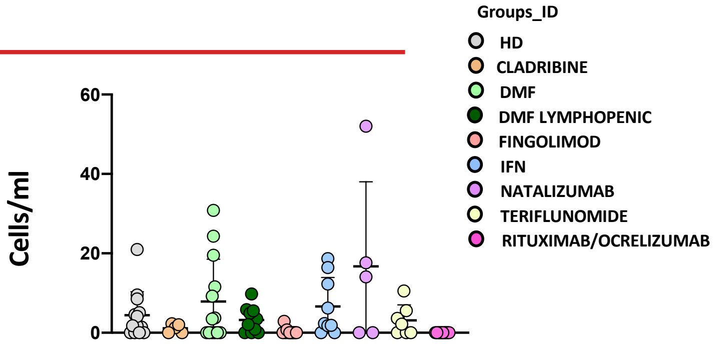

**MBC IgG CD71 (N, %)**	q value	Individual P Value
HD vs. RITUXIMAB/OCRELIZUMAB	0.029	0.004
DMF vs. RITUXIMAB/OCRELIZUMAB	0.029	0.0024
DMF LYMPHO vs. RITUXIMAB/OCRELIZUMAB	0.029	0.0021
FINGOLIMOD vs. IFN	0.029	0.0036
IFN vs. RITUXIMAB/OCRELIZUMAB	0.0051	0.0001

**MBC IgG CD71 (N, mL)**	q value	Individual P Value
HD vs. FINGOLIMOD	0.0078	0.002
HD vs. RITUXIMAB/OCRELIZUMAB	<0.0001	<0.0001
DMF vs. RITUXIMAB/OCRELIZUMAB	0.0394	0.0044
DMF LYMPHO vs. RITUXIMAB/OCRELIZUMAB	0.0496	0.0083
FINGOLIMOD vs. IFN	0.0394	0.0055
IFN vs. RITUXIMAB/OCRELIZUMAB	0.0394	0.0014
NATALIZUMAB vs. RITUXIMAB/OCRELIZUMAB	0.0394	0.0055

MBC IgG

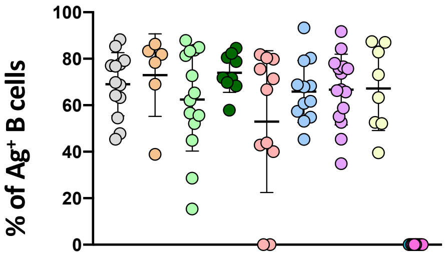

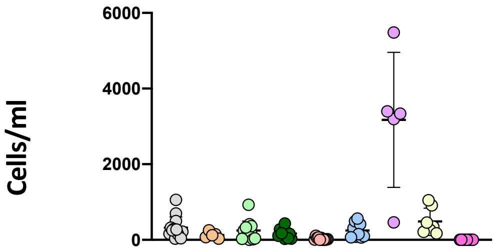

**MBC IgG (N, %)**	q value	Individual P Value
HD vs. RITUXIMAB/OCRELIZUMAB	0.0002	<0.0001
CLADRIBINE vs. RITUXIMAB/OCRELIZUMAB	0.0003	<0.0001
DMF vs. RITUXIMAB/OCRELIZUMAB	0.0005	<0.0001
DMF LYMPHO vs. RITUXIMAB/OCRELIZUMAB	0.0002	<0.0001
FINGOLIMOD vs. RITUXIMAB/OCRELIZUMAB	0.0108	0.0024
IFN vs. RITUXIMAB/OCRELIZUMAB	0.0011	0.0002
NATALIZUMAB vs. RITUXIMAB/OCRELIZUMAB	0.0003	<0.0001
TERFLUNOMIDE vs. RITUXIMAB/OCRELIZUMAB	0.0011	0.0002

**MBC IgG (N, mL)**	q value	Individual P Value
HD vs. FINGOLIMOD	0.003	0.0007
HD vs. RITUXIMAB/OCRELIZUMAB	<0.0001	<0.0001
CLADRIBINE vs. NATALIZUMAB	0.0196	0.0065
CLADRIBINE vs. RITUXIMAB/OCRELIZUMAB	0.0405	0.0169
DMF vs. FINGOLIMOD	0.0205	0.0074
DMF vs. NATALIZUMAB	0.0227	0.0088
DMF vs. RITUXIMAB/OCRELIZUMAB	0.0005	<0.0001
DMF LYMPHO vs. NATALIZUMAB	0.018	0.0055
DMF LYMPHO vs. RITUXIMAB/OCRELIZUMAB	0.003	0.0007
FINGOLIMOD vs. IFN	0.018	0.0051
FINGOLIMOD vs. NATALIZUMAB	<0.0001	<0.0001
FINGOLIMOD vs. TERFLUNOMIDE	0.001	0.0002
IFN vs. RITUXIMAB/OCRELIZUMAB	0.0005	<0.0001
NATALIZUMAB vs. RITUXIMAB/OCRELIZUMAB	<0.0001	<0.0001
TERFLUNOMIDE vs. RITUXIMAB/OCRELIZUMAB	<0.0001	<0.0001

Supplementary Figure 13D. Detailed statistical analysis of 11 clusters obtained using FlowSOM. On the left, dot plots show the percentage of cells in different treated groups of patients and healthy donors (HD). On the right, dot plots show the absolute number of cells in different treated groups of patients and healthy donors (HD). Scatter plots show individual values, mean ± standard deviation (SD). Tables display statistically significant q-value and individual p-value obtained using Kruskal–Wallis test with Benjamini–Hochberg correction for multiple comparisons. Abbreviations: Naïve; TrB. transitional B cells; MBC Usw. memory B cell unswitched; MBC. memory B cell; PB. plasmablasts; atBC. atypical B cell. HD, healthy donors n=13; Cladribine n=6; DMF, Dimethyl Fumarate n=14; DMF Lympho, Dimethyl Fumarate Lymphopenic n=9; Fingolimod n=12; IFN, interferon n=13; Natalizumab n=15; Teriflunomide n=8; rituximab/ocrelizumab (Ocrelizumab;/Rituximab) n=11.

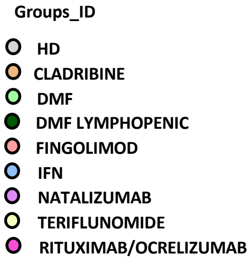

MBC IgA

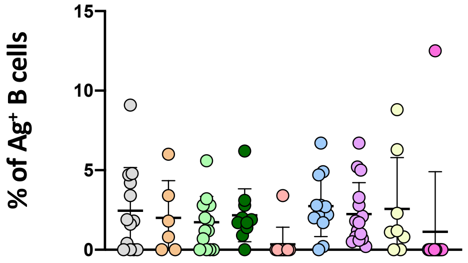

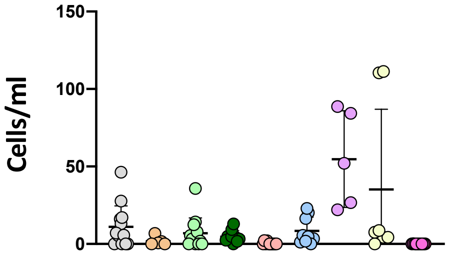

MBC IgA (N, %)	q value	Individual P Value
HD vs. FINGOLIMOD	0.0385	0.0083
HD vs. RITUXIMAB/OCRELIZUMAB	0.0385	0.0086
DMF LYMPHO vs. FINGOLIMOD	0.0294	0.0047
DMF LYMPHO vs. RITUXIMAB/OCRELIZUMAB	0.0294	0.0049
FINGOLIMOD vs. IFN	0.0079	0.0004
FINGOLIMOD vs. NATALIZUMAB	0.0161	0.0018
IFN vs. RITUXIMAB/OCRELIZUMAB	0.0079	0.0004
NATALIZUMAB vs. RITUXIMAB/OCRELIZUMAB	0.0161	0.0018

MBC IgA (N, mL)	q value	Individual P Value
HD vs. FINGOLIMOD	0.0069	0.0017
HD vs. RITUXIMAB/OCRELIZUMAB	0.0069	0.0009
CLADRIBINE vs. NATALIZUMAB	0.0104	0.002
DMF vs. FINGOLIMOD	0.035	0.0139
DMF vs. NATALIZUMAB	0.021	0.0064
DMF vs. RITUXIMAB/OCRELIZUMAB	0.023	0.0077
DMF LYMPHO vs. FINGOLIMOD	0.0317	0.0115
DMF LYMPHO vs. NATALIZUMAB	0.035	0.0146
DMF LYMPHO vs. RITUXIMAB/OCRELIZUMAB	0.021	0.0064
FINGOLIMOD vs. IFN	0.0169	0.0042
FINGOLIMOD vs. NATALIZUMAB	0.0001	<0.0001
FINGOLIMOD vs. TERFLUNOMIDE	0.0091	0.0013
IFN vs. RITUXIMAB/OCRELIZUMAB	0.0104	0.0023
NATALIZUMAB vs. RITUXIMAB/OCRELIZUMAB	0.0001	<0.0001
TERFLUNOMIDE vs. RITUXIMAB/OCRELIZUMAB	0.0081	0.0007

PB

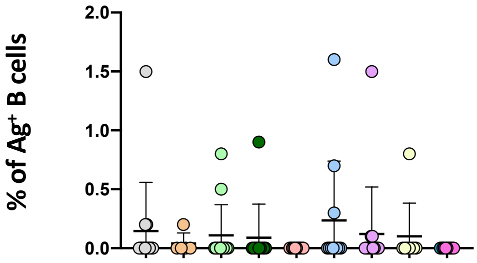

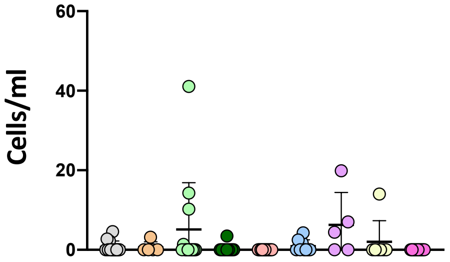

Supplementary Figure 13E. Detailed statistical analysis of 11 clusters obtained using FlowSOM. On the left, dot plots show the percentage of cells in different treated groups of patients and healthy donors (HD). On the right, dot plots show the absolute number of cells in different treated groups of patients and healthy donors (HD). Scatter plots show individual values, mean ± standard deviation (SD). Tables display statistically significant q-value and individual p-value obtained using Kruskal–Wallis test with Benjamini–Hochberg correction for multiple comparisons. Abbreviations: Naïve; TrB. transitional B cells; MBC Usw. memory B cell unswitched; MBC. memory B cell; PB. plasmablasts; atBC. atypical B cell. HD, healthy donors n=13; Cladribine n=6; DMF, Dimethyl Fumarate n=14; DMF Lympho, Dimethyl Fumarate Lymphopenic n=9; Fingolimod n=12; IFN, interferon n=13; Natalizumab n=15; Teriflunomide n=8; rituximab/ocrelizumab (Ocrelizumab;/Rituximab) n=11.

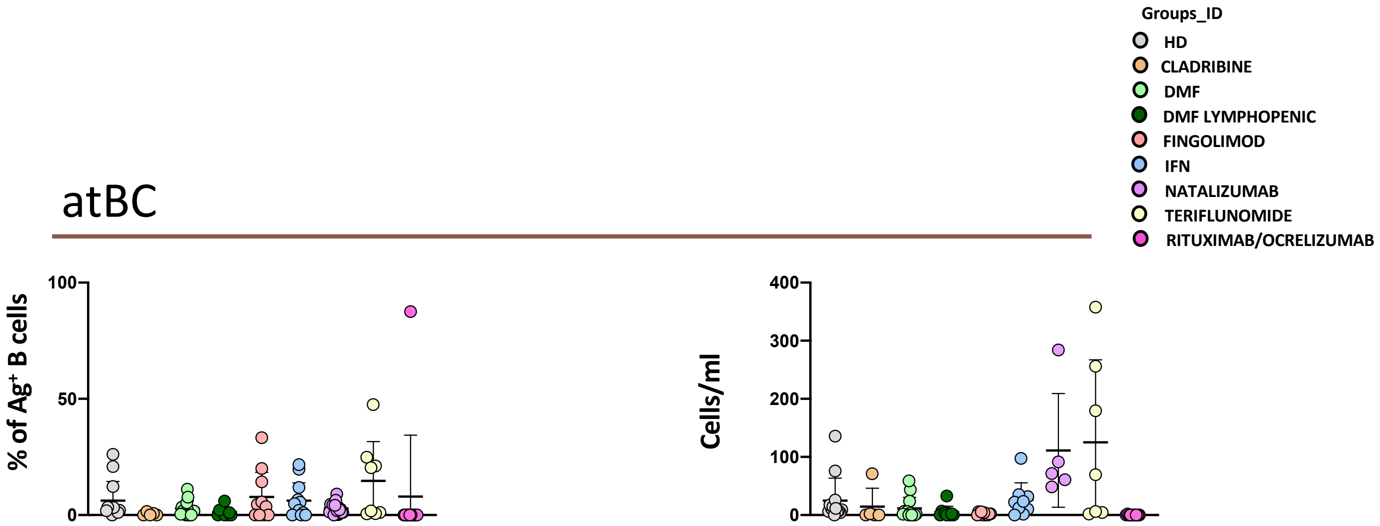

atBC (N, %)	q value	Individual P Value
HD vs. RITUXIMAB/OCRELIZUMAB	0.0128	0.0006
FINGOLIMOD vs. RITUXIMAB/OCRELIZUMAB	0.0308	0.0043
IFN vs. RITUXIMAB/OCRELIZUMAB	0.0308	0.0039
NATALIZUMAB vs. RITUXIMAB/OCRELIZUMAB	0.0308	0.0035
TERFLUNOMIDE vs. RITUXIMAB/OCRELIZUMAB	0.0128	0.0007

atBC (N, mL)	q value	Individual P Value
HD vs. FINGOLIMOD	0.0363	0.0091
HD vs. RITUXIMAB/OCRELIZUMAB	0.0004	<0.0001
CLADRIBINE vs. NATALIZUMAB	0.0114	0.0022
CLADRIBINE vs. TERFLUNOMIDE	0.0392	0.0139
DMF vs. NATALIZUMAB	0.0161	0.004
DMF vs. RITUXIMAB/OCRELIZUMAB	0.0392	0.0142
DMF LYMPHO vs. NATALIZUMAB	0.0061	0.001
DMF LYMPHO vs. TERFLUNOMIDE	0.0291	0.0081
FINGOLIMOD vs. NATALIZUMAB	0.0029	0.0004
FINGOLIMOD vs. TERFLUNOMIDE	0.0155	0.0035
IFN vs. RITUXIMAB/OCRELIZUMAB	0.0022	0.0002
NATALIZUMAB vs. RITUXIMAB/OCRELIZUMAB	0.0001	<0.0001
TERFLUNOMIDE vs. RITUXIMAB/OCRELIZUMAB	0.0006	<0.0001

Supplementary Figure 13F. Detailed statistical analysis of 11 clusters obtained using FlowSOM. On the left, dot plots show the percentage of cells in different treated groups of patients and healthy donors (HD). On the right, dot plots show the absolute number of cells in different treated groups of patients and healthy donors (HD). Scatter plots show individual values, mean ± standard deviation (SD). Tables display statistically significant q-value and individual p-value obtained using Kruskal–Wallis test with Benjamini–Hochberg correction for multiple comparisons. Abbreviations: Naïve; TrB. transitional B cells; MBC Usw. memory B cell unswitched; MBC. memory B cell; PB. plasmablasts; atBC. atypical B cell. HD, healthy donors n=13; Cladribine n=6; DMF, Dimethyl Fumarate n=14; DMF Lympho, Dimethyl Fumarate Lymphopenic n=9; Fingolimod n=12; IFN, interferon n=13; Natalizumab n=15; Teriflunomide n=8; rituximab/ocrelizumab (Ocrelizumab;/Rituximab) n=11.

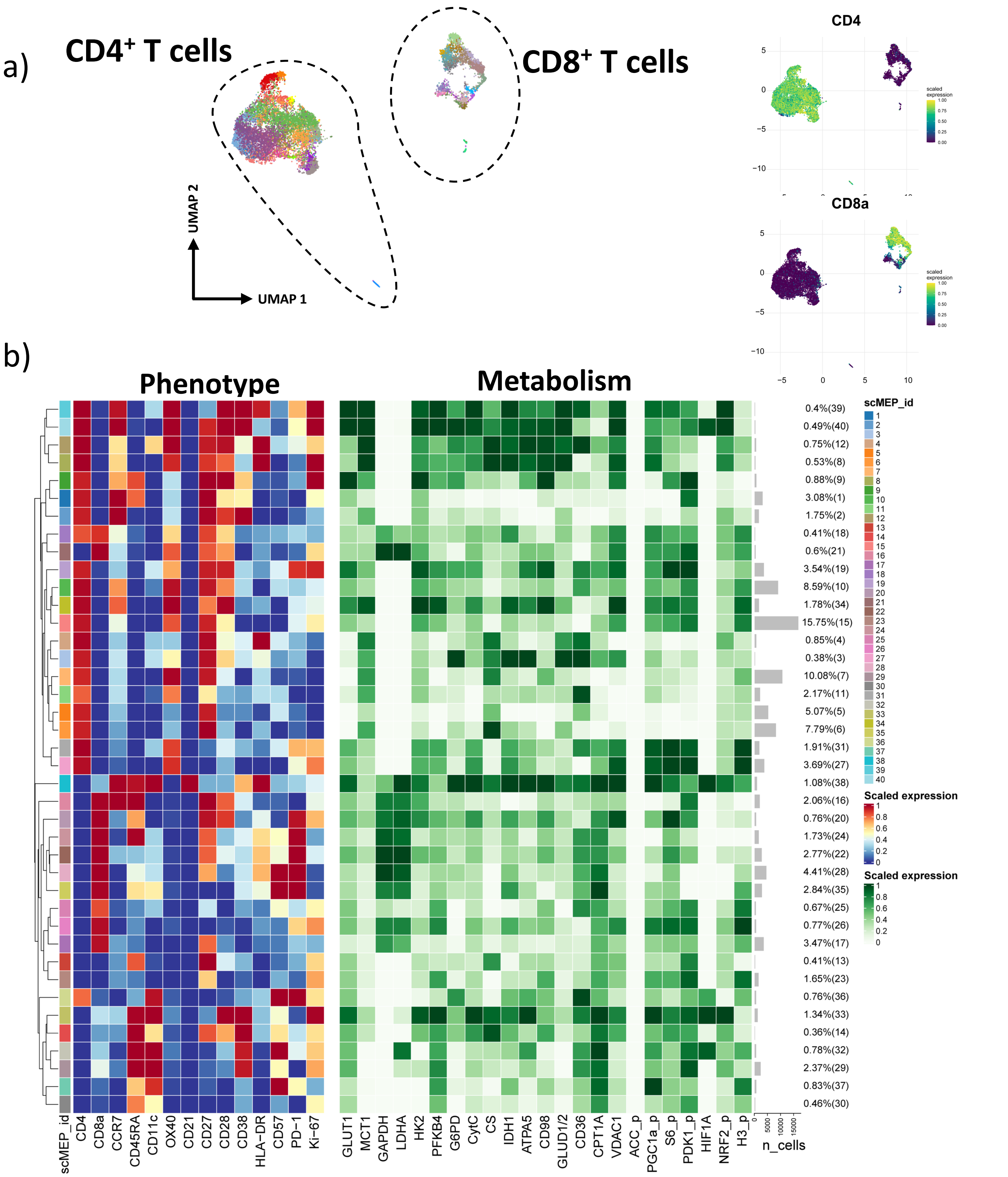

Supplementary Figure 14. **(a)** Uniform Manifold Approximation and Projection (UMAP) representing single-cell metabolic regulome profiling (scMEP) of antigen-specific CD4+ and CD8+ T cells. **(b) (Left)** Heatmap of the median markers intensity of the 14 lineage markers across the antigen-specific T cell populations obtained with FlowSOM algorithm after the manual metaclusters merging. Blue represents lower expression while red represent higher expression. **(Right)** Heatmap of the median markers intensity of the 23 markers of different metabolic pathways. The color in the heatmap is referred to the median of the arcsinh marker expression (0 to 1 scaled) calculated over cells from all the samples. White represents lower expression and less activated metabolic state, while green represents higher expression and more activated metabolic state respectively. Light grey bar along the rows (clusters) and values in brackets indicate the relative sizes of clusters.

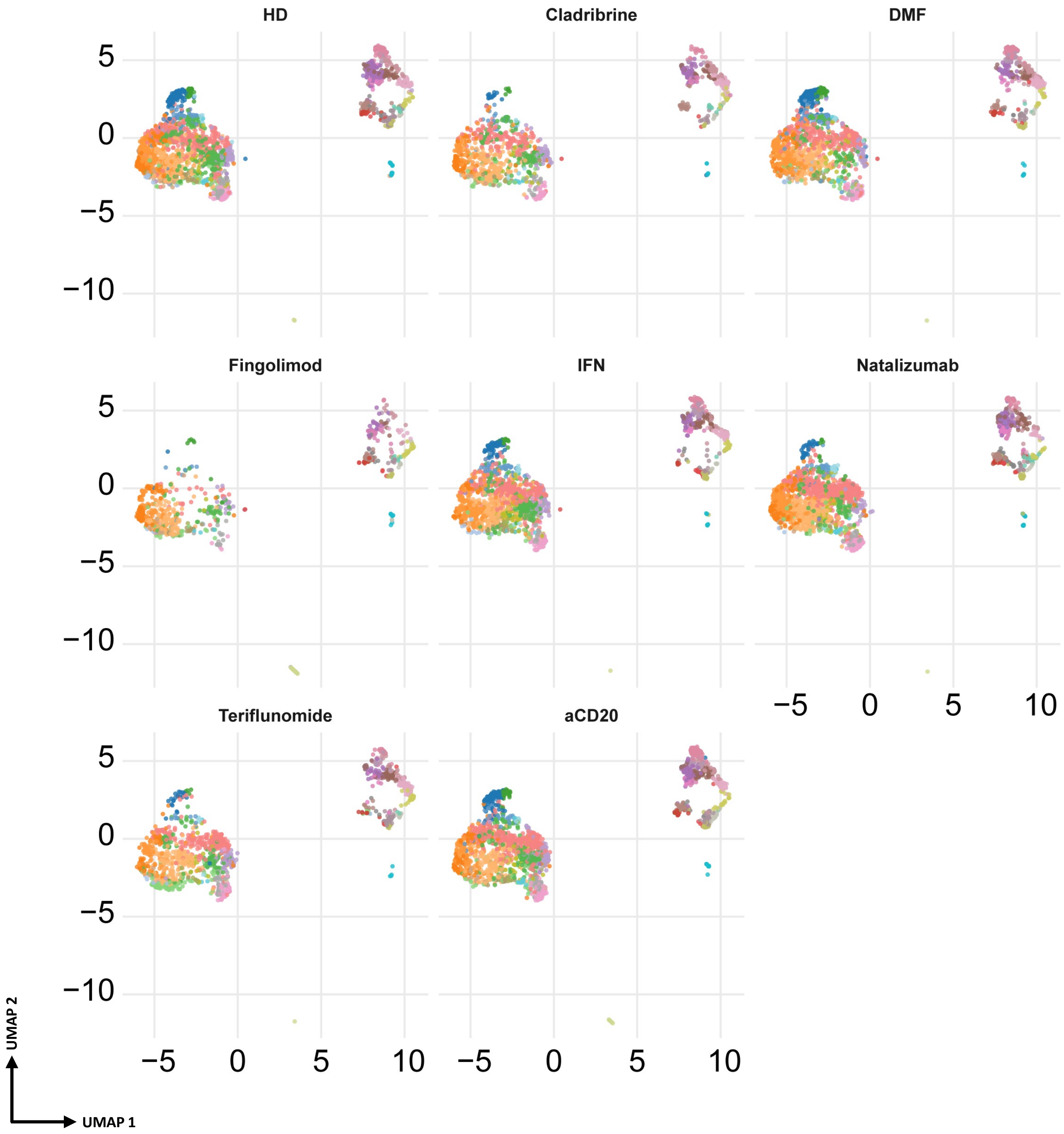

Supplementary Figure 15. Single-cell metabolic regulome profiling (scMEP) of antigen-specific T cells shows the 2D spatial distribution of cells from healthy donors (HD, n= 8), multiple sclerosis patients treated with Cladribine (n=4), DMF (n= 8), Fingolimod (n= 5), IFN (n= 6), Natalizumab (n= 8), Teriflunomide (n= 5), rituximab/ocrelizumab (n= 7).

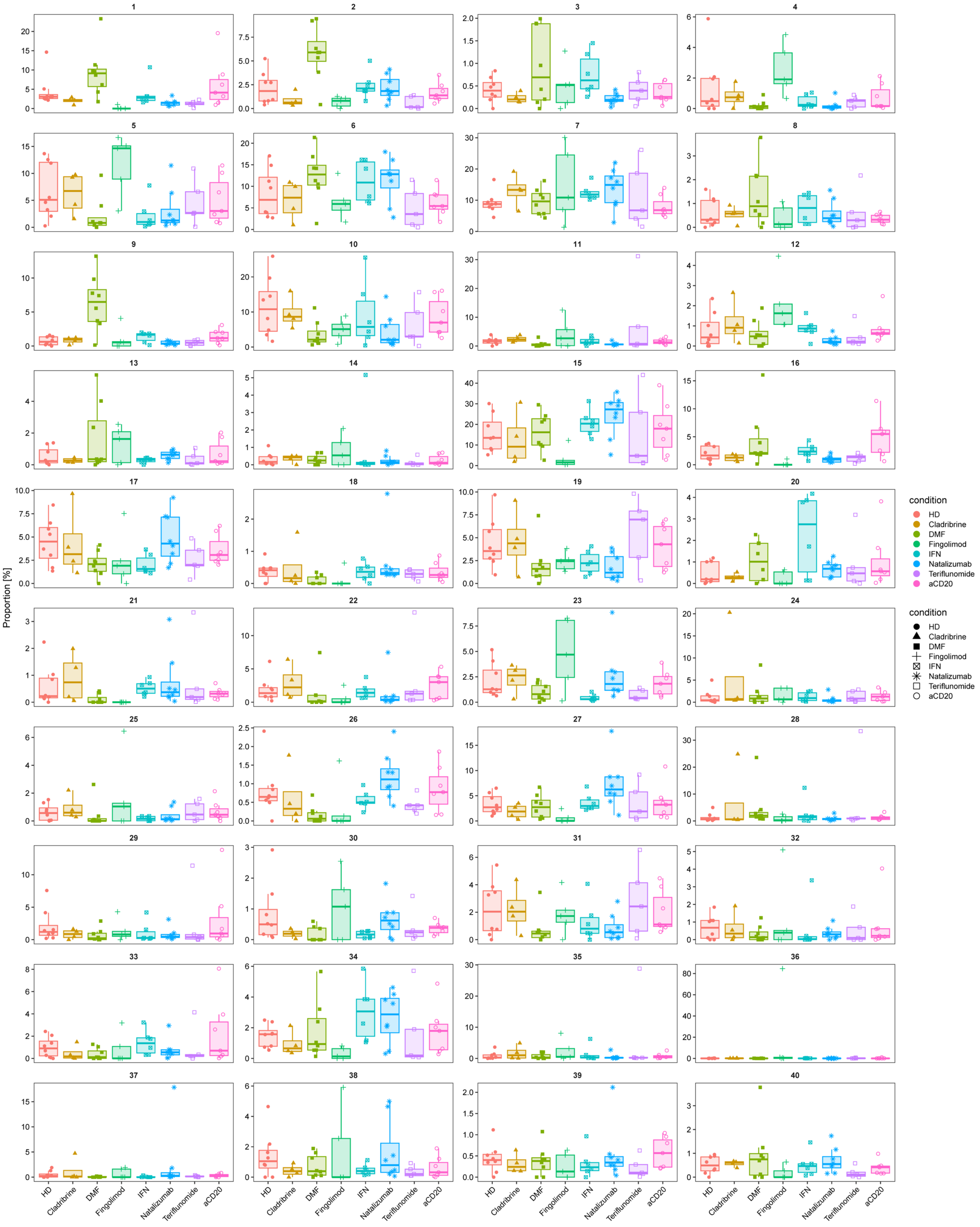

Supplementary Figure 16. Proportions of antigen-specific T cells scMEP of healthy donors (HD, n= 8), multiple sclerosis patients treated with Cladribine (n=4), DMF (n= 8), Fingolimod (n= 5), IFN (n= 6), Natalizumab (n= 8), Teriflunomide (n= 5), rituximab/ocrelizumab (n= 7). Box plots show median value, interquartile ranges (IQR) and whiskers (1.5*IQR).

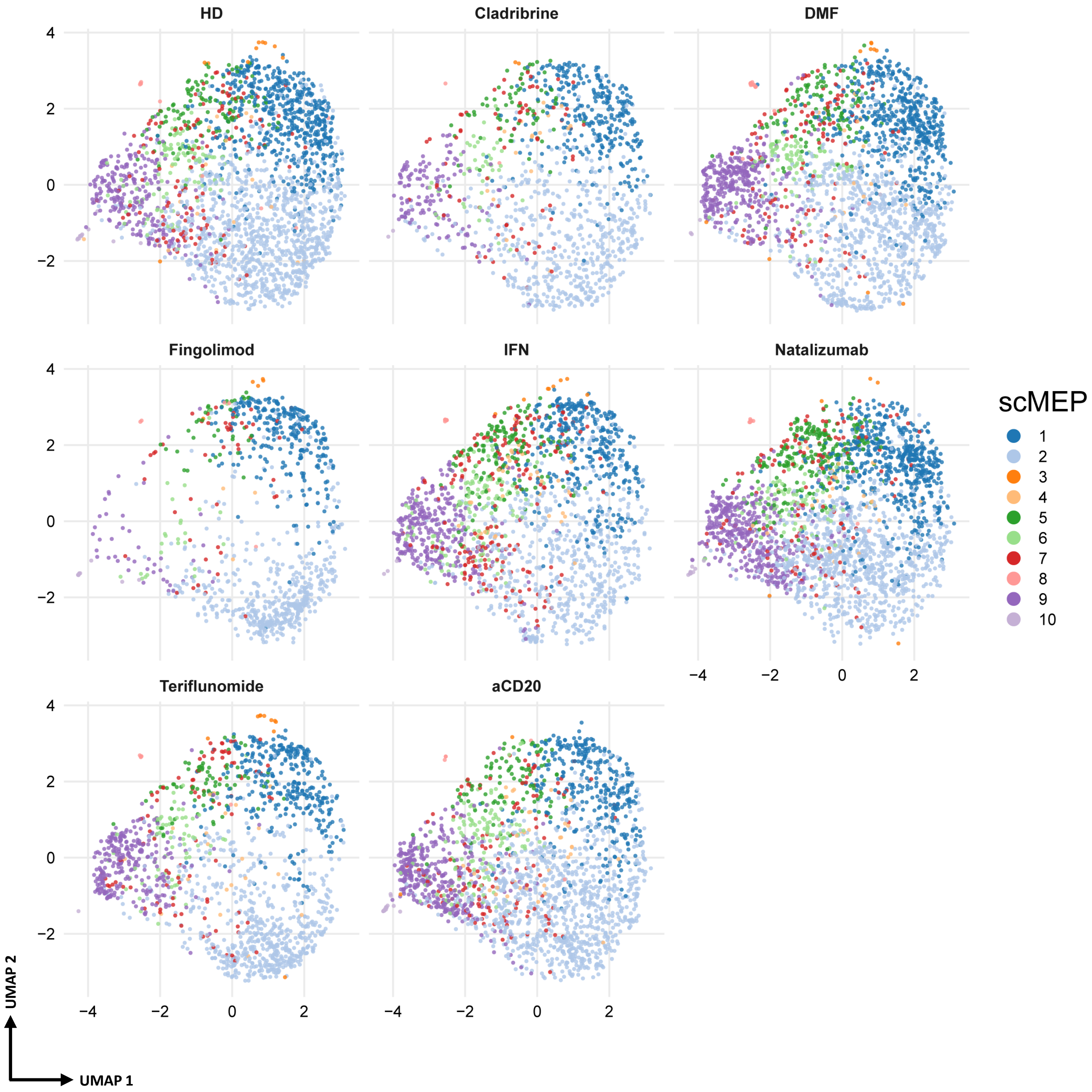

Supplementary Figure 17. Uniform Manifold Approximation and Projection (UMAP) plot representing scMEP of antigen-specific T cells shows the 2D spatial distribution of cells from healthy donors (HD, n= 8), multiple sclerosis patients treated with Cladribine (n=4), DMF (n= 8), Fingolimod (n= 5), IFN (n= 6), Natalizumab (n= 8), Teriflunomide (n= 5), rituximab/ocrelizumab (n= 7).

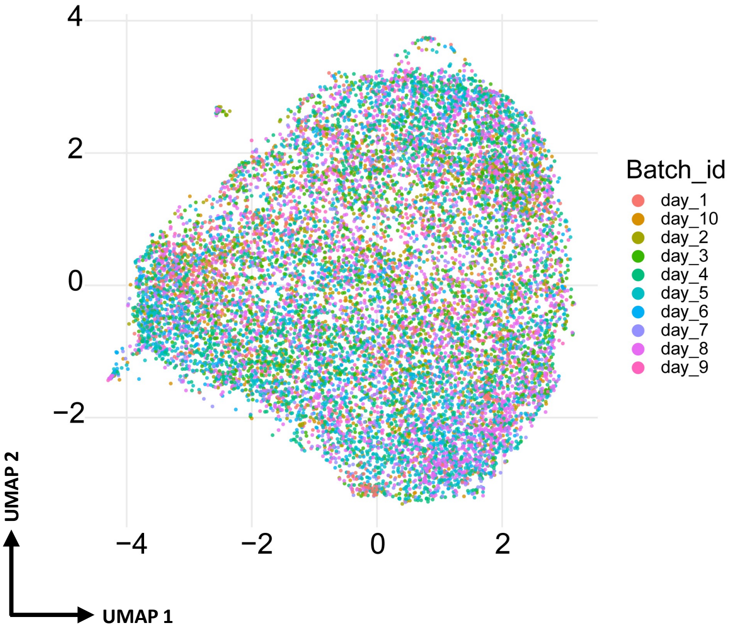

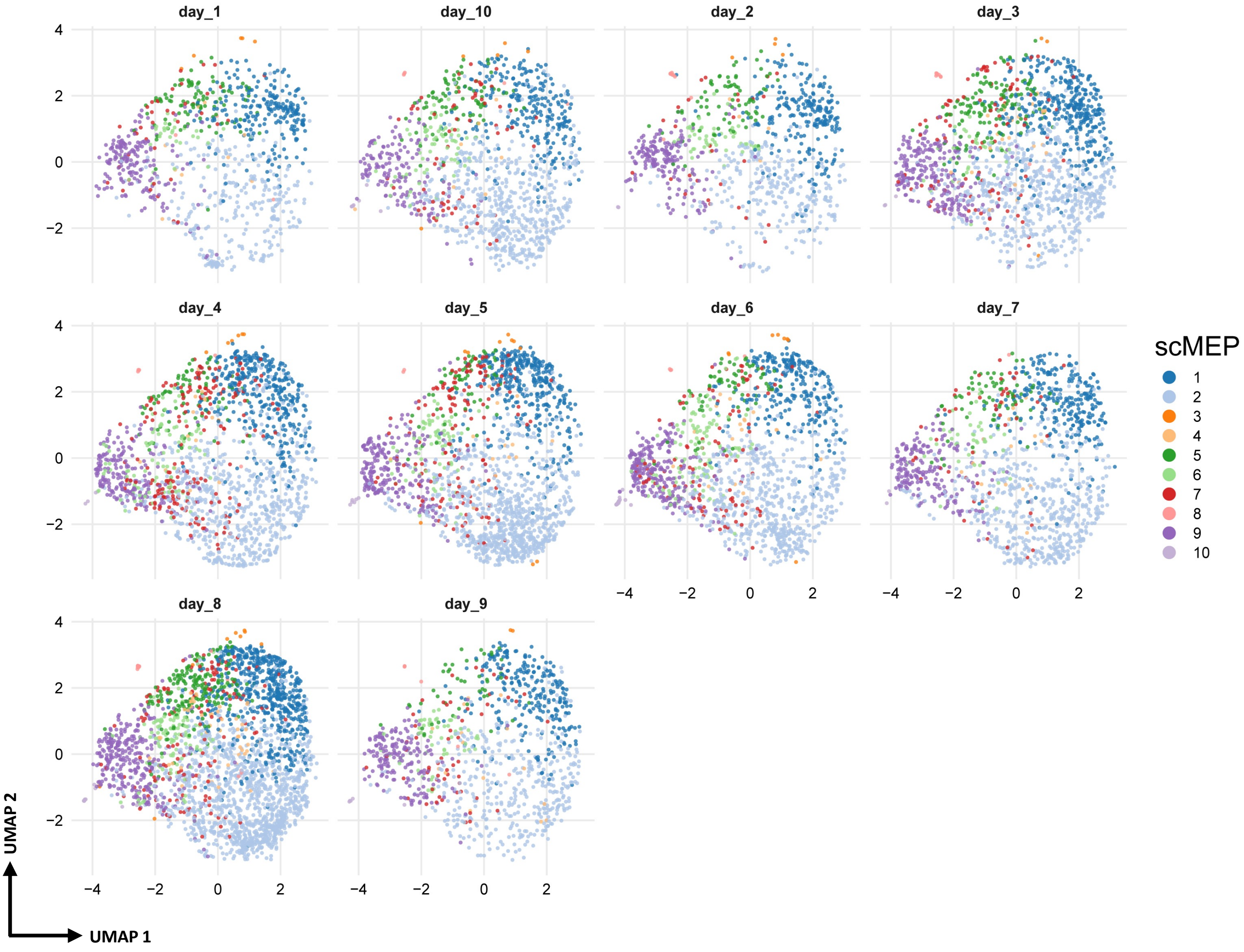

Supplementary Figure 18. Uniform Manifold Approximation and Projection (UMAP) plot representing scMEP of antigen-specific T cells shows the 2D spatial distribution tot cells from healthy donors (HD, n= 8), multiple sclerosis patients treated with Cladribine (n=4), DMF (n= 8), Fingolimod (n= 5), IFN (n= 6), Natalizumab (n= 8), Teriflunomide (n= 5), rituximab/ocrelizumab (n= 7) stratified for batch (different days of acquisition).

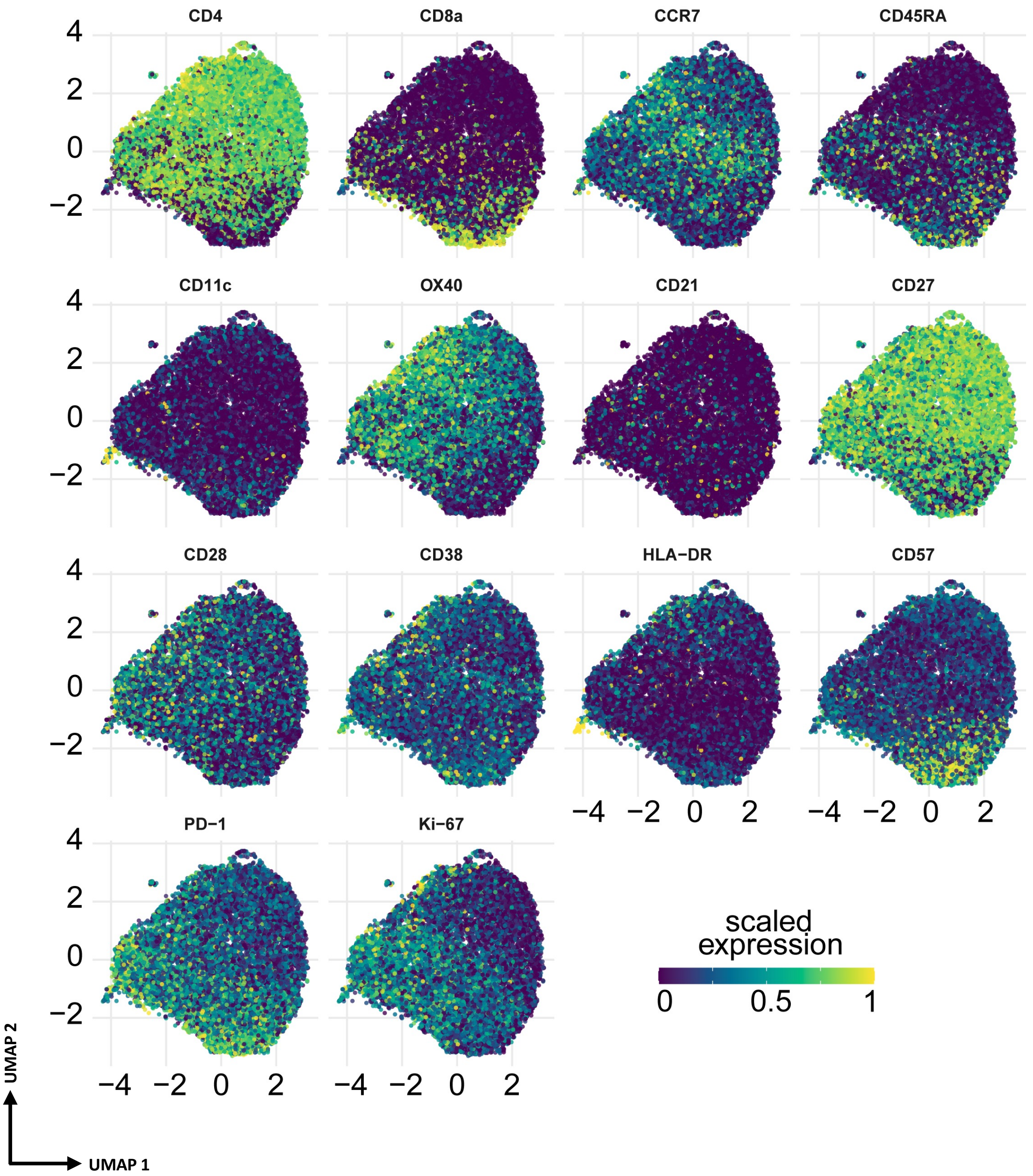

Supplementary Figure 19. UMAP scMEP states of Ag⁺ T cells stratified for lineage markers. Uniform Manifold Approximation and Projection (UMAP) plot representing scMEP of antigen-specific T cells colored by the expression of 14 markers used for T cells phenotyping. Blue represents lower expression while yellow represent higher expression.

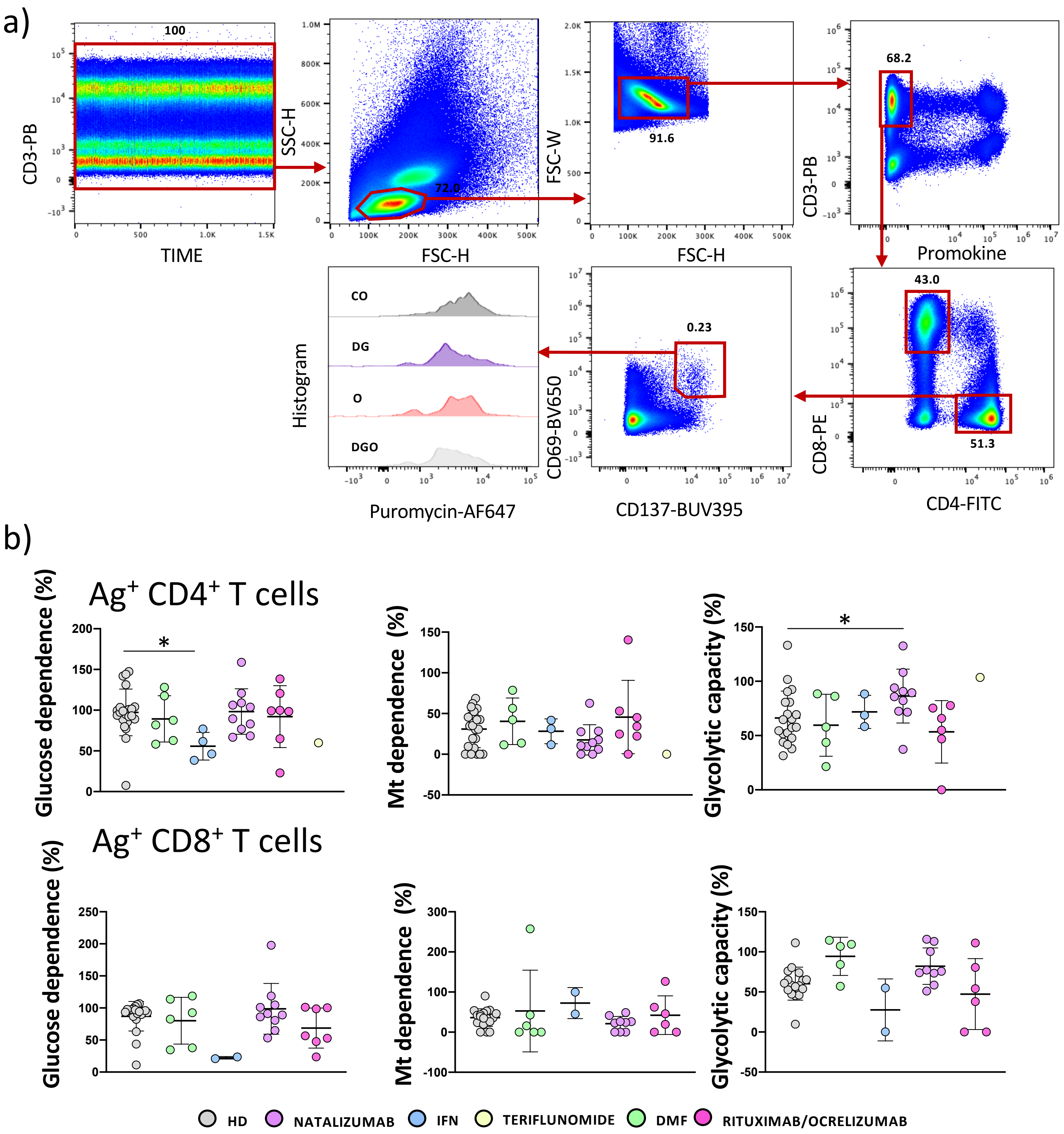

Supplementary Figure 20. **(a)** Gating strategy of antigen-specific T cells. On a bivariate plot of Time vs. CD3, create and place a rectangular region to include all valid events acquired in chronologic homogeneity and avoid fluidic perturbances. Forward and side scatter (FSC and SSC) gating is used to identify cells of interest based on the relative size and complexity of the cells, while removing debris and other events that are not of interest. Further gating is done in an FSC-H and FSC-Width dot plot to eliminate doublets. On a bivariate plot of CD3 vs. Promokine (viability) select CD3$^+$, Promokine$^-$ cells (viable T cells). On a bivariate plot of CD4 vs CD8 select CD8$^+$ or CD4$^+$ T cells and evaluate CD69$^+$ CD137$^+$ Antigen Specific T cells. Within CD69$^+$ CD137$^+$ T cells evaluate the MFI of Puromycin-AF647 after the treatment of different metabolic inhibitors. **(b)** Percentage of glucose dependence, mitochondrial dependence and glycolytic capacity of antigen-specific CD4$^+$ and CD8$^+$ T cells among the different groups. HD, healthy controls; DMF, Dimethyl Fumarate; IFN, interferon; Natalizumab; Teriflunomide; RITUXIMAB/OCRELIZUMAB (Ocrelizumab; Rituximab).

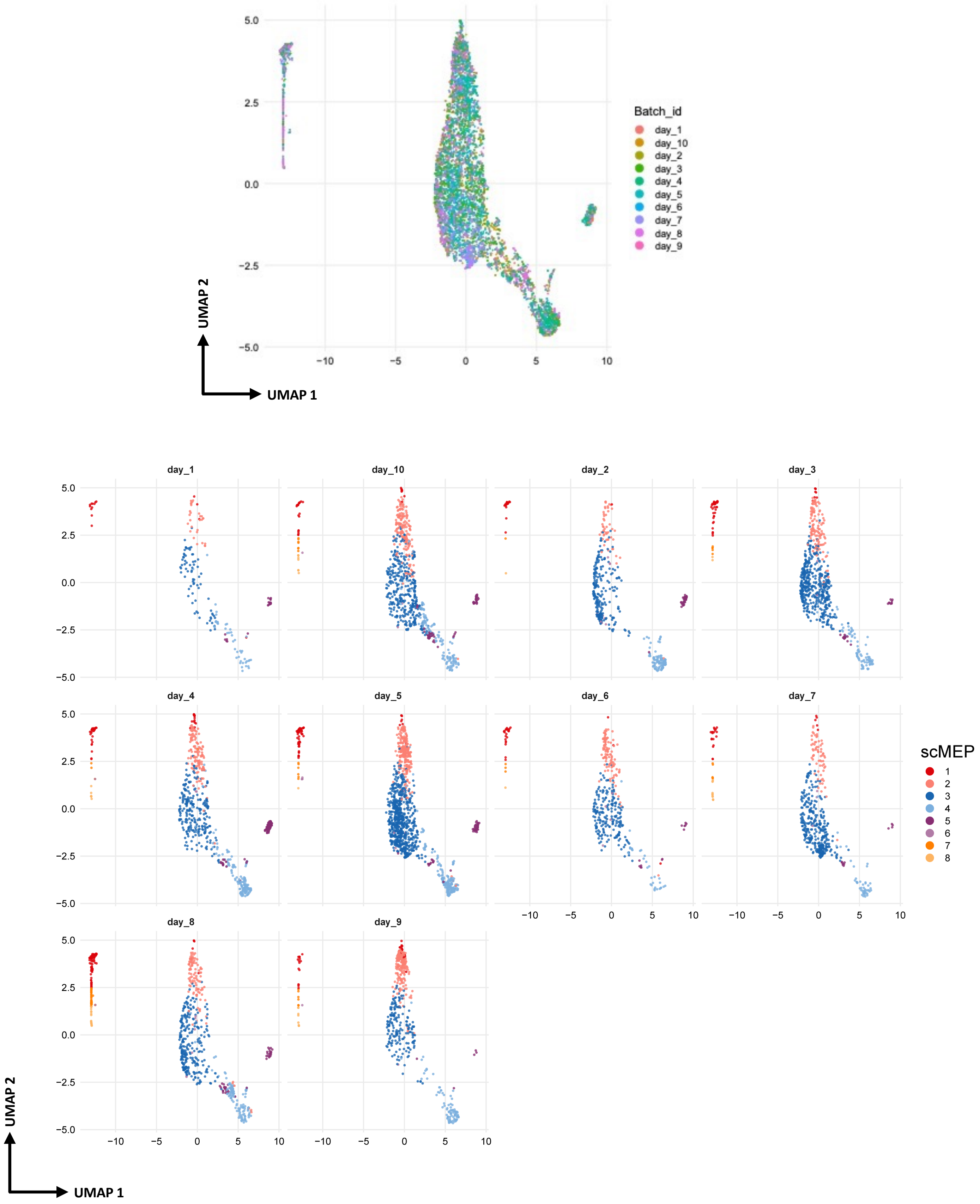

Supplementary Figure 21. Uniform Manifold Approximation and Projection (UMAP) plot representing scMEP of antigen-specific B cells shows the 2D spatial distribution of cells from healthy donors (HD, n= 8), multiple sclerosis patients treated with Cladribine (n=4), DMF (n= 8), Fingolimod (n= 5), IFN (n= 6), Natalizumab (n= 8), Teriflunomide (n= 5), rituximab/ocrelizumab (n= 7) stratified for batch.

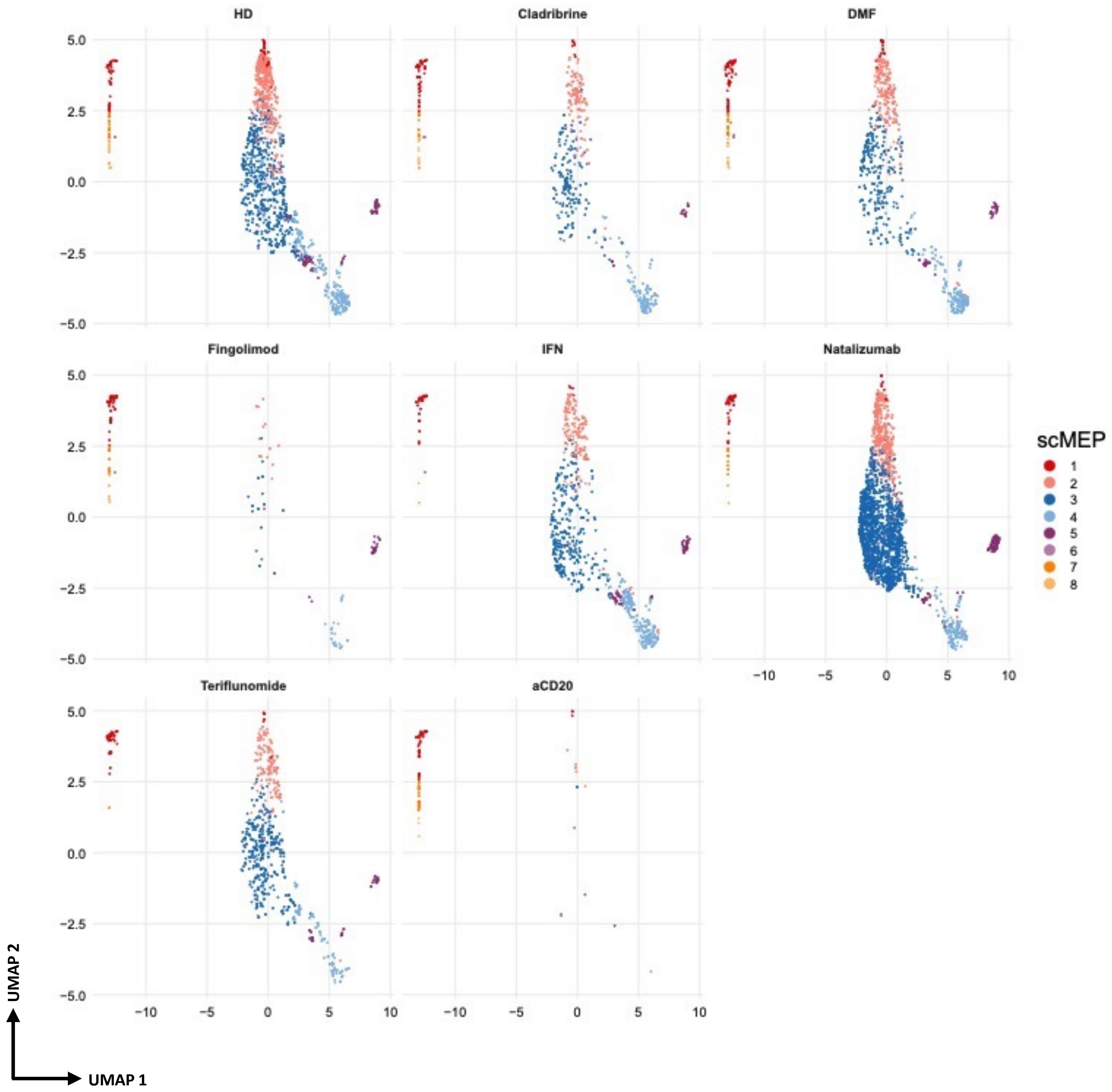

Supplementary Figure 22. Uniform Manifold Approximation and Projection (UMAP) plot representing scMEP of antigen-specific B cells shows the 2D spatial distribution of cells from healthy donors (HD, n= 8), multiple sclerosis patients treated with Cladribine (n=4), DMF (n= 8), Fingolimod (n= 5), IFN (n= 6), Natalizumab (n= 8), Teriflunomide (n= 5), rituximab/ocrelizumab (n= 7), stratified by therapy.

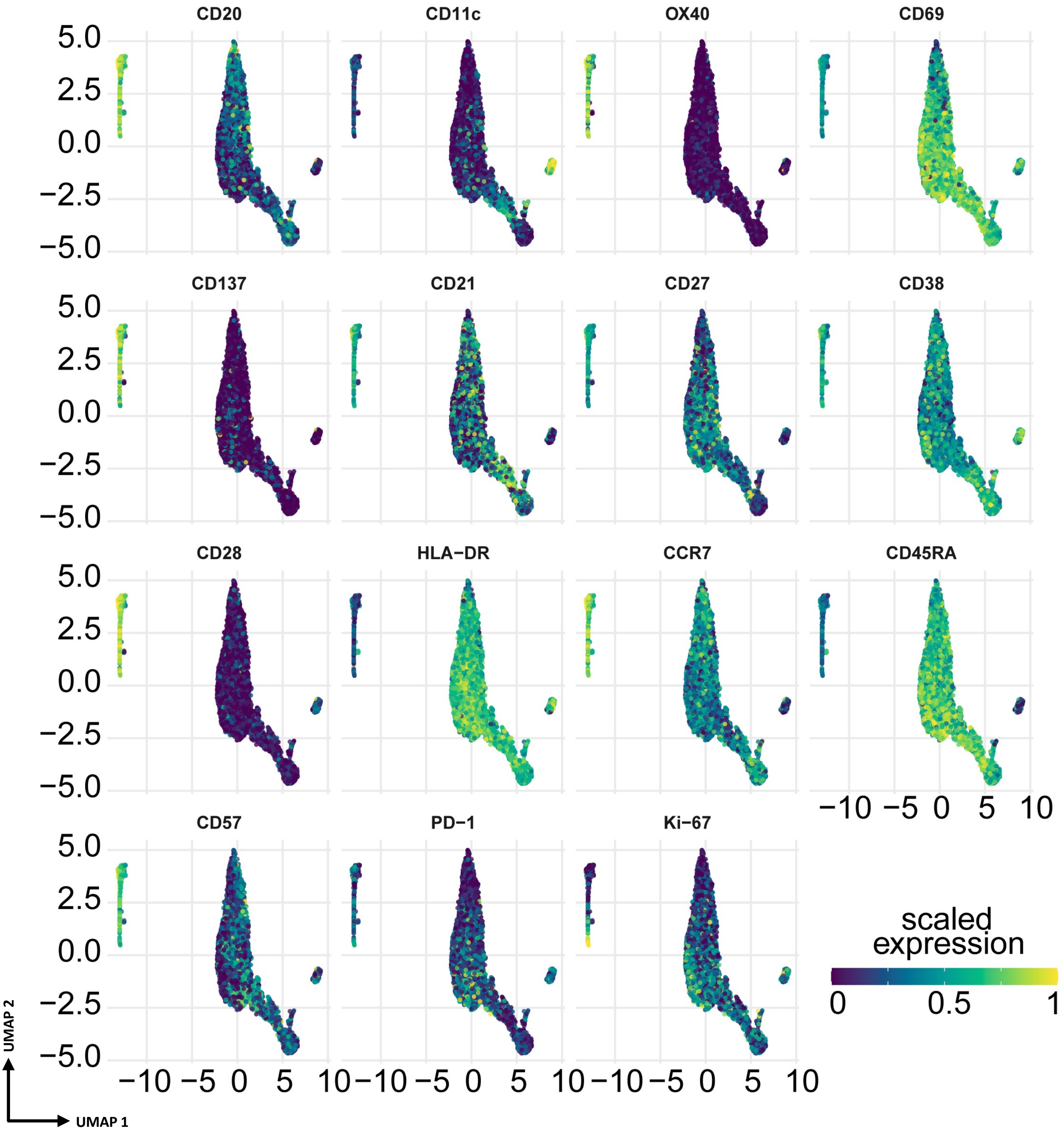

Supplementary Figure 23. **Ag⁺ B cells stratified by lineage markers.** Uniform Manifold Approximation and Projection (UMAP) plot shows the 2D spatial distribution of scMEP of antigen-specific B cells, colored by the expression of 15 markers used for B cells phenotype. Blue represents lower expression while yellow represent higher expression.

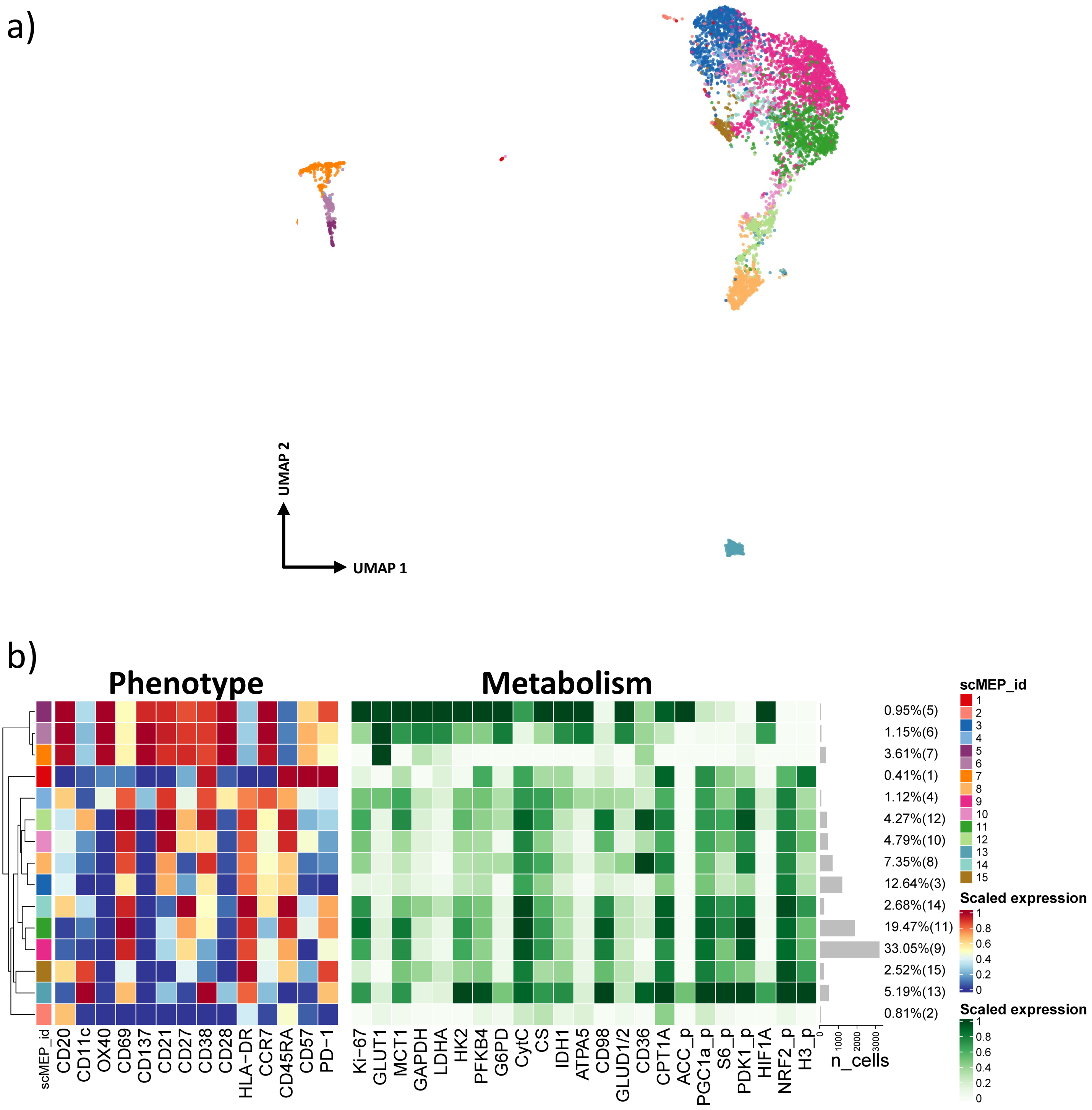

Supplementary Figure 24. **(a)** Uniform Manifold Approximation and Projection (UMAP) representing single-cell metabolic regulome profiling (scMEP) of antigen-specific B cells **(b) (Left)** Heatmap of the median markers intensity of the 15 lineage markers across the antigen-specific B cell populations obtained with FlowSOM algorithm after the manual metaclusters merging. Blue represents lower expression while red represent higher expression. **(Right)** Heatmap of the median markers intensity of the 23 markers of different metabolic pathways. White represents lower expression and less activated metabolic state, while green represents higher expression and more activated metabolic state respectively. Light grey bar along the rows (clusters) and values in brackets indicate the relative sizes of clusters.

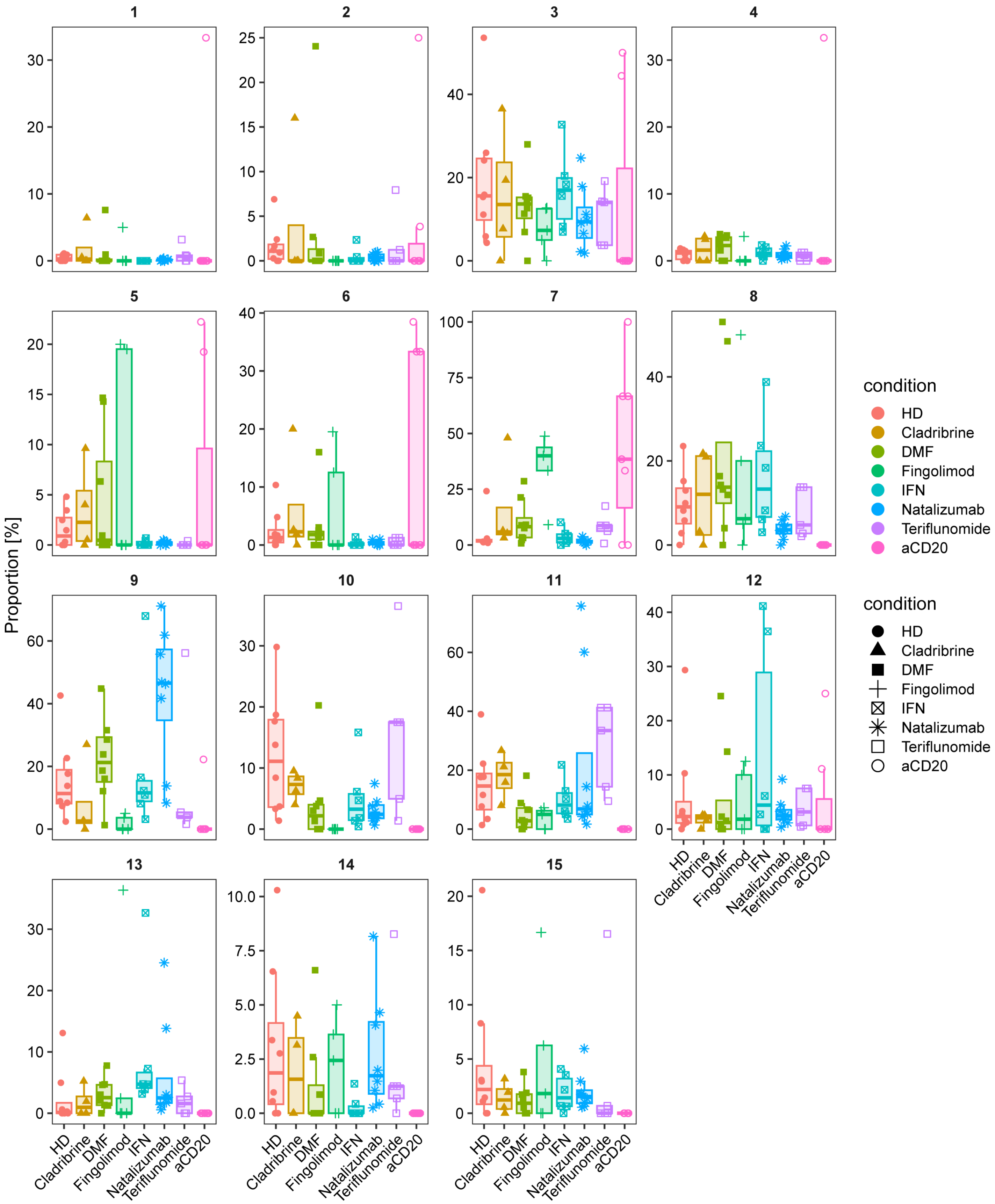

Supplementary Figure 25. Proportions of antigen-specific B cells scMEP of healthy donors (HD, n= 8), multiple sclerosis patients treated with Cladribine (n=4), DMF (n= 8), Fingolimod (n= 5), IFN (n= 6), Natalizumab (n= 8), Teriflunomide (n= 5), rituximab/ocrelizumab (n= 7). Box plots show median value, interquartile ranges (IQR) and whiskers (1.5*IQR).

a)

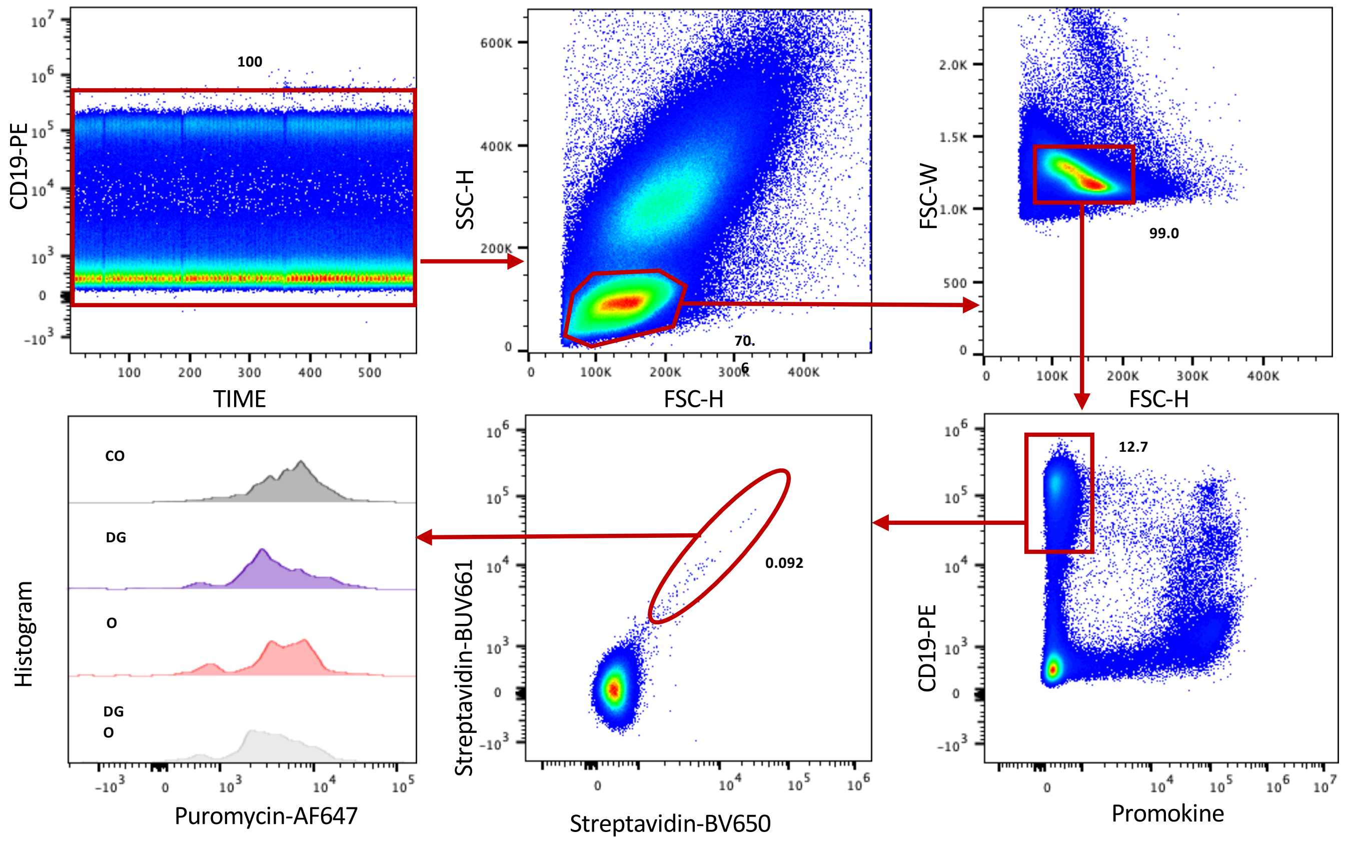

b)

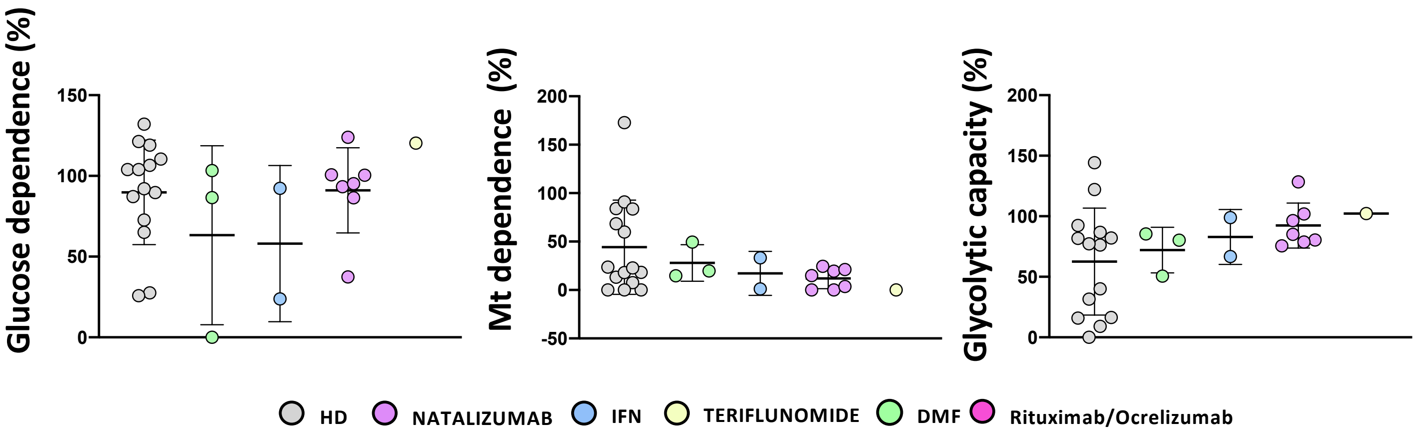

Supplementary Figure 26. **(a)** Gating strategy of antigen-specific B cells. On a bivariate plot of Time vs. CD19, create and place a rectangular region to include all valid events acquired in chronologic homogeneity and avoid fluidic perturbances. Forward and side scatter (FSC and SSC) gating is used to identify cells of interest based on the relative size and complexity of the cells, while removing debris and other events that are not of interest. Further gating is done in an FSC-H and FSC-Width dot plot to eliminate doublets. On a bivariate plot of CD19 vs. Promokine (viability) select CD19^{+}, Promokine^{-} cells (viable B cells) and evaluate Streptavidin-BUV661^{+} Streptavidin-BV650^{+} Antigen Specific B cells. Within antigen-specific B cells evaluate the MFI of Puromycin-AF647 after the treatment of different metabolic inhibitors. **(b)** Percentage of glucose dependence, mitochondrial dependence and glycolytic capacity of antigen-specific B cells among the different groups. HD, healthy controls; DMF, Dimethyl Fumarate; IFN, interferon; Natalizumab; Teriflunomide; RITUXIMAB/OCRELIZUMAB (Ocrelizumab; Rituximab).

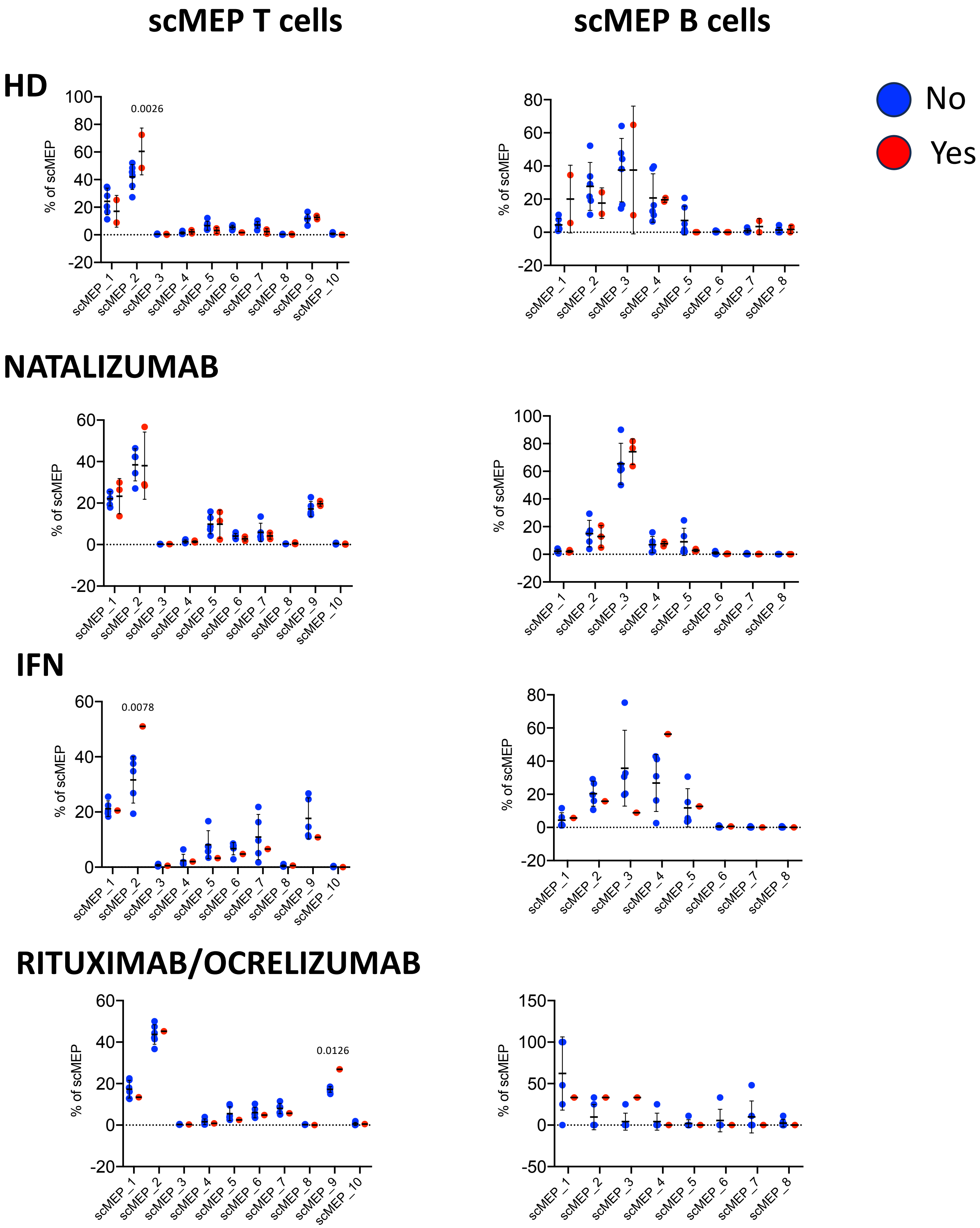

Supplementary Figure 27. The percentage of scMEP clusters divided by who experienced SARS-CoV-2 breakthrough infection (YES, in red dots) and who did not (NO, in blue dots), stratified by therapy. Column on the left reports percentages of T cell clusters, column on the right reports percentages of B cell clusters. Mean and SD are represented, Two-way ANOVA test. Exact q value is reported in the figure.

Supplementary Table 1. mAbs used in AIM assay.

Target	Dye	Clone	Producer	Catalog number	Lot number	Titer (µL)/100µL
**PromoFluor840**	Maleimide	N/A	Promocell	PK-PF840-3- 01		0.3
**CD45RA**	FITC	2H4	Beckman Coulter (DuraClone IM T)	B53328		-
**CCR7**	PE	G043H7	Beckman Coulter (DuraClone IM T)	B53328		-
**CD28**	ECD	CD28.2	Beckman Coulter (DuraClone IM T)	B53328		-
**PD-1**	PC5.5	PD1.3.5	Beckman Coulter (DuraClone IM T)	B53328		-
**CD27**	PC7	1A4.CD2 7	Beckman Coulter (DuraClone IM T)	B53328		-
**CD4**	APC	13B8.2	Beckman Coulter (DuraClone IM T)	B53328		-
**CD8**	A700	B9.11	Beckman Coulter (DuraClone IM T)	B53328		-
**CD3**	APC-A750	UCHT-1	Beckman Coulter (DuraClone IM T)	B53328		-
**CD57**	Pacific Blue	NC1	Beckman Coulter (DuraClone IM T)	B53328		-
**CD45**	Krome Orange	J33	Beckman Coulter (DuraClone IM T)	B53328		-
**CXCR3**	BV785	G025H7	BioLegend	353738	B302668	1.25
**CCR6**	BUV496	11A9	Becton Dickinson	612948	1114714	1.25
**CXCR5**	BUV661	RF8B2	Becton Dickinson	741559	1298915	0.6
**CD69**	BV650	FN50	BioLegend	310934	B346313	2.5
**CD137**	BUV395	4B4-1	Becton Dickinson	745737	1298922	1.25
**CD95**	BV605	DX2	BioLegend	305628	B344380	2.5

Supplementary Table 2. mAbs used in ICS protocol

TARGET	DYE	CLONE	PRODUCER	CATALOG NUMBER	LOT NUMBER	TITER µL/100µL
**LIVE/DEAD**	AQUA	N/A	ThermoFisher	L34966	2268307	1.25
**CD4**	AF700	RPA-T4	Biolegend	300526	B336913	0.6
**CD8**	APC-Cy7	RPA-T8	Biolegend	301016	B300873	0.6
**CD3**	PE-Cy5	UCHT1	Biolegend	301016	B300873	0.6
**IFNg**	FITC	B27	Biolegend	506504	B286029	2.5
**TNF**	BV605	MAb11	Biolegend	502936	B327946	3.75
**IL-2**	APC	MQ1-17H12	Biolegend	500310	B313276	2.5
**IL-17a**	PE-Cy7	BL168	Biolegend	512315	B325831	3.75
**GRZB**	BV421	QA18A28	Biolegend	396414	B311965	2.5
**CD107a**	PE	H4A3	Biolegend	328608	B321484	0.3

Supplementary Table 3. mAbs used in B cell panel.

Target	Dye	Clone	Producer	Catalog Number	Lot Number	Titer µL/100µL
**PromoFluor840**	Maleimide	N/A	Promocell	PK-PF840-3- 01		0.3
**CD45**	Krome Orange	J33	Beckman Coulter (DuraClone IM B)	B53318		-
**CD19**	ECD	J3-119	Beckman Coulter (DuraClone IM B)	B53318		-
**CD21**	PE	BL13	Beckman Coulter (DuraClone IM B)	B53318		-
**CD27**	PC7	1A4CD27	Beckman Coulter (DuraClone IM B)	B53318		-
**CD24**	APC	ALB9	Beckman Coulter (DuraClone IM B)	B53318		-
**CD38**	APC-A750	LS198-4-3	Beckman Coulter (DuraClone IM B)	B53318		-
**IgD**	FITC	IA6-2	Beckman Coulter (DuraClone IM B)	B53318		-
**IgM**	Pacific Blue	SA-DA4	Beckman Coulter (DuraClone IM B)	B53318		-
**Streptavidin**	BV650	-	BioLegend	405231	B347044	0.3
**Streptavidin**	BUV661	-	Becton Dickinson	612979	1188291	0.3
**Streptavidin**	AF700	-	ThermoFisher	S21383	2286302	0.1
**S-protein**	Biotin	-	R&D	BT10549	DOJH0421071	4.5
**CD20**	BV785	2H7	BioLegend	302356	B337363	0.6
**CD71**	BUV395	M-A712	Becton Dickinson	743308	1341511	1.25
**IgG**	BUV496	G18-154	Becton Dickinson	741172	1341490	1.25
**IgA**	PerCP-Vio700	1S11-8E10	Miltenyi Biotec	130-113-478	5211109889	0.5

Supplementary Table 4. mAbs used in SCENITH protocol

Specificity	Dye	Clone	Manufacturer	Cat.	Lot	Titer µL/100µL
**Fc-Block**	-	-	Beckton Dickinson	564220	0114728	0.5
**PromoFluor-840**	Maleimide	-	PromoKine	PK-PF840-3-01	429P0-17	0.3
**CD19**	PE	HIB19	Biolegend	302208	B355446	2
**CD69**	FITC	FN50	Biolegend	310904	B347085	2.5
**CD4**	FITC	RPA-T4	Biolegend	300506	B283935	2
**CD8**	PE	RPA-T8	Biolegend	301008	B323647	0.6
**CD8**	APC-Cy7	RPA-T8	Biolegend	301016	B300873	0.6
**CD3**	PB	UCHT1	Beckton Dickinson	558117	1180049	2.5
**CD69**	BV650	FN50	Biolegend	310934	B356230	2.5
**CD137**	BUV395	4B4-1	Beckton Dickinson	745739	2171939	1.25
**Puromycin**	AF647					1/250

Supplementary Table 5. mAbs used in CYTOF scMEP panel.

MARKER	label	clone	brand	cat	lot	titer (µL/100µL)
**CD45**	089Y	HI30	Standard Biotools	3089003B	2208767-16	1
**CD4**	106Cd	RPA-T4	Biolegend	300502	custom	1
**HLA-DR**	110Cd	L243	Biolegend	307602	custom	1
**CD8a**	111Cd	RPA-T8	Biolegend	301002	custom	1
**GAPDH**	112Cd	6C5	ThermoFisher	AM4300	custom	1
**CD20**	113Cd	2H7	Biolegend	302302	custom	1
**LDHA**	114Cd	EP1566Y	AbCam	ab219591	custom	1
**NRF2_p**	116Cd	EP1809Y	AbCam	ab180844	custom	1
**CD3**	141Pr	UCHT1	Standard Biotools	3141019B	2112251-08	1
**CD19**	142Nd	HIB19	Standard Biotools	3142001B	2203508-10	1
**CD45RA**	143Nd	HI100	Standard Biotools	3143006B	2209433-27	1
**CD38**	144Nd	HIT2	Standard Biotools	3144014B	2203499-09	1
**CytC**	145Nd	6H2.B4	Biolegend	612302	custom	1
**CS**	146Nd	EPR8067	Abcam	ab233838	custom	1
**CD11c**	147Sm	Bu15	Standard Biotools	3147008B	3431914	1
**HIF1A**	148Nd	700505	Thermofisher	16H4L13	custom	1
**PFKFB4**	149Sm	PA528648	Thermofisher	PA5-28648	custom	1
**CD134**	150Nd	ACT35	Standard Biotools	3150023B	2112513-23	1
**ACC_p**	151Eu	D7D11	Cell Signaling	11818S	custom	1
**CD21**	152Sm	BL13	Standard Biotools	3152010B	2205540-11	1
**ATPA5**	153Eu	15H4C4	Abcam	ab14748	custom	1
**VDAC1**	154Sm	20B12AF2	Abcam	ab14734	custom	1
**CD36**	155Gd	5-271	Standard Biotools	3155012B	1151904	1
**G6PD**	156Gd	EPR20668	Abcam	ab231828	custom	1
**CD27**	158Gd	L128	Standard Biotools	3158010B	2206891-08	1
**CD98**	159Tb	UM7F8	Standard Biotools	3159022B	1551501	1
**CD28**	160Gd	CD28.2	Standard Biotools	3160003B	2206133-22	1
**Ki-67**	161Dy	B56	Standard Biotools	3161007B	2202239-18	1
**CD69**	162Dy	FN50	Standard Biotools	3162001B	2112484-22	1
**PDK1_p**	163Dy	2H3AA11	Abcam	ab110335	custom	1
**GLUT1**	164Dy	EPR3915	Abcam	ab196357	custom	1
**PGC1a_p**	165Ho	Polyclonal	Novus	NBP1-04676	custom	1
**HK2**	166Er	EPR20839	Abcam	ab228819	custom	1
**CD197**	167Er	G043H7	Standard Biotools	3167009A	2205601-16	1
**MCT1**	168Er	ERR13706(B)	Abcam	ab250131	custom	1
**GLUD1/2**	169Tm	D9F7P	Cell Signaling	12793S	custom	1
**IDH1**	170Er	843219	Novus	MAB7049	custom	1
**anti-spike**	171Yb	-	R&D	10549-CV	DODR0622071	1
**pS6 [S235/S236]**	172Yb	A17020B	Biolegend	608602	B333307	1
**CPT1A**	173Yb	8F6AE9	Abcam	ab128568	custom	1
**CD279 (PD-1)**	174Yb	EH12.2H7	Standard Biotools	3155009B	2206216-29	1
**pHistone H3 [S28]**	175Lu	HTA28	Standard Biotools	3175012A	2208977-31	1
**CD57**	176Yb	HNK-1	Biolegend	359602	B338514	1
**CD137**	209Bi	4B4-1	Standard Biotools	3209015B	2203423-07	1
**Cell-ID Cisplatin**	195Pt	-	Standard Biotools	201195	2203477-08	0.5
**Cell-ID Intercalator**	191&193Ir	-	Standard Biotools	201192A	2204312-27	see protocol
