# Supplementary material for: Immunosenescence and vaccine efficacy revealed by immunometabolic analysis of SARS-CoV-2-specific cells in multiple sclerosis patients
Source: Nat Commun. 2024 Mar 29;15:2752. doi: 10.1038/s41467-024-47013-0 (PMC10980723; doi:10.1038/s41467-024-47013-0)
Supplement: Supplementary file 4 — Description of Additional Supplementary Files [file 41467_2024_47013_MOESM4_ESM.pdf]

Description of Additional Supplementary Files

Supplementary Data 1

Description: Table with demographic and clinical characteristic of healthy donors and MS patients.
