# Supplementary material for: Immunosenescence and vaccine efficacy revealed by immunometabolic analysis of SARS-CoV-2-specific cells in multiple sclerosis patients
Source: Nat Commun. 2024 Mar 29;15:2752. doi: 10.1038/s41467-024-47013-0 (PMC10980723; doi:10.1038/s41467-024-47013-0)
Supplement: Supplementary file 5 — Reporting Summary [file 41467_2024_47013_MOESM5_ESM.pdf]

nature portfolio

Corresponding author(s): Sara De Biasi; Andrea Cossarizza

Last updated by author(s): Feb 15, 2024

Reporting Summary

Nature Portfolio wishes to improve the reproducibility of the work that we publish. This form provides structure for consistency and transparency in reporting. For further information on Nature Portfolio policies, see our Editorial Policies and the Editorial Policy Checklist.

Statistics

For all statistical analyses, confirm that the following items are present in the figure legend, table legend, main text, or Methods section.

n/a	Confirmed	
☐	☒	The exact sample size (*n*) for each experimental group/condition, given as a discrete number and unit of measurement
☐	☒	A statement on whether measurements were taken from distinct samples or whether the same sample was measured repeatedly
☐	☒	The statistical test(s) used AND whether they are one- or two-sided<br>*Only common tests should be described solely by name; describe more complex techniques in the Methods section.*
☒	☐	A description of all covariates tested
☐	☒	A description of any assumptions or corrections, such as tests of normality and adjustment for multiple comparisons
☐	☒	A full description of the statistical parameters including central tendency (e.g. means) or other basic estimates (e.g. regression coefficient) AND variation (e.g. standard deviation) or associated estimates of uncertainty (e.g. confidence intervals)
☐	☒	For null hypothesis testing, the test statistic (e.g. *F*, *t*, *r*) with confidence intervals, effect sizes, degrees of freedom and *P* value noted<br>*Give P values as exact values whenever suitable.*
☒	☐	For Bayesian analysis, information on the choice of priors and Markov chain Monte Carlo settings
☒	☐	For hierarchical and complex designs, identification of the appropriate level for tests and full reporting of outcomes
☒	☐	Estimates of effect sizes (e.g. Cohen's *d*, Pearson's *r*), indicating how they were calculated

Our web collection on statistics for biologists contains articles on many of the points above.

Software and code

Policy information about availability of computer code

Data collection	All data (clinical and data from immunological assays) were collected in Excel v. 16 MAC version.
Data analysis	Quantitative variables were compared using Kruskal-Wallis non-parametric test corrected for multiple comparisons by controlling the False Discovery Rate (FDR), method of Benjamini and Hochberg. Statistically significant q-values are represented. Statistical analysis of cytokines production was performed using GraphPad Prism version 8 (GraphPad Software Inc., La Jolla, USA). Total percentage of antigen-specific (Ag +CD4+ and Ag+CD8+) T cell data have been calculated as background subtracted data. Simplified Presentation of Incredibly Complex Evaluation (SPICE) software (version 6, Vaccine Research Center, NIAID, NIH, Bethesda, MD, USA) was used to analyze flow cytometry data on T cell polyfunctionality. Data from the total cytokine production are represented as individual values, means, and standard errors of the mean. Regarding polyfunctionality, data in pie charts are represented as median values and statistical analysis was performed using permutation test; data in graphs are represented as individual values, means, and standard errors of the mean.<br>Computational analysis of flow cytometry data.<br>Compensated Flow Cytometry Standard (FCS) 3.0 files were imported into FlowJo software version v10.7.1 and analyzed by standard gating to remove doublets, aggregates and dead cells. The further analysis was performed using CATALYST v1.17.3.<br>Principal Component Analysis (PCA) was executed and visualized in R using the prcomp function (stats v3.6.2) and the pca3d package v0.1. The Euclidean distance of MS-treated groups to HD in PCA space was calculated using the phenoptr v.0.3.2 package.<br>PENCIL v0.7 was used to predict cell clusters associated with absence of breakthrough infection in MS patients and HD. As single cell input data we used our 45-parameter mass cytometry data (scMEP data) analyzed previously with R by using CATALYST v1.18.1 (see method above). We imported into Seurat v4.9.9 58 the expression matrix, containing hyperbolic arcsinh (cofactor 5) transformed data, and the metadata (also UMAP coordinates) of Ag+ T or B lymphocytes.<br>Acquisition software: Cytoflex LX: CytExpert v.2.3

Attune NxT software v.4.2.0
CyTOF software ver. 10.7.1014

For manuscripts utilizing custom algorithms or software that are central to the research but not yet described in published literature, software must be made available to editors and reviewers. We strongly encourage code deposition in a community repository (e.g. GitHub). See the Nature Portfolio guidelines for submitting code & software for further information.

Data

Policy information about availability of data

All manuscripts must include a data availability statement. This statement should provide the following information, where applicable:

- Accession codes, unique identifiers, or web links for publicly available datasets
- A description of any restrictions on data availability
- For clinical datasets or third party data, please ensure that the statement adheres to our policy

The original contributions presented in the study are included in the article/Supplementary Material/Source Data File. Further inquiries can be directed to the corresponding author.

Research involving human participants, their data, or biological material

Policy information about studies with human participants or human data. See also policy information about sex, gender (identity/presentation), and sexual orientation and race, ethnicity and racism.

Reporting on sex and gender	Demographic and clinical characteristics of patients are reported in table 1. Sex was indicated. We do not have any information regarding gender.
Reporting on race, ethnicity, or other socially relevant groupings	NA
Population characteristics	MS patients and healthy donors had a median age of 44.0 (interquartile range, IQR: 41.5-48.5), were mostly female (71.7%), with a median disease duration of 14.3 years (IQR: 10.0-17.1). The most common anti-COVID-19 vaccine used was Pfizer-BioNTech (Comirnaty): 68 persons (64.2%), followed by Moderna (Spikevax): 38 persons (35.8%). Median time from the last dose of vaccine to sample collection was 4.4 months (IQR: 3.8-5.3). Demographic and clinical characteristics of 93 MS patients and 13 healthy donors (HD), the type of DMT at the time of vaccination, the type of third dose vaccine and median range of time to last administration, prior COVID-19 infection status, and relevant comorbidities are shown in Supplementary data 1. Patients treated with different DMT were enrolled such as: natalizumab (n=15; 14.2%), DMF (n=18; 17.0%), DMF patients with decreased absolute lymphocyte counts (<800/uL) at the time of sampling, defined “DMF lymphopenic” (n=10; 9.4%), interferon IFN (n=12; 11.3%), FTY (n=14; 11.3%), aCD20 [n=10; 9.4%, which included those treated with ocrelizumab (n=7; 70.0%) or rituximab (n=3; 30.0%)], cladribine (n=6; 5.7%), and teriflunomide (n=8; 7.5%).
Recruitment	We enrolled patients who were admitted to routine visit in the Nerological Center in the clinic whith the following inclusion and exclusion criteria. Patients were eligible for inclusion if they met the following criteria: a) a confirmed diagnosis of Relapsing-Remitting Multiple Sclerosis (RRMS), and b) a history of treatment with FTY, dimethyl fumarate, natalizumab, or teriflunomide for a minimum of six months, or having undergone at least two infusional cycles with rituximab/ocrelizumab or completed at least one full cycle of cladribine. Patients on ocrelizumab or rituximab, as per routine clinical practice, underwent SARS-CoV-2 vaccination at least six weeks before subsequent infusion or at least three months after the last infusion. Exclusion criteria comprised treatment with steroids during the preceding six weeks and a history of COVID-19 before vaccination.
Ethics oversight	The study was reviewed and approved by each participant, including healthy donors, provided informed consent according to Helsinki Declaration, and all uses of human material have been approved by the local Ethical Committee (Comitato Etico dell'Area Vasta Emilia Nord, protocol number 199/ 2022, May 24th, 2020) and by the University Hospital Committee (Direzione Sanitaria dell'Azienda Ospedaliero Universitaria di Modena, protocol number 5974, February 24th, 2023). The patients/participants provided their written informed consent to participate in this study.

Note that full information on the approval of the study protocol must also be provided in the manuscript.

Field-specific reporting

Please select the one below that is the best fit for your research. If you are not sure, read the appropriate sections before making your selection.

☒ Life sciences ☐ Behavioural & social sciences ☐ Ecological, evolutionary & environmental sciences

For a reference copy of the document with all sections, see nature.com/documents/nr-reporting-summary-flat.pdf

Life sciences study design

All studies must disclose on these points even when the disclosure is negative.

Sample size	Due to the situation, i.e., the Covid pandemics, no sample size calculation was performed. However, according to our previous and large experience on the analysis of T cells, a sample size of 12 patients and 12 controls for cell phenotype was considered sufficient to detect relevant differences. However, for analysis of T cell phenotype, and function we were able to include 94 patients and 13 healthy

	donors.Notwithstanding the fact that for some treatment groups we did not reach a numerosity of 12, due to the striking differences in the mechanisms of action of the drugs, we nevertheless also detected significant differences involving treatment groups with <12 patients per group, as reported in the paper, with regard to either antigen-specific T/B cell responses or metabolic features.
Data exclusions	No data were excluded from the analysis.
Replication	No experimental replication was conducted in this study, as all samples were derived from primary human participants. However the reproducibility of the analysis was tested analysing the same samples in different days. Flow cytometers were aligned every day with QC beads.
Randomization	Patients were not randomized. They were enrolled consecutively throughout the enrolment period, if inclusion/exclusion criteria were met, during routine clinical visits at the MS centre. Samples were then allocated to the different groups based on the disease-modifying treatment they were on. The differences in numerosity throughout treatment groups reflect the different frequencies of treatments prescribed per standard clinical practice at our center.<br>Randomization was not carried out since we were not expecting the different treatment groups to be similar in relation to disease or immune cell status. We were, in fact, expecting a different effect of the Sars-CoV2-vaccine as a consequence of the differences between the groups and, in particular, of the different mechanisms of action of the prescribed disease-modifying treatments
Blinding	For obvious reasons, all blood from human beings is considered infected and treated as such, so fersearchers used the same procedures for all samples. So, samples were coded in the Multiple Sclerosis Clinic and taken to the lab, where blood was treated, stored and subsequently analyzed in a blind manner. Moreover, we applied unsupervised statistical analysis to avoid any possible influence of the operator. The senior authors of the paper (Cossarizza) did not open the key until the end of the study.

Reporting for specific materials, systems and methods

We require information from authors about some types of materials, experimental systems and methods used in many studies. Here, indicate whether each material, system or method listed is relevant to your study. If you are not sure if a list item applies to your research, read the appropriate section before selecting a response.

Materials & experimental systems

n/a	Involved in the study
☐	☒ Antibodies
☒	☐ Eukaryotic cell lines
☒	☐ Palaeontology and archaeology
☒	☐ Animals and other organisms
☐	☒ Clinical data
☒	☐ Dual use research of concern
☒	☐ Plants

Methods

n/a	Involved in the study
☒	☐ ChIP-seq
☐	☒ Flow cytometry
☐	☒ MRI-based neuroimaging

Antibodies

Antibodies used

All the antibodies used are reported in supplementary tables 1-5, divided per panel. For each antibody, marker, label, clone, brand cat, lot and titer have been reported.
Antibodies used for AIM assay (supplementary table 1).
Target Dye Clone Producer Catalog number Lot number Titer (µL)/100µL
PromoFluor840 Maleimide N/A Promocell PK-PF840-3- 01 0.3
CD45RA FITC 2H4 Beckman Coulter (DuraClone IM T) B53328 -
CCR7 PE G043H7 Beckman Coulter (DuraClone IM T) B53328 -
CD28 ECD CD28.2 Beckman Coulter (DuraClone IM T) B53328 -
PD-1 PC5.5 PD1.3.5 Beckman Coulter (DuraClone IM T) B53328 -
CD27 PC7 1A4.CD27 Beckman Coulter (DuraClone IM T) B53328 -
CD4 APC 13B8.2 Beckman Coulter (DuraClone IM T) B53328 -
CD8 A700 B9.11 Beckman Coulter (DuraClone IM T) B53328 -
CD3 APC-A750 UCHT-1 Beckman Coulter (DuraClone IM T) B53328 -
CD57 Pacific Blue NC1 Beckman Coulter (DuraClone IM T) B53328 -
CD45 Krome Orange J33 Beckman Coulter (DuraClone IM T) B53328 -
CXCR3 BV785 G025H7 BioLegend 353738 B302668 1.25
CCR6 BUV496 11A9 Becton Dickinson 612948 1114714 1.25
CXCR5 BUV661 RF8B2 Becton Dickinson 741559 1298915 0.6
CD69 BV650 FN50 BioLegend 310934 B346313 2.5
CD137 BUV395 4B4-1 Becton Dickinson 745737 1298922 1.25
CD95 BV605 DX2 BioLegend 305628 B344380 2.5
mAbs used in ICS protocol (Suppllementary table 2)
TARGET DYE CLONE PRODUCER CATALOG NUMBER LOT NUMBER TITER

µL/100µL
LIVE/DEAD AQUA N/A ThermoFisher L34966 2268307 1.25
CD4 AF700 RPA-T4 Biolegend 300526 B336913 0.6
CD8 APC-Cy7 RPA-T8 Biolegend 301016 B300873 0.6
CD3 PE-Cy5 UCHT1 Biolegend 301016 B300873 0.6
IFNg FITC B27 Biolegend 506504 B286029 2.5
TNF BV605 MAb11 Biolegend 502936 B327946 3.75
IL-2 APC MQ1-17H12 Biolegend 500310 B313276 2.5
IL-17a PE-Cy7 BL168 Biolegend 512315 B325831 3.75
GRZB BV421 QA18A28 Biolegend 396414 B311965 2.5
CD107a PE H4A3 Biolegend 328608 B321484 0.3
mAbs used in B cell panel (Supplemetary table 3)
Target Dye Clone Producer Catalog Number Lot Number Titer
µL/100µL
PromoFluor840 Maleimide N/A Promocell PK-PF840-3- 01 0.3
CD45 Krome Orange J33 Beckman Coulter (DuraClone IM B) B53318 -
CD19 ECD J3-119 Beckman Coulter (DuraClone IM B) B53318 -
CD21 PE BL13 Beckman Coulter (DuraClone IM B) B53318 -
CD27 PC7 1A4CD27 Beckman Coulter (DuraClone IM B) B53318 -
CD24 APC ALB9 Beckman Coulter (DuraClone IM B) B53318 -
CD38 APC-A750 LS198-4-3 Beckman Coulter (DuraClone IM B) B53318 -
IgD FITC IA6-2 Beckman Coulter (DuraClone IM B) B53318 -
IgM Pacific Blue SA-DA4 Beckman Coulter (DuraClone IM B) B53318 -
Streptavidin BV650 - BioLegend 405231 B347044 0.3
Streptavidin BUV661 - Becton Dickinson 612979 1188291 0.3
Streptavidin AF700 - ThermoFisher S21383 2286302 0.1
S-protein Biotin - R&D BT10549 DOJH0421071 4.5
CD20 BV785 2H7 BioLegend 302356 B337363 0.6
CD71 BUV395 M-A712 Becton Dickinson 743308 1341511 1.25
IgG BUV496 G18-154 Becton Dickinson 741172 1341490 1.25
IgA PerCP-Vio700 1S11-8E10 Miltenyi Biotec 130-113-478 5211109889 0.5
mAbs used in SCENITH protocol (Supplementary Tabel 4)
Specificity Dye Clone Manufacturer Cat. Lot Titer
µL/100µL
Fc-Block - - Beckton Dickinson 564220 114728 0.5
PromoFluor-840 Maleimide - PromoKine PK-PF840-3-01 429P0-17 0.3
CD19 PE HIB19 Biolegend 302208 B355446 2
CD69 FITC FN50 Biolegend 310904 B347085 2.5
CD4 FITC RPA-T4 Biolegend 300506 B283935 2
CD8 PE RPA-T8 Biolegend 301008 B323647 0.6
CD8 APC-Cy7 RPA-T8 Biolegend 301016 B300873 0.6
CD3 PB UCHT1 Beckton Dickinson 558117 1180049 2.5
CD69 BV650 FN50 Biolegend 310934 B356230 2.5
CD137 BUV395 4B4-1 Beckton Dickinson 745739 2171939 1.25
Puromycin AF647 1/250
mAbs used in CYTOF scMEP panel (Supplementary Table 5)MARKER label clone brand cat lot titer (ul)
CD45 089Y HI30 Standard Biotools 3089003B 2208767-16 1
CD4 106Cd RPA-T4 Biolegend 300502 custom 1
HLA-DR 110Cd L243 Biolegend 307602 custom 1
CD8a 111Cd RPA-T8 Biolegend 301002 custom 1
GAPDH 112Cd 6C5 ThermoFisher AM4300 custom 1
CD20 113Cd 2H7 Biolegend 302302 custom 1
LDHA 114Cd EP1566Y AbCam ab219591 custom 1
NRF2_p 116Cd EP1809Y AbCam ab180844 custom 1
CD3 141Pr UCHT1 Standard Biotools 3141019B 2112251-08 1
CD19 142Nd HIB19 Standard Biotools 3142001B 2203508-10 1
CD45RA 143Nd HI100 Standard Biotools 3143006B 2209433-27 1
CD38 144Nd HIT2 Standard Biotools 3144014B 2203499-09 1
CytC 145Nd 6H2.B4 Biolegend 612302 custom 1
CS 146Nd EPR8067 Abcam ab233838 custom 1
CD11c 147Sm Bu15 Standard Biotools 3147008B 3431914 1
HIF1A 148Nd 700505 Thermofisher 16H4L13 custom 1
PFKFB4 149Sm PA528648 Thermofisher PA5-28648 custom 1
CD134 150Nd ACT35 Standard Biotools 3150023B 2112513-23 1
ACC_p 151Eu D7D11 Cell Signaling 11818S custom 1
CD21 152Sm BL13 Standard Biotools 3152010B 2205540-11 1
ATPA5 153Eu 15H4C4 Abcam ab14748 custom 1
VDAC1 154Sm 20B12AF2 Abcam ab14734 custom 1

	CD36 155Gd 5-271 Standard Biotools 3155012B 1151904 1<br>G6PD 156Gd EPR20668 Abcam ab231828 custom 1<br>CD27 158Gd L128 Standard Biotools 3158010B 2206891-08 1<br>CD98 159Tb UM7F8 Standard Biotools 3159022B 1551501 1<br>CD28 160Gd CD28.2 Standard Biotools 3160003B 2206133-22 1<br>Ki-67 161Dy B56 Standard Biotools 3161007B 2202239-18 1<br>CD69 162Dy FN50 Standard Biotools 3162001B 2112484-22 1<br>PDK1_p 163Dy 2H3AA11 Abcam ab110335 custom 1<br>GLUT1 164Dy EPR3915 Abcam ab196357 custom 1<br>PGC1a_p 165Ho Polyclonal Novus NBP1-04676 custom 1<br>HK2 166Er EPR20839 Abcam ab228819 custom 1<br>CD197 167Er G043H7 Standard Biotools 3167009A 2205601-16 1<br>MCT1 168Er ERR13706(B) Abcam ab250131 custom 1<br>GLUD1/2 169Tm D9F7P Cell Signaling 12793S custom 1<br>IDH1 170Er 843219 Novus MAB7049 custom 1<br>anti-spike 171Yb - R&D 10549-CV DODR0622071 1<br>pS6 [S235/S236] 172Yb A17020B Biolegend 608602 B333307 1<br>CPT1A 173Yb 8F6AE9 Abcam ab128568 custom 1<br>CD279 (PD-1) 174Yb EH12.2H7 Standard Biotools 3155009B 2206216-29 1<br>pHistone H3 [S28] 175Lu HTA28 Standard Biotools 3175012A 2208977-31 1<br>CD57 176Yb HNK-1 Biolegend 359602 B338514 1<br>CD137 209Bi 4B4-1 Standard Biotools 3209015B 2203423-07 1<br>Cell-ID Cisplatin 195Pt - Standard Biotools 201195 2203477-08 0.5<br>Cell-ID Intercalator 191&193Ir - Standard Biotools 201192A 2204312-27<br>Anti-puromycin used in the SCENITH panel has been gifted by Dr. Rafa Arguello.
Validation	We used only monoclonal antibodies that are commercially available and have been validated by different companies. We have titrated each of them for the optimal use by flow cytometry, as recommended by the most recent guidelines for the use of cytometry in immunological studies (Cossarizza et al., Eur. J. Immunol. 2021: Hartmann et al Nat Biotecnology 2022). For more information about antibody validation by different brand visit www.biolegend.com; www.standardbiotools.com; www.bdbiosciences.com; www.abcam.com; www.novus.com; www.cellsignalling.com; www.thermofisher.com; www.beckmancoulter.com.

Clinical data

Policy information about clinical studies

All manuscripts should comply with the ICMJEguidelines for publication of clinical research and a completedCONSORT checklist must be included with all submissions.

Clinical trial registration	NA
Study protocol	NA
Data collection	NA
Outcomes	NA

Plants

Seed stocks	NA
Novel plant genotypes	NA
Authentication	NA

Flow Cytometry

Plots

Confirm that:

- [x] The axis labels state the marker and fluorochrome used (e.g. CD4-FITC).
- [x] The axis scales are clearly visible. Include numbers along axes only for bottom left plot of group (a 'group' is an analysis of identical markers).
- [x] All plots are contour plots with outliers or pseudocolor plots.
- [x] A numerical value for number of cells or percentage (with statistics) is provided.

Methodology

Sample preparation

Blood collection and isolation of mononuclear cells
Up to 30 mL of blood were collected from each patient in vacuettes containing ethylenediamine-tetraacetic acid (EDTA). Blood was immediately processed. Isolation of peripheral blood mononuclear cells (PBMC) was performed using ficoll-hypaque according to standard procedures. For all experiments, except those related to metabolic investigation, PBMC were stored in liquid nitrogen in fetal bovine serum (FBS) supplemented with 10% dimethyl sulfoxide (DMSO). For metabolic investigation, PBMC were used immediately after isolation.
Isolated PBMCs were thawed and rested for 6 hours. After resting, CD40-blocking antibody (0.5 mg/ml final concentration) (Miltenyi Biotec, Bergisch Gladbach, Germany) was added to the cultures 15 min before stimulation. PBMCs were cultured in 96-well plate in the presence of 15-mer peptides with 11-amino acids overlap, covering the complete sequence of Wuhan SARS-CoV-2 Spike glycoprotein (PepTivator SARS-CoV-2 Prot_S complete, Miltenyi Biotec, Bergisch Gladbach, Germany) together with 1 μg/mL of anti-CD28 (Miltenyi Biotec, Germany). PBMCs were stimulated for 18 h at 37 °C in a 5% CO2 atmosphere in complete culture medium (RPMI 1640 supplemented with 10% fetal bovine serum and 1% each of L-glutamine, sodium pyruvate, nonessential amino acids, antibiotics, 0.1M HEPES, 55μM β-mercaptoethanol). For each stimulated sample, an unstimulated one was prepared, as negative control. After stimulation, cells were washed with PBS and stained with PromoFluor IR-840 (Promokine, PromoCell, Heidelberg, Germany) for 20 minutes at room temperature (RT). Next, cells were washed with FACS buffer (PBS supplemented with 2% FBS) and stained with the following fluorochrome-labeled mAbs: CXCR5-BUV661, CCR6-BUV496, CXCR3-BV785 for 30 minutes at 37 °C. Finally, cells were washed with FACS buffer and stained for 20 minutes at RT with Duraclone IM T cell panel (Beckman Coulter, Brea, CA) containing CD45-Krome Orange, CD3-APC-A750, CD4-APC, CD8-AF700, CD27-PC7, CD57-Pacific Blue, CD279 (PD1)-PC5.5, CD28-ECD, CCR7-PE, CD45RA-FITC and added with other three fluorescent mAbs i.e., CD69-BV650, CD137-BUV395 and CD95-BV605. Samples were acquired on a CytoFLEX LX flow cytometer (Beckman Coulter). All reagents used for T cell phenotyping are listed in Supplementary Table 1. All mAbs added to DuraClone IM T cells were previously titrated on human PBMCs and used at the concentration giving the best signal-to-noise ratio. The gating strategies used to identify CD4+ and CD8+ T cells are reported in the Supplementary Figures 1,4.

Detection of SARS-CoV-2-specific B cells
Thawed PBMC were washed twice with RPMI 1640 supplemented with 10% fetal bovine serum and 1% each of L-glutamine, sodium pyruvate, nonessential amino acids, anti- biotics, 0.1M HEPES, 55μM β-mercaptoethanol and 0.02 mg/ml DNAse. PBMC were washed with PBS and stained using viability marker PromoFluor IR-840 (Promokine, PromoCell, Heidelberg, Germany) for 20 min at RT in PBS. Next, cells were washed with PBS and stained for 15 min at RT with streptavidin-AF700 (decoy channel; ThermoFisher Scientific, USA) to remove false positive SARS-CoV-2-specific B cells. After washing with FACS buffer, cells were stained with biotinylated full-length SARS-CoV-2 spike protein (R&D Systems, Minneapolis) labelled with different streptavidin-fluorophore conjugates. Full-length biotinylated spike protein was mixed and incubated with streptavidin-BUV661(Becton Dickinson) or streptavidin-BV650 (BioLegend) at a 6:1 mass ratio for 15 min at RT. All samples were stained with both biotinylated streptavidin for 1h at 4°C. Then, cells were washed with FACS buffer and stained for 20 min at RT with DuraClone IM B cells (Beckman Coulter, Brea, CA) containing the following lyophilized directly conjugated mAbs: anti-IgD-FITC, CD21-PE, CD19-ECD, CD27-PC7, CD24-APC, CD38-AF750, anti-IgM-PB, CD45-KrO to which following drop-in antibodies were added: CD71-BUV395, CD20-BV785, anti-IgG-BUV496 and anti-IgA-PerCP-Vio700. Samples were acquired on a CytoFLEX LX flow cytometer (Beckman Coulter). A minimum of 1,000,000 cells per sample were acquired. All reagents used for B cell phenotype are reported in Supplementary Table 3. All mAbs added to DuraClone IM B cells were previously titrated on human PBMCs and used at the concentration giving the best signal-to-noise ratio. The gating strategy used to identify Ag– and Ag+ B cells is reported in the Supplementary Figure 11.

Intracellular cytokine staining (ICS)
Isolated PBMCs were thawed and rested for 6 h. PBMCs were stimulated in the presence of a pool of lyophilized peptides covering the complete protein coding sequence (aa 5–1273) of spike glycoprotein ("S") of SARS-CoV-2 (PepTivator SARS-CoV-2 Prot_S Complete Miltenyi Biotec, Bergisch Gladbach, Germany) together with 1 μg/ml of anti-CD28/49d (Becton Dickinson). PBMCs were stimulated for 16 h at 37°C in a 5% CO2 atmosphere in complete culture medium (RPMI 1640 supplemented with 10% FBS and 1% each of L-glutamine, sodium pyruvate, non-essential amino acids, antibiotics, 0.1 M HEPES, 55 mM β-mercaptoethanol, and 0.02 mg/mL DNAse I). For each stimulated sample, an unstimulated one was prepared as a negative control. All samples were incubated with protein transport inhibitors brefeldin A (Golgi Plug, Becton Dickinson Bioscience, San Jose, CA, USA) and monensin (Golgi Stop, Becton Dickinson Bioscience, San Jose, CA, USA) and previously titrated concentration of CD107a-PE (BioLegend, San Diego, CA, USA). After stimulation, cells were washed with PBS and stained with LIVE/DEAD fixable Aqua (ThermoFisher Scientific, USA) for 20 min at RT. Next, cells were washed with FACS buffer and stained with surface mAbs recognizing CD3-PE.Cy5, CD4-AF700, and CD8-APC.Cy7 (BioLegend, San Diego, CA, USA). Cells were washed with FACS buffer and fixed and permeabilized with the Cytofix/Cytoperm buffer set (Becton Dickinson Bioscience, San Jose, CA, USA) for cytokine detection. Then, cells were stained with previously titrated mAbs recognizing IL-17-PE-Cy7, TNF-BV605, IFN--FITC,   IL-2-APC, and GRZMB BV421 (all mAbs from BioLegend, San Diego, CA,

USA). Samples were acquired on an Attune NxT acoustic cytometer (ThermoFisher Scientific, USA). Supplementary Table 2 reports mAb titers, clones, catalog numbers, and type of fluorochrome used in the panel.
Gating strategy used to identify and analyze the intracellular cytokine production of CD4+ and CD8+ T lymphocytes is reported in Supplementary Figure 7.

Instrument	CytoFLEX LX flow cytometer (Beckman Coulter) for T cell phenotype.<br>Attune NxT acoustic cytometer (ThermoFisher) for intracellular staining.<br>Cytof XT Helios for scMEP.
Software	We have used the following softwares:<br>Attune NxT 3.2.1 software (ThermoFisher)<br>Cytexpert 2.4 software (Beckman Coulter)<br>CytoBank (Beckman Coulter)<br>FlowJo software version 9 (Becton Dickinson)<br>R statistical packages (CATALYST 1.10.1). The script is available at: https://github.com/HelenaLC/CATALYST<br>CyTOF® Software v9.0<br>FlowSOM (Bioconductor)<br>Simplified Presentation of Incredibly Complex Evaluation (SPICE) software
Cell population abundance	No cell sorting has been performed.
Gating strategy	Supplementary Figure 1 Gating strategy for the identification and characterization of antigen-specific CD4+ T cells (AIM assay). (a) A gate was set on CD69 vs TIME plot, then in this population, a gate was set according to physical parameter (FSC and SSC. Further gating is done in an FSC-H and FSC-Width dot plot to eliminate doublets. On a bivariate plot of CD45 vs. ViaKrome (viability) select CD45+, ViaKrome– cells (viable cells). On a bivariate plot of CD3 vs SSC-H select CD3 T lymphocytes. CD4+ T cells was selected and the percentage of Antigen-Specific (CD69+ CD137+)T cells was quantified. (b) Gating strategy to identify and characterize i)T helper (Th), ii) circulating T follicular helper (cTfh), iii) Naive, true naive and TSCM among Ag +CD4+ T cell populations. EM, effector memory; CM central memory; EMRA, terminally differentiated effector memory; TSCM stem memory cell.<br>Supplementary Figure 4. Gating strategy for the identification and characterization of antigen-specific CD8+ T cells (AIM assay). (a) A gate was set on CD69 vs TIME plot, then in this population, a gate was set according to physical parameter (FSC and SSC. Further gating is done in an FSC-H and FSC-Width dot plot to eliminate doublets. On a bivariate plot of CD45 vs. ViaKrome (viability) select CD45+, ViaKrome– cells (viable cells). On a bivariate plot of CD3 vs SSC-H select CD3 T lymphocytes. CD4+ T cells was selected and the percentage of Antigen-Specific (CD69+ CD137+)T cells was quantified. (b) Gating strategy to identify and characterize i)T cytotoxic (Tc), ii) Tc CXCR5+, iii) Naive, true naive and TSCM among Ag+CD4+ T cell populations. EM, effector memory; CM central memory; EMRA, terminally differentiated effector memory; TSCM stem memory cell.<br>Supplementary Figure 7. Gating strategy and representative plots of intracellular staining analysis of cytokine producing cells (ICS) after overnight stimulation with spike protein compared to unstimulated control. Cytokine production and polyfunctionality of antigen-specific CD4+ T cells (panel b) and CD8+ T cells (panel c) . Numbers in the dot plots indicate the percentage of CD4+ and CD8+ cells identified by the gates. Comparison between the total production of IFN-g, TNF, IL-17, IL-2, CD107a, and GZMB .<br>Supplementary Figure 20. (a) Gating strategy of antigen-specific T cells. On a bivariate plot of Time vs. CD3, create and place a rectangular region to include all valid events acquired in chronologic homogeneity and avoid fluidic perturbances. Forward and side scatter (FSC and SSC) gating is used to identify cells of interest based on the relative size and complexity of the cells, while removing debris and other events that are not of interest. Further gating is done in an FSC-H and FSC-Width dot plot to eliminate doublets. On a bivariate plot of CD3 vs. Promokine (viability) select CD3+, Promokine– cells (viable T cells). On a bivariate plot of CD4 vs CD8 select CD8+ or CD4+ T cells and evaluate CD69+ CD137+ Antigen Specific T cells. Within CD69+ CD137+ T cells evaluate the MFI of Puromycin-AF647 after the treatment of different metabolic inhibitors.

☒ Tick this box to confirm that a figure exemplifying the gating strategy is provided in the Supplementary Information.

Magnetic resonance imaging

Experimental design

Design type	NA
Design specifications	NA
Behavioral performance measures	NA

Acquisition

Imaging type(s)	NA
Field strength	NA
Sequence & imaging parameters	NA
Area of acquisition	NA

Diffusion MRI ☐ Used ☒ Not used

Preprocessing

Preprocessing software	NA
Normalization	NA
Normalization template	NA
Noise and artifact removal	NA
Volume censoring	NA

Statistical modeling & inference

Model type and settings	NA
Effect(s) tested	NA

Specify type of analysis: ☐ Whole brain ☐ ROI-based ☐ Both

Statistic type for inference (See Eklund et al. 2016)	NA
Correction	NA

Models & analysis

n/a	Involved in the study
☒	☐ Functional and/or effective connectivity
☒	☐ Graph analysis
☒	☐ Multivariate modeling or predictive analysis
